# A multi-omics framework integrating gut microbiota, blood metabolites, and immune cells to elucidate the pathogenesis of Alzheimer’s disease

**DOI:** 10.3389/fimmu.2026.1842398

**Published:** 2026-07-06

**Authors:** Bei Wang, Wei Yan, Yusheng Zhang

**Affiliations:** Naval Medical Centre, Naval Medical University, Shanghai, China

**Keywords:** Alzheimer’s disease, machine learning, Mendelian randomization, microbiota-metabolite-immune axis, multi-omics integration

## Abstract

**Background:**

Alzheimer’s disease (AD) develops through complex interactions between the central nervous system and peripheral systems. The microbiota-metabolite-immune axis has emerged as an important focus of AD research. However, the coordinated mechanisms that regulate this axis remain poorly understood.

**Methods:**

We used a multi-stage, multi-omics strategy to systematically investigate peripheral–central interactions in AD. The analytical framework integrated Mendelian randomization (MR), summary-data-based Mendelian randomization (SMR), differential expression analysis, machine learning, single-cell and spatial transcriptomics, and quantitative real-time polymerase chain reaction (qPCR) trend confirmation.

**Results:**

Exploratory MR analyses identified multiple microbial taxa, metabolites, and immune cell phenotypes showing associations consistent with potential causal effects on AD. Integrating the SMR and MR findings with differential expression analysis led to the identification of 31 core genetically associated genes. A five-gene predictive model comprising *ATF7IP2*, *TWSG1*, *PTPRN2*, *ASCC3* and *IGF1R* was then developed using machine learning. The diagnostic potential of the individual feature genes was further evaluated in an external validation dataset. Spatial transcriptomic analyses revealed clear cell type-specific expression patterns in brain tissue, with *IGF1R*, *ASCC3*and *TWSG1* showing potential co-localization in oligodendrocytes. qPCR trend confirmation in pooled samples produced expression trends consistent with the directions inferred from eQTL-based MR.

**Conclusions:**

This study mapped a regulatory network underlying the AD microbiota–metabolite-immune-brain axis and identified core genes with potential diagnostic and therapeutic value. The spatial transcriptomic findings, while primarily based on *in situ* co-localization analysis, highlight a biologically plausible but provisional working hypothesis regarding an active role for oligodendrocytes in AD pathology. Overall, this study supports a systems-level view of AD that may inform precision medicine strategies.

## Highlights

- A multi-omics framework integrating gut microbiota, metabolites, and immune cells reveals peripheral-central interactions in Alzheimer’s disease (AD).
- Exploratory Mendelian randomization identifies associations consistent with potential causal effects between specific microbial taxa, metabolic traits, and immune phenotypes with AD risk.
- A five-gene predictive model (*ATF7IP2*, *TWSG1*, *PTPRN2*, *ASCC3*, *IGF1R*) is developed, and the diagnostic potential of the individual feature genes is evaluated in external datasets.
- Spatial transcriptomics reveal cell type-specific expression, with *IGF1R*, *ASCC3*, and *TWSG1* co-localizing in oligodendrocytes.
- Spatial transcriptomics highlight a provisional working hypothesis for oligodendrocytes as active participants in AD pathology.

## Introduction

Alzheimer’s disease (AD) is the most common neurodegenerative disorder and is characterized clinically by progressive memory loss, cognitive decline, and behavioral changes. It accounts for approximately 60%-80% of all dementia cases (1). As the global population ages, AD has become a major public health challenge. Although substantial progress has been made over the past three decades in understanding its pathology and developing therapies, current treatments remain limited, and their efficacy and safety are still debated (2). The hallmark pathological features of AD include amyloid-β (Aβ) deposition, neurofibrillary tangles formed by hyperphosphorylated tau, and neuroinflammation (3). While these features are central to disease progression, the upstream triggers and downstream regulatory networks that drive them remain incompletely understood (4, 5).

Recent evidence suggests that AD is not solely a disorder of the central nervous system. Instead, its onset and progression appear to involve extensive communication between the brain and peripheral systems. In this context, the microbiota-metabolite-immune axis has become a major focus of research because it may connect the peripheral environment to brain function. Gut microbiota, blood metabolites, and immune cells interact continuously and form a dynamic regulatory network. Gut microbes produce a wide range of bioactive molecules through the metabolism of dietary components. Once these molecules enter the circulation, they can alter immune cell function, and activated immune cells can then affect the central nervous system through multiple pathways. A clearer understanding of this axis may therefore help explain how AD develops.

The gut-brain connection has drawn particular attention in AD research (6). The gut microbiota communicates bidirectionally with the central nervous system through neural, endocrine, and metabolic routes, and disturbances in microbial balance have been linked to neuroinflammation, Aβ deposition, and tau hyperphosphorylation (7). Changes in the gut microbiota can be detected early in the disease course and may serve as biomarkers or modulators of the brain microenvironment by affecting metabolites such as short-chain fatty acids, bile acids, and tryptophan derivatives (8, 9). Blood metabolites are also informative because they reflect both systemic metabolism and processes related to the central nervous system (10). Metabolomic studies have shown that AD is associated with disturbances in amino acid, lipid, and energy metabolism (11, 12). These metabolic changes may result from brain pathology, but they may also contribute to disease progression through the gut-brain axis, creating a bidirectional feedback loop (13). At the same time, the immune system has a central role in AD. Immune cells, including microglia, T cells, B cells, monocytes/macrophages, and neutrophils, influence neuroinflammation, Aβ clearance, tau spread, and cognitive decline through complex interactions (14, 15).

Although each of these areas has been studied extensively, the interplay among gut microbiota, metabolites, and immune cells in AD has not been systematically characterized. Most previous studies have focused on only one layer of this system, making it difficult to define potential causal relationships or build an integrated view of the regulatory network involved.

In this study, we adopted a multi-stage, multi-omics strategy. First, we focused on potential causal inference. Using two-sample Mendelian randomization (MR), we evaluated the exploratory associations of gut microbial composition, circulating metabolites, and immune cell phenotypes with AD. We then intersected genes identified by MR and summary-data-based Mendelian randomization (SMR) and combined these results with differential expression analysis to identify candidate genetically associated genes. Second, we constructed and validated a predictive model. Four machine learning algorithms were used to select feature genes, build an AD risk model, and visualize risk with a nomogram, and the diagnostic potential of the individual feature genes was further evaluated in an external validation dataset. Third, we examined cell type localization using single-cell RNA sequencing data. Fourth, we added spatial and temporal context by analyzing spatial transcriptomic data and pseudotime trajectory analysis during disease progression. Finally, we confirmed the findings by performing eQTL-based MR for the target genes and measuring gene expression in pooled clinical blood samples using qPCR. Together, these steps created a closed loop from computational inference to experimental confirmation. Through this multi-layered framework, we aimed to clarify the molecular basis of peripheral–central interactions in AD, identify candidate biomarkers for early diagnosis and intervention, and provide a foundation for precision medicine.

## Materials and methods

### Data sources

Genetic association data for AD were obtained from the IEU Open GWAS database (finn-b-G6_ALZHEIMER, https://opengwas.io/datasets/). Genetic association data for blood metabolites were derived from the metabolomics GWAS of the Canadian Longitudinal Study on Aging published by Chen et al. In that study, plasma samples from 8,299 individuals of European ancestry were profiled quantitatively for 1,458 metabolites. After quality control and preprocessing, 1,091 metabolites were retained for GWAS analysis, including 850 known metabolites across eight super-pathways (lipids, amino acids, xenobiotics, nucleotides, cofactors and vitamins, carbohydrates, peptides, and energy metabolism) and 241 unknown or partially characterized molecules. In addition, 309 metabolite ratios were constructed from metabolites that share enzymes or transporters in the Human Metabolome Database (16). Genetic data for immune cell phenotypes were obtained from the SardiNIA study published by Orrù et al. In that study, peripheral blood samples from 3,757 individuals of European ancestry from Sardinia were analyzed by flow cytometry using a BD FACSCanto II flow cytometer and BD FACSDiva software. A total of 731 immune cell phenotypes were quantified, including 118 absolute cell counts, median fluorescence intensities of 389 surface antigens, 32 morphological parameters, and 192 cell ratios (17). Genetic instruments for gut microbial composition were obtained from large-scale microbiome GWAS summary statistics released by the Dutch Microbiome Project. In that study, fecal samples from 7,738 Dutch individuals of European ancestry underwent metagenomic shotgun sequencing. This yielded 207 microbial taxonomic units for genetic analysis, including 5 phyla, 10 classes, 13 orders, 26 families, 48 genera, and 105 species, as well as 205 bacterial functional pathways (18).

Exposure data for SMR analysis were obtained from blood cis-eQTL summary statistics generated by the eQTLGen Consortium. AD-related transcriptomic datasets (GSE138260, GSE37263, GSE29378, GSE5281and GSE36980) were downloaded from the Gene Expression Omnibus (GEO) database(https://www.ncbi.nlm.nih.gov/geo/) and included 143 AD cases and 133 controls. GSE36980 was processed independently as the external validation dataset. Single-cell transcriptomic data were derived from a published scRNA-seq study of brain tissue from patients with AD and controls. Prefrontal cortex samples were obtained from GEO (GSE157827). Spatial transcriptomic data from the human middle temporal gyrus, including samples from patients with AD and controls, were also retrieved from GEO (GSE220442). To evaluate the potential causal association between target gene expression and AD, expression quantitative trait loci (eQTLs) for the target genes were obtained from the Open GWAS database: *ATF7IP2* (eqtl-a-ENSG00000166669), *TWSG1* (eqtl-a-ENSG00000128791), *PTPRN2* (eqtl-a-ENSG00000155093), *ASCC3* (eqtl-a-ENSG00000112249), and *IGF1R* (eqtl-a-ENSG00000140443).

### Mendelian randomization analysis

All MR analyses were performed in R (4.4.3) using the Two Sample MR package (v0.7.0). To ensure the robustness and validity of our instrumental variables (IVs), we applied a rigorous, multi-step selection pipeline: (1) Significance Threshold: SNPs were selected based on a genome-wide significance threshold of *P* < 5×10^-8^; for the respective exposures; (2) Independence: Linkage disequilibrium clumping was performed with a threshold of r² < 0.001 and a window size of 10,000 kb; (3) Exclusion Restriction: To prevent horizontal pleiotropy, we strictly filtered out any SNPs directly associated with the outcome at a nominal significance level (*P* < 0.05). All retained IVs had an F-statistic > 10, mitigating weak instrument bias. Given the high-throughput nature of screening 201 microbial taxa, 731 immune phenotypes, and 1,400 metabolites, applying strict multiple testing corrections (such as Bonferroni or False Discovery Rate) would severely penalize and potentially eliminate true biological signals. Therefore, we deliberately evaluated the initial MR associations using a nominal significance (*P* < 0.05) to serve as a dimensional reduction tool. These initial results are explicitly defined as exploratory findings, with definitive identification relying on downstream multi-omics integration.

The primary potential causal effect was estimated using the random-effects inverse-variance weighted (IVW) method. This method combines the Wald ratio estimates for individual SNPs into an overall potential causal estimate and provides the most precise inference when all instruments are valid. Several additional methods that are more robust to pleiotropy were used as sensitivity analyses: (1) MR-Egger regression, which tests for directional horizontal pleiotropy through an intercept term and provides a pleiotropy-adjusted potential causal estimate; (2) the weighted median method, which gives a consistent estimate if at least 50% of the instrumental variables are valid; and (3) the simple mode and weighted mode methods, which use clustering-based approaches to generate pleiotropy-robust estimates. Furthermore, the raw data for heterogeneity and pleiotropy analyses are provided in **Supplementary Tables 1**, **2**.

**Figure 1:**
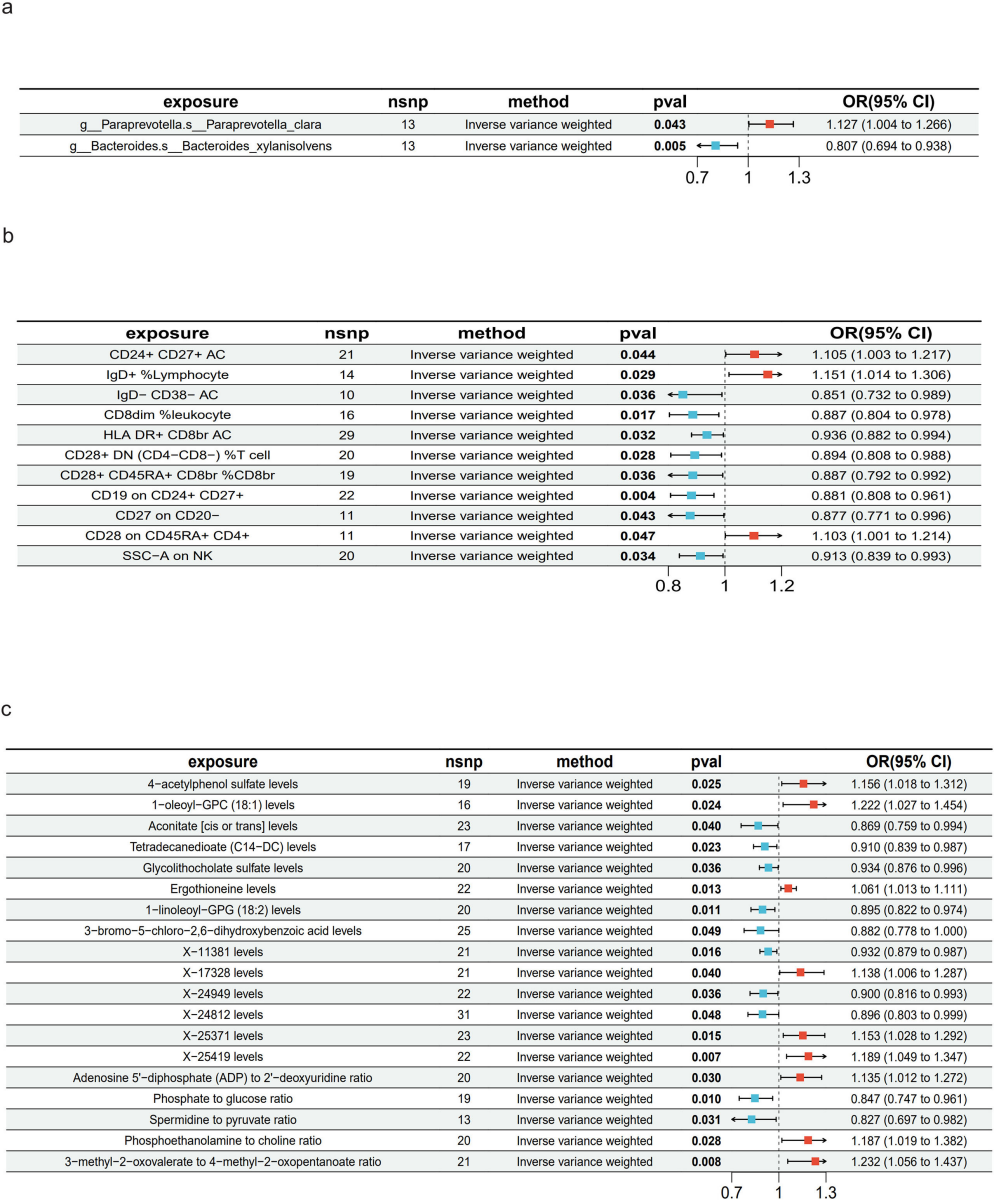
Mendelian randomization analysis of gut microbiota, immune cell phenotypes, and blood metabolites in relation to Alzheimer’s disease risk. Forest plots show associations consistent with potential causal effects (*P* < 0.05) between **(a)** gut microbial taxa, **(b)** immune cell phenotypes, and **(c)** blood metabolites and AD risk. Odds ratios (ORs) and 95% confidence intervals (CIs) are shown. All instrumental variables had F statistics >10, and sensitivity analyses (MR-Egger, weighted median, and MR-PRESSO) detected no evidence of significant horizontal pleiotropy.

Following the initial high-throughput MR screening, exposures demonstrating associations consistent with potential causal effects (*P* < 0.05) with AD were identified as candidate risk factors. To rigorously assess the robustness of these positive findings, multiple sensitivity analyses were conducted on this subset. The MR-PRESSO global test was employed to detect overall horizontal pleiotropy, identify potential outlier SNPs, and correct for pleiotropic bias (**Supplementary Table 3**). For exposures that showed heterogeneity, leave-one-out analyses were performed by removing each SNP in turn to determine whether the overall estimate was driven by a single variant (**Figure 1**).

### SMR analysis

SMR analysis was performed using the SMR software package (v1.3.1). For each gene, the most significant cis-eQTL was used as the instrumental variable, and the potential causal effect of gene expression on AD risk was estimated using the Wald ratio method. Bonferroni correction was applied to control for multiple testing. Based on the number of effective genes tested, the significance threshold was set at *P* < 2.7 × 10^−6^. Probe-to-gene mapping was performed using Strawberry Perl (v5.30.01),

### Differential expression analysis

**Figure 2:**
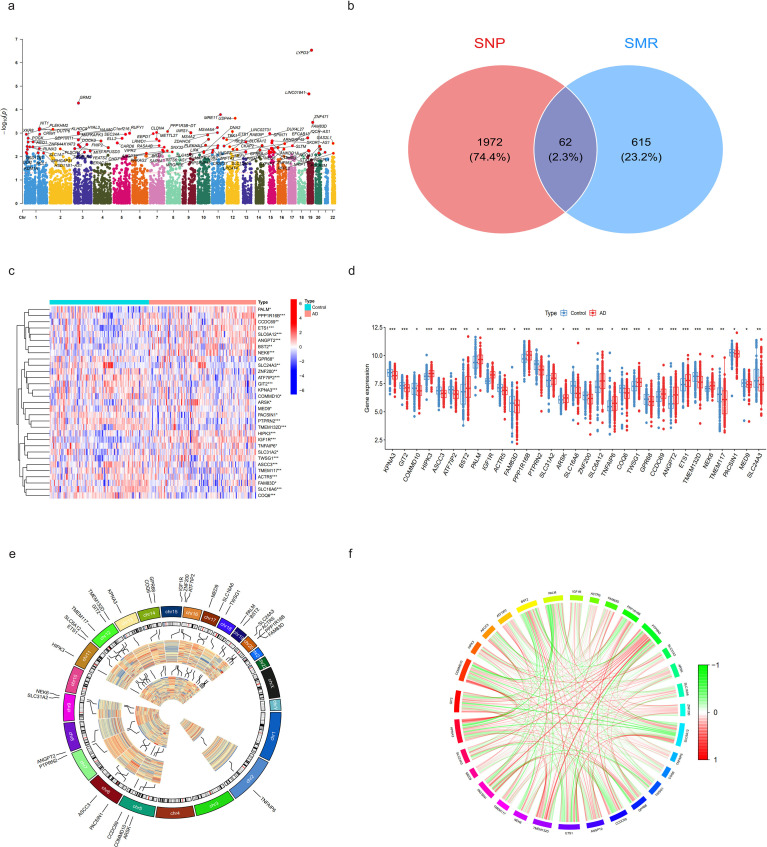
Integrative SMR and MR analyses identify core potential causal genes associated with AD and their differential expression. **(a)** Manhattan plot showing chromosome-wide associations between gene expression and AD risk identified by SMR analysis. **(b)** Venn diagram showing the 61 genes shared between the SMR and MR analyses. **(c)** Heatmap showing clustering of the differential expression patterns of the 31 core genes in the AD and control groups. **(d)** Box plots showing expression differences of the 31 core genes between the two groups. **(e)** Chromosomal ideogram showing the distribution of the 31 core genes across chromosomes. **(f)** Gene co-expression network showing correlations among the core genes (red, positive correlation; green, negative correlation). * P < 0.05, ** P < 0.01, *** P < 0.001.

The limma package (v3.62.2) was used to compare gene expression between AD and control samples in the training set, which combined the GSE138260, GSE37263, GSE29378and GSE5281 datasets. Cross-dataset batch effects were corrected using the ‘Com Bat’ function from the sva (v3.54.0) package (**Figure 2**). Differentially expressed genes were defined as those meeting both criteria: |log_2_FC| > 0.5 and false discovery rate < 0.05.

### Immune cell infiltration analysis

The CIBERSORT algorithm was used to estimate the relative abundance of 22 immune cell types in the training set. Although the CIBERSORT LM22 reference matrix was originally derived from peripheral blood, it has been widely applied to non-blood tissues, including brain transcriptomes, to capture broad immune signatures and infer the presence of infiltrating peripheral immune cells. In this study, we employed this deconvolution strategy to provide a proxy for the neuroimmune microenvironment in AD, recognizing that while it may not perfectly distinguish all brain-resident phenotypes, it offers valuable insights into the systemic-to-central immune crosstalk. Pvalues were calculated using 1,000 permutations, and only samples with CIBERSORT *P* < 0.05 were retained for further analysis. Differences in immune cell abundance between the AD and control groups were assessed using the Wilcoxon rank-sum test. Spearman correlation analysis was then performed to evaluate associations between immune cell abundance and the expression of feature genes. Correlation heatmaps were generated with the pheatmap package (v1.0.13).

### Machine learning model construction

Using the training set, four classification models were developed: random forest (RF; v4.7-1.2), support vector machine (SVM), generalized linear model (GLM), and extreme gradient boosting (XGBoost; v1.2.0.1). Disease status was used as the outcome, and differentially expressed genes were used as input features. Model performance was evaluated with ten-fold cross-validation. The area under the receiver operating characteristic curve (AUC) was the primary performance metric, and accuracy, sensitivity, specificity, and F1 score were also calculated. Based on the optimal feature set, an AD risk prediction model was built using multivariable logistic regression and displayed as a nomogram. Calibration curves were used to assess agreement between predicted and observed probabilities, and decision curve analysis was used to estimate clinical net benefit. XGBoost was ultimately selected because it achieved the highest area under the ROC curve among the four models. Furthermore, residual analysis indicated it had the most concentrated residual distribution and the lowest root mean square error (RMSE). Using the built-in feature importance ranking of XGBoost, the top 5 genes were selected to construct the final AD risk prediction model.

### Single-cell RNA sequencing data analysis

The scRNA-seq data were analyzed using a standard workflow implemented in the Seurat package (v5.4). Quality control filtering was performed using the percentage of mitochondrial genes, percentage of ribosomal genes, number of detected genes, and number of unique molecular identifiers per cell. Cells with mitochondrial gene content <20% and more than 20 detected genes were retained. Data were normalized using the Log Normalize method. Dimensionality reduction was then performed by principal component analysis, and the top 20 principal components were selected for downstream analysis. A k-nearest-neighbor graph was constructed from the selected principal components, and clustering was performed across multiple resolutions (0.1-1.0, increment 0.1). The clustree package (v0.5.1) was used to visualize cluster relationships across resolutions, and a resolution of 0.2 was selected. UMAP was used for nonlinear dimensionality reduction and visualization. Automated cell type annotation was performed with the SingleR package (v2.8.0) using the Human Primary Cell Atlas Data reference. Annotation was performed at the cluster level to improve robustness, and the resulting labels were then mapped back to individual cells. For each gene, the proportion of expressing cells and the average expression level across cell types were summarized with bubble plots.

### Spatial transcriptomic data analysis

Spatial transcriptomic analyses were performed in R using Seurat for data processing and clustering, Matrix for sparse matrix handling, ggplot2 (v4.0.1) and patchwork (v 1.3.2) for visualization, SingleR (v2.8.0) and celldex (v1.20.0) for automated cell type annotation, monocle3 (v1.4.26) and Seurat Wrappers for pseudotime trajectory analysis, and glmGamPoi (v1.22.0) to accelerate SCTransform normalization (v0.4.2).

### qPCR trend confirmation in pooled PBMC samples

#### Study participants and sample collection

The blood samples used in this study were obtained from the clinical laboratory of the Naval Medical Centre, Naval Medical University. All samples were completely deidentified and did not contain any patient identifiers. According to local Chinese legislation, a formal ethical review was not required because no human participants were directly recruited and all samples were fully anonymized. Consequently, an ethical exemption was granted for this study (Exemption No. 2026061801). All procedures were conducted in accordance with local legislation and institutional requirements. The study included 5 patients with a clinical diagnosis of AD and 5 healthy controls. Detailed baseline demographic and clinical characteristics of the participants are provided in **Supplementary Table 4**. AD was diagnosed according to the NIA-AA 2011 criteria. Exclusion criteria included other forms of dementia, severe cerebrovascular disease, autoimmune disease, malignant tumors, recent infection, and recent use of antibiotics or immunomodulatory agents. The study followed the Declaration of Helsinki, and written informed consent was obtained from all participants. Five milliliters of fasting venous blood were collected into EDTA anticoagulant tubes, stored temporarily at 4 °C, and processed within 2 h for isolation of peripheral blood mononuclear cells (PBMCs). PBMCs were isolated by Ficoll density-gradient centrifugation, and the cell pellets were stored at −80 °C until use. To minimize individual genetic and environmental noise and confirm the overarching population-level expression trends inferred from the computational analyses, we utilized an RNA pooling strategy. Equal amounts of total RNA from the 5 strictly matched AD patients and 5 healthy controls were pooled into an “AD group” and a “Control group,” respectively. Quantitative PCR was then performed on these pooled samples with three technical replicates. Exact forward and reverse primer sequences used for the qPCR trend confirmation are provided in **Supplementary Table 5**.

### Major instruments and reagents

Quantitative real-time PCR was performed using a TGL-16 benchtop high-speed refrigerated centrifuge (Xiangyi, China), a Micro Drop microvolume spectrophotometer (Shanghai Baoyude, China), a My Cycler thermal cycler (Bio-Rad, USA), and a ViiA7 real-time PCR system (ABI, USA). Reagents included RNAiso Plus total RNA extraction reagent (TAKARA, Japan; Cat. No. 9109), Prime Script RT Master Mix reverse transcription kit (TAKARA, Japan; Cat. No. RR037A), and Perfect Start Universal Green qPCR Super Mix (Beijing Trans Gen Biotech, China; Cat. No. AQ631). Primers were synthesized by Tsingke Biotechnology Co., Ltd. (Beijing, China).

### Total RNA extraction

Total RNA was extracted from PBMCs using a TRIzol-based protocol. Briefly, 1 mL of RNAiso Plus reagent was added to each cell pellet, and the sample was pipetted repeatedly until lysis was complete. The lysate was incubated at room temperature for 5 min. Chloroform (0.2 mL per 1 mL RNAiso Plus) was then added, and the mixture was shaken vigorously for 15 s, incubated at room temperature for 3 min, and centrifuged at 12,000 rpm for 15 min at 4 °C. The upper aqueous phase was transferred to a new 1.5mL tube and mixed with an equal volume of isopropanol (approximately 0.5 mL). After incubation at room temperature for 10 min, the sample was centrifuged at 12,000 rpm for 10 min at 4 °C. The supernatant was discarded, and the pellet was washed with 1 mL of 75% ethanol and centrifuged at 7,500 rpm for 5 min at 4 °C. The supernatant was removed, and the RNA pellet was air-dried for approximately 5 min before being dissolved in an appropriate volume of DEPC-treated water.

RNA concentration and purity were measured using a Micro Drop microvolume spectrophotometer, and the A260/A280 ratio was recorded. Samples with an A260/A280 ratio between 1.8 and 2.0 were considered acceptable.

### Reverse transcription

Reverse transcription was performed using the Prime Script RT Master Mix kit to generate cDNA from total RNA. Reactions were prepared on ice in a total volume of 10μL and contained 2μL 5× Prime Script Buffer (for Real Time), 0.5μL Prime Script RT Enzyme Mix I, 0.5μL oligo(dT) primer (50μM), 0.5μL random 6-mers (100μM), 600 ng total RNA, and RNase-free dH_2_O to volume. To improve accuracy, the reaction mix was prepared in slight excess, and RNA samples were added last. The reverse transcription program was 37 °C for 15 min followed by 85 °C for 5 s. The resulting cDNA was stored at −20 °C until use.

### Quantitative PCR

Real-time quantitative PCR was performed using a SYBR Green-based assay. Primer sequences were designed with Primer Premier 5.0, and specificity was checked using NCBI Primer-BLAST. GAPDH was used as the internal reference gene. qPCR was performed on an ABI ViiA7 real-time PCR system using the Perfect Start Universal Green qPCR Super Mix kit. Reactions were prepared on ice in a total volume of 10μL and contained 5μL 2× Perfect Start Universal Green qPCR Super Mix, 0.2μL each of forward and reverse primers (10μM; final concentration 0.2μM), 2μL of 10-fold diluted cDNA template, and RNase-free water to volume. Three technical replicates were run for each sample, and no-template controls were included. The amplification protocol consisted of an initial denaturation at 95 °C for 1 min, followed by 40 cycles of 95 °C for 5 s and 60 °C for 30 s, during which fluorescence was collected. Melt-curve analysis was performed at the end of the run by gradually increasing the temperature from 60°C to 95 °C to confirm amplification specificity.

### qPCR data analysis

Relative gene expression was quantified using the comparative threshold cycle method.

(2^(-ΔΔCt)), where ΔCt = Ct_target − Ct_GAPDH. ΔΔCt values were calculated by subtracting the ΔCt of the pooled control sample from the ΔCt of the pooled AD sample. Fold change was determined as 2^(-ΔΔCt), with values above 1.0 indicating an upward trend and below 1.0 indicating a downward trend in the pooled AD sample.

### Statistical analysis

Unless otherwise specified, all statistical analyses were performed in R (4.4.3). The Shapiro-Wilk test was used to assess the normality of continuous variables. Normally distributed variables are presented as mean ± standard deviation and were compared between groups using the independent-samples t test. Non-normally distributed variables are presented as median (interquartile range) and were compared using the Mann-Whitney U test. Categorical variables are presented as counts and percentages and were compared using the chi-square test or Fisher’s exact test, as appropriate. Correlations were assessed using Spearman’s rank correlation coefficient. All tests were two-sided, and *P* < 0.05 was considered statistically significant.

## Results

### Mendelian randomization analysis

To systematically evaluate the exploratory relationships of gut microbiota, immune cell phenotypes, and blood metabolites with AD, we performed two-sample MR analyses. All instrumental variables had F statistics >10, indicating no evidence of weak-instrument bias. Sensitivity analyses, including MR-Egger, weighted median, and MR-PRESSO, produced consistent results and detected no significant horizontal pleiotropy (all *P* > 0.05). The statistically significant exploratory associations (*P* < 0.05) are summarized below.

MR analysis of 201 microbial taxonomic units identified two species-level microbial traits associated with AD (**Figure 1**). A higher relative abundance of Paraprevotella clara (P. clara) was associated with an increased risk of AD (OR = 1.127, 95% CI: 1.004-1.266, *P* = 0.043), suggesting that this species may be a risk factor. In contrast, a higher relative abundance of Bacteroides xylanisolvens (B. xylanisolvens) was associated with a lower risk of AD (OR = 0.807, 95% CI: 0.694-0.938, *P* = 0.005), indicating a potential protective effect.

MR analysis of 731 immune cell phenotypes identified several immune features associated with AD (**Figure 1**). Among B cell-related phenotypes, a higher absolute count of CD24+ CD27+ cells was associated with increased AD risk (OR = 1.105, 95% CI: 1.003-1.217, *P* = 0.044), whereas the absolute count of IgD- CD38- cells showed a protective association (OR = 0.851, 95% CI: 0.732–0.989, P = 0.036). In addition, a higher proportion of IgD+ lymphocytes was associated with increased AD risk (OR = 1.151, 95% CI: 1.014-1.306, *P* = 0.029).

Among T cell-related phenotypes, both the proportion of CD28+ double-negative (DN; CD4−CD8−) T cells (OR = 0.894, 95% CI: 0.808–0.988, P = 0.028) and the proportion of CD28+ CD45RA+ CD8^br T cells among CD8^br cells (OR = 0.887, 95% CI: 0.792–0.992, P = 0.036) were inversely associated with AD risk, suggesting protective effects. By contrast, increased CD28 expression on CD45RA+ CD4+ T cells was associated with higher AD risk (OR = 1.103, 95% CI: 1.001–1.214, P = 0.047).

With respect to cell surface markers, the proportion of CD8dim cells among leukocytes (OR = 0.887, 95% CI: 0.804-0.978, *P* = 0.017), the absolute count of HLA-DR+ CD8br cells (OR = 0.936, 95% CI: 0.882-0.994, P = 0.032), CD19 expression on CD24+ CD27+ B cells (OR = 0.881, 95% CI: 0.808–0.961, P = 0.004), and CD27 expression on CD20− B cells (OR = 0.877, 95% CI: 0.771-0.996, P = 0.043) were all negatively associated with AD risk. Increased side scatter area (SSC-A) of natural killer (NK) cells was also associated with reduced AD risk (OR = 0.913, 95% CI: 0.839-0.993, *P* = 0.034).

Analysis of 1,400 blood metabolites and metabolite ratios identified 18 metabolic traits associated with AD (**Figure 1**). Of these, 11 metabolites or metabolite ratios were associated with increased risk (OR > 1), whereas 7 showed protective associations (OR < 1). Among the risk-associated metabolites, higher levels of 4-acetylphenol sulfate were associated with increased AD risk (OR = 1.156, 95% CI: 1.018-1.312, *P* = 0.025), as were higher levels of 1-oleoyl-GPC (18:1) (OR = 1.222, 95% CI: 1.027-1.454, *P* = 0.024). Protective metabolic features included aconitate (cis or trans) (OR = 0.869, 95% CI: 0.759-0.994, *P* = 0.040), tetradecanedioate (OR = 0.910, 95% CI: 0.839-0.987, *P* = 0.023), and glycolithocholate sulfate (OR = 0.934, 95% CI: 0.876-0.996, *P* = 0.036).

### Identification of genes shared by SMR and MR analyses

We first integrated eQTL data with outcome GWAS data using SMR, and the genome-wide distribution of associations between gene expression and disease risk was visualized in a Manhattan plot (**Figure 2**). This analysis identified 677 genes with potential causal effects. We then performed conventional two-sample MR, selected independent SNPs significantly associated with the disease outcome, and mapped them to 2,034 protein-coding genes. Intersecting the results of these two approaches yielded 61 genes supported by both SMR and MR analyses (**Figure 2**).

### Differential expression validation and final identification of core targets

To determine whether these 61 candidate genes also showed disease-related transcriptional changes, we analyzed an independent transcriptomic dataset containing AD and control samples. Among the 61 genes, 31 were significantly differentially expressed between the two groups. These 31 genes were defined as the core targets in this study because they were supported by three lines of evidence: (1) SMR suggested a potential causal association between altered gene expression and disease risk; (2) conventional MR confirmed a robust effect of genetic variation on the outcome; and (3) differential expression was observed in an independent disease dataset.

Heatmap-based clustering showed clear separation between AD and control samples, indicating that these differentially expressed genes discriminated well between disease states. Notable upregulated genes included *PPP1R16B*, *CCDC69*, *SLC6A12*, and *ANGPT2*, whereas significantly downregulated genes included *KPNA3*, *GIT2*, and *ASCC3* (**Figure 2**). Box plots of individual genes further supported these differences (**Figure 2**).

Chromosomal mapping showed that the target genes were broadly distributed across the autosomes, with several genes clustering on specific chromosomes (**Figure 2**). For example, *CCDC69*, *COMMD10*, and *ARSK* were located on chromosome 5; *SLC6A12*, *TMEM117*, *GIT2*, and *TMEM132D* on chromosome 2; and *SLC24A3*, *ACTR5*, *PPP1R16B*, and *FAM83D* on chromosome 6. To further explore their regulatory relationships, we performed gene co-expression network analysis (**Figure 2**). Most differentially expressed genes were positively correlated, suggesting coordinated transcriptional regulation in AD. The modular structure of the network further implied that these genes may participate in shared biological pathways or regulatory programs.

### Immune cell infiltration analysis

**Figure 3:**
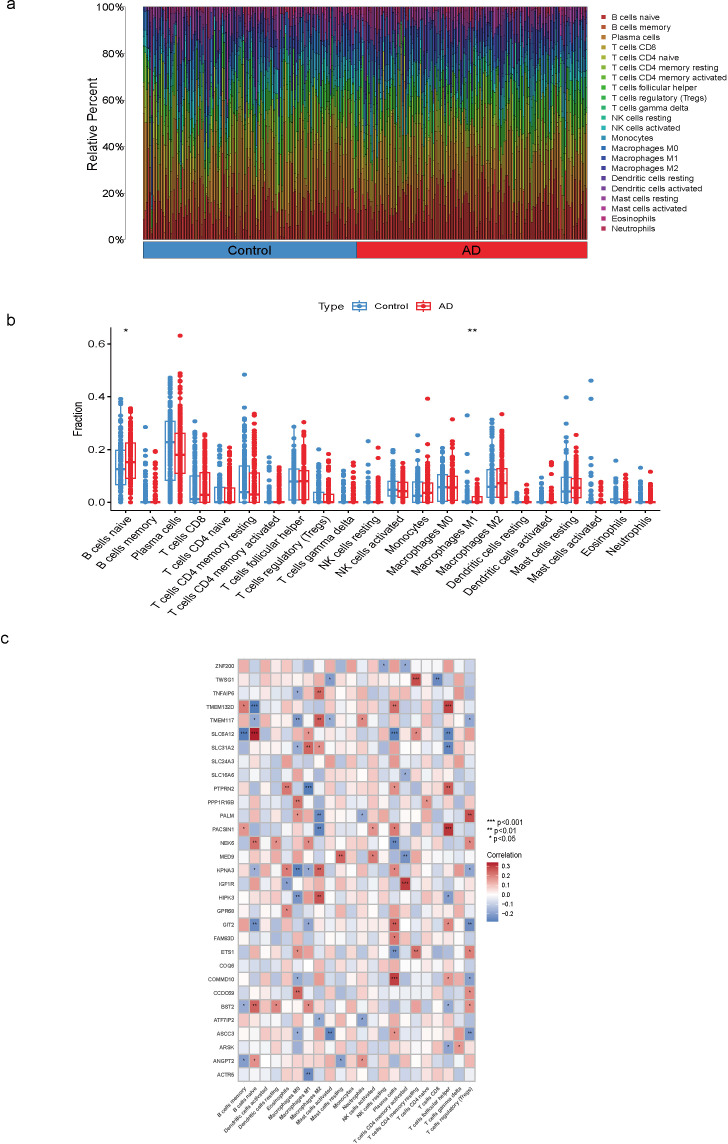
Immune cell infiltration analysis in AD samples. **(a)** Relative abundances of 22 immune cell types in control and AD groups as estimated by the CIBERSORT algorithm. **(b)** Box plots showing immune cell subsets with significant differences in infiltration between the two groups, including M1 macrophages and naive B cells. **(c)** Spearman correlation heatmap showing associations between differentially expressed genes and the abundances of significantly altered immune cell subsets. Immune cell fractions were inferred using the CIBERSORT algorithm based on the LM22 reference matrix. These results represent an estimation of the immune microenvironment and should be interpreted with caution given the tissue-specific differences between brain and peripheral blood. *P < 0.05, **P < 0.01, ***P < 0.001; statistical significance was assessed using the Wilcoxon rank-sum test or independent-samples t test.

To better characterize the immune microenvironment in AD, we estimated the relative abundance of 22 immune cell types in the training set using CIBERSORT. The analysis showed clear alterations in immune cell composition in AD samples, with dysregulation affecting multiple innate and adaptive immune cell populations (**Figure 3**). Compared with controls, changes in innate immune cells were particularly prominent in AD brain tissue. The infiltration proportion of M1 macrophages was significantly increased in the AD group (*P* < 0.01) (**Figure 3**). In addition, the proportion of naive B cells was decreased in the AD group (*P* < 0.05).

We next examined the relationship between gene expression and the altered immune microenvironment by performing Spearman correlation analysis between differentially expressed genes and the immune cell subsets that differed significantly between groups (**Figure 3**). The results showed a complex correlation network. Specifically, *ASCC3* expression was significantly positively correlated with resting plasma cells (*P* < 0.001) but negatively correlated with activated mast cells and regulatory T cells (Tregs). *IGF1R* expression was positively correlated with activated memory CD4 T cells (*P* < 0.01) and negatively correlated with eosinophils (*P* < 0.05). *TWSG1* expression was negatively correlated with activated mast cells and positively correlated with resting memory CD4 T cells. These findings suggest that the target genes may contribute to AD-related neuroinflammation by influencing the infiltration and functional state of specific immune cell subsets.

### Construction and validation of the predictive model

**Figure 4:**
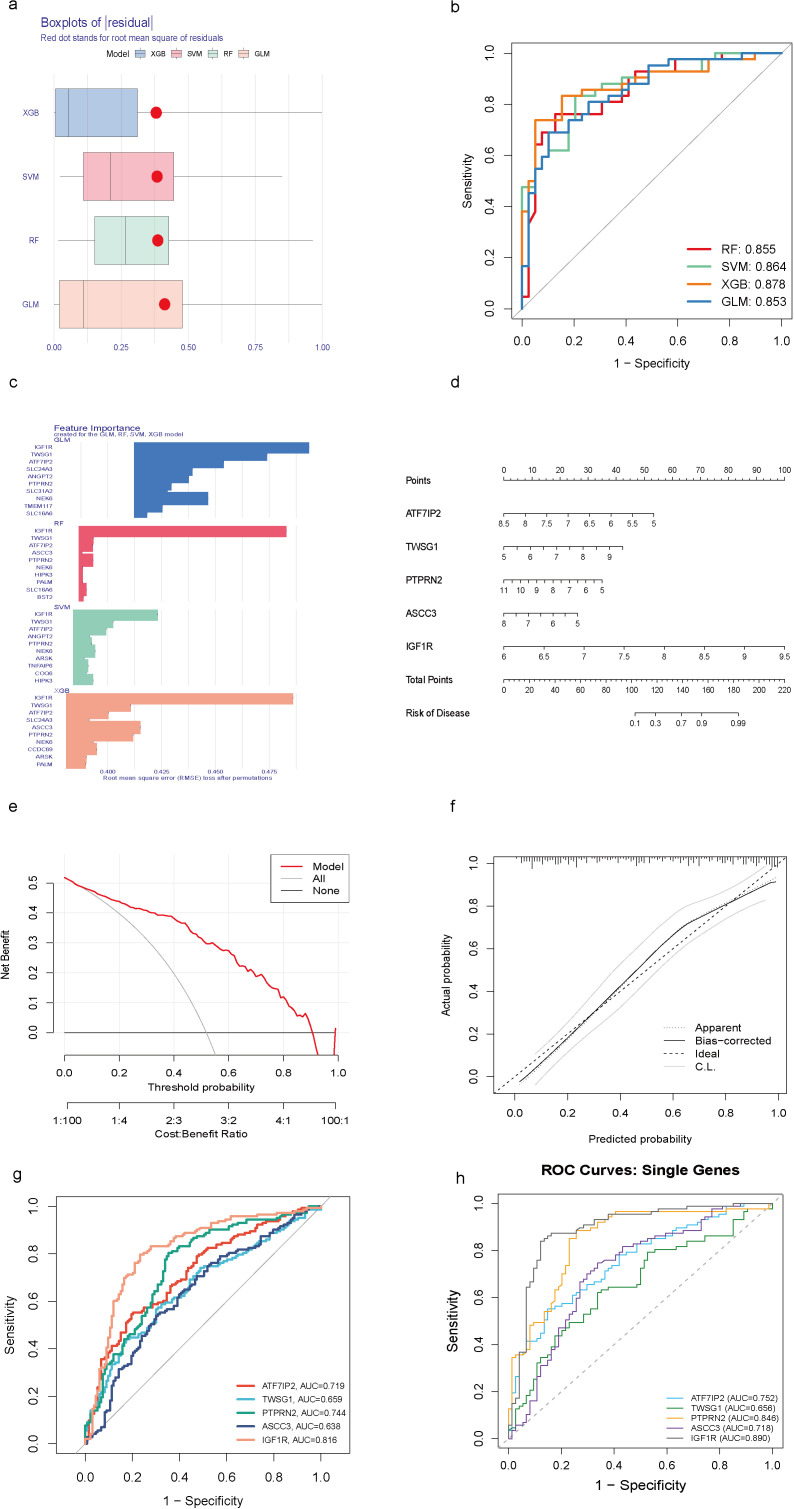
Construction and validation of a machine learning-based AD risk prediction model. **(a)** Comparison of residual box plots for four algorithms: random forest, support vector machine, generalized linear model, and XGBoost. **(b)** ROC curves showing that the XGBoost model achieved the best performance (AUC = 0.878). **(c)** Feature importance ranking identifying five key genes (ATF7IP2, TWSG1, PTPRN2, ASCC3, and IGF1R). **(d)** Nomogram for AD risk prediction based on the five-gene signature. **(e)** Decision curve analysis showing that the model provided a positive net benefit across a threshold probability range of 0.5-0.9. **(f)** Calibration curve showing agreement between predicted and observed probabilities. **(g)** AUC values of individual genes in the training set. **(h)** AUC values of individual genes in the external validation set.

Based on the differential expression results, we used four machine learning algorithms-random forest, support vector machine, generalized linear model, and extreme gradient boosting (XGBoost)-to build AD risk prediction models. Performance was assessed using ten-fold cross-validation, which showed that XGBoost had the best overall performance. Residual analysis indicated that the XGBoost model had the most concentrated residual distribution and the lowest root mean square error (**Figure 4**). Receiver operating characteristic (ROC) analysis confirmed its superior performance, with an AUC of 0.878 (**Figure 4**). Using the built-in feature importance ranking of XGBoost, we identified the five genes that contributed most strongly to the model: *ATF7IP2*, *TWSG1*, *PTPRN2*, *ASCC3* and *IGF1R* (**Figure 4**). All five genes were differentially expressed between groups, highlighting their value as potential AD biomarkers. Based on these genes, we constructed a nomogram for individualized prediction of AD risk (**Figure 4**). In the nomogram, each gene expression level was assigned a score, and the total score was converted into a predicted probability of AD. Decision curve analysis showed that the model provided a positive net benefit across a threshold probability range of 0.5 to 0.9, outperforming strategies of treating all patients or treating none (**Figure 4**). The calibration curve showed good agreement between predicted and observed probabilities (**Figure 4**). The predictive value of the five feature genes was further evaluated in the independent external validation dataset GSE36980. ROC analysis of the individual genes showed that *IGF1R* had the best discriminative ability (AUC = 0.890), followed by *PTPRN2* (AUC = 0.846), *ATF7IP2* (AUC = 0.752), while *ASCC3* (AUC = 0.718), and *TWSG1* (AUC = 0.656) exhibited modest diagnostic performance (**Figure 4**). Together, these exploratory findings suggest that the individual genes from the model hold potential clinical relevance, though its definitive diagnostic accuracy must be rigorously evaluated in future large-scale, independent clinical cohorts.

### Single-cell RNA sequencing reveals cell type-specific expression of key genes

**Figure 5:**
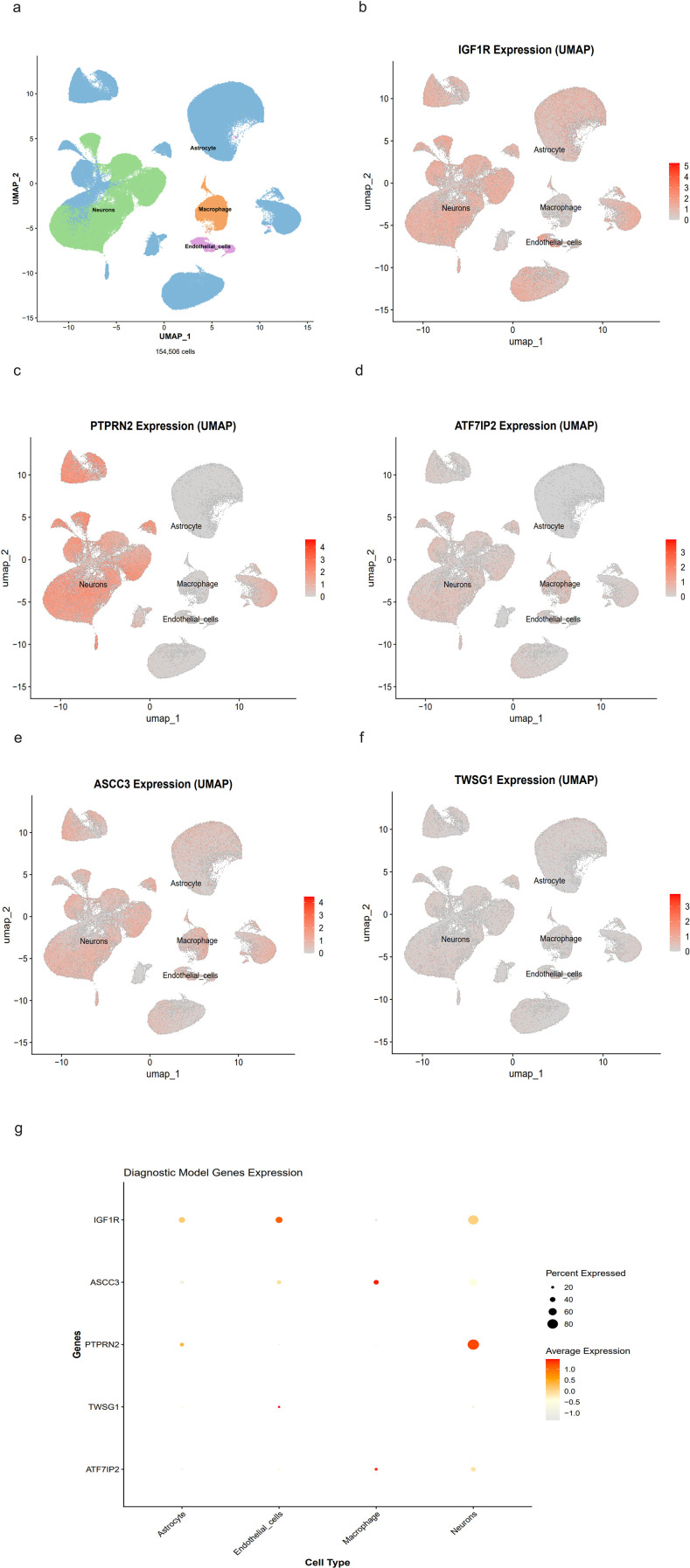
Single-cell RNA sequencing reveals cell type-specific expression of key genes in brain tissue. **(a)** UMAP plot showing the four major cell types identified in the GSE157827 dataset: astrocytes, neurons, macrophages, and endothelial cells. **(b–f)** UMAP feature plots showing expression of the five genes across individual cells. **(g)** Bubble plot summarizing the proportion of expressing cells and the average expression levels of the five key genes (ATF7IP2, TWSG1, PTPRN2, ASCC3 and IGF1R) across cell types.

To define the cellular origins of the five key genes (*ATF7IP2*, *TWSG1*, *PTPRN2*, *ASCC3*, and *IGF1R*) in brain tissue and to characterize their expression under AD pathology, we analyzed the GSE157827 dataset. After quality control, normalization, and clustering, four major cell types were identified: astrocytes, neurons, macrophages, and endothelial cells (**Figure 5**). Notably, standard scRNA-seq protocols often struggle to capture myelin-heavy oligodendrocytes, which explains their absence in this dataset. Consequently, our subsequent spatial transcriptomic analysis serves as the primary basis for exploring oligodendrocyte-specific patterns, although these findings should be interpreted with caution as they represent spatial associations rather than direct functional evidence. Cell type-specific analysis showed distinct expression patterns for the five genes across these populations (**Figure 5**). *ATF7IP2* was expressed mainly in macrophages and showed low expression in astrocytes (**Figure 5**). *TWSG1* was highly expressed almost exclusively in endothelial cells. *PTPRN2* was highly expressed in neurons and astrocytes, with relatively low expression in the other cell types. *ASCC3* showed high expression in macrophages and endothelial cells. *IGF1R* was highly expressed in astrocytes, neurons, and endothelial cells but showed low expression in macrophages.

### Spatial transcriptomics delineates the spatial distribution of key genes across brain regions

**Figure 6:**
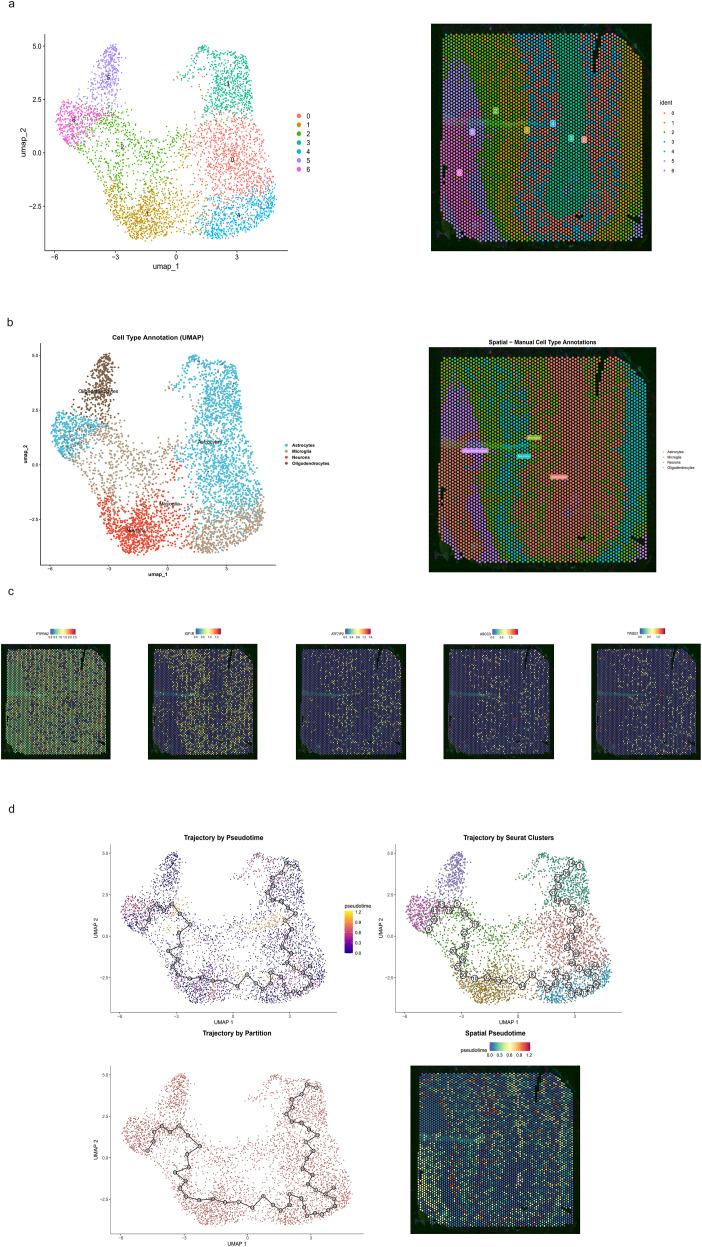
Spatial transcriptomic analysis reveals the spatial distribution of key genes in brain tissue. **(a)** UMAP plot showing seven clusters identified in the GSE220442 dataset. **(b)** UMAP plot showing cell type annotations in the GSE220442 dataset, including astrocytes, microglia, neurons, and oligodendrocytes. **(c)** Heatmap showing the spatial expression patterns of the five key genes in the spatial transcriptomic dataset. **(d)** Pseudotime analysis.

To visualize the spatial expression patterns of the key genes *in situ*, we analyzed the spatial transcriptomic dataset from the human middle temporal gyrus (GSE220442). Clustering analysis partitioned the tissue sections into distinct regions annotated as astrocytes, microglia, neurons, and oligodendrocytes (**Figure 6**). We then extracted the expression levels of the five core genes across these cell types and compared their spatial patterns.

The results suggested that the five genes could be broadly divided into two groups based on their expression profiles, reflecting clearly different cell type-specific distributions. *PTPRN2* showed broad and relatively uniform expression across all four cell types, without obvious enrichment in any one population (**Figure 6**).

In contrast, spatial transcriptomic profiling suggested that *IGF1R*, *ASCC3* and *TWSG1* displayed highly concordant cell type-specific patterns. Based primarily on this spatial distribution, these three genes appeared prominently associated with oligodendrocytes (**Table 1**), suggesting a potential, though still provisional, cell-specific regulatory module. Compared with the other three cell types, the mean expression levels of *IGF1R* (log_2_FC=−2.119, *P* < 0.001) and *TWSG1* (log_2_FC = −1.971, *P* < 0.001) were significantly lower in oligodendrocytes, whereas *ASCC3* expression was significantly higher in oligodendrocytes (log_2_FC=3.591, *P* < 0.001). *ATF7IP2* showed opposite patterns in two cell types, with decreased expression in oligodendrocytes (log_2_FC = −3.308, *P* < 0.001) but increased expression in microglia (log_2_FC = 1.031, *P* < 0.001). Pseudotime analysis further suggested that oligodendrocytes were enriched in early states, whereas microglia were enriched in intermediate states (**Figure 6**).

**Table 1:** Differentially expressed genes identified by spatial transcriptomics.

Genes	Cluster (cell type)	Log_2_ FC	P value
ASCC3	5(Oligodendrocytes)	3.591	<0.001
ATF7IP2	4(Microglia)	1.031	<0.001
ATF7IP2	5(Oligodendrocytes)	-3.308	<0.001
IGF1R	5(Oligodendrocytes)	-2.119	<0.001
TWSG1	5(Oligodendrocytes)	-1.971	<0.001

Genes with significant differential expression across clusters (cell types) in human middle temporal gyrus samples (GSE220442). Log_2_ FC and *P*-values are shown. Cluster 4: microglia; Cluster 5: oligodendrocytes. Positive Log_2_ FC indicates upregulation in AD compared to control; negative Log_2_ FC indicates downregulation. Statistical significance was determined by Wilcoxon rank-sum test with Benjamini-Hochberg correction.

### MR and qPCR trend confirmation in pooled PBMC samples

**Figure 7:**
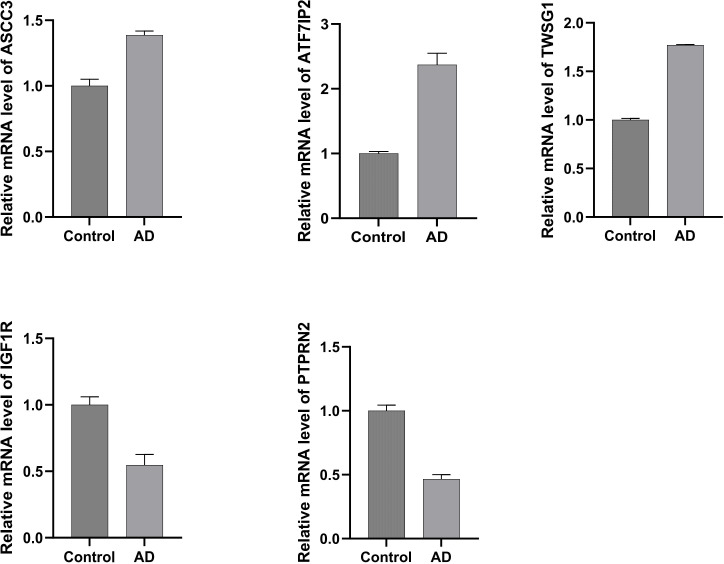
Relative expression levels of five genes in pooled PBMCs from patients with AD and healthy controls as measured by qPCR. qPCR analysis showed that ATF7IP2, TWSG1, and ASCC3 exhibited an upward trend in the AD group, whereas IGF1R and PTPRN2 showed a downward trend, consistent with the potential causal directions inferred from eQTL-based MR analysis. (n=1 pooled sample per group; error bars represent SD across three technical replicates.).

We next performed two-sample MR analyses using eQTLs for the target genes and AD GWAS data. Among the five genes, *ASCC3* (OR = 1.198, 95% CI: 1.004-1.429), *ATF7IP2* (OR = 1.200, 95% CI: 1.057-1.363), and *TWSG1* (OR = 1.357, 95% CI: 1.085-1.698) were identified as risk genes, with genetically predicted higher expression associated with increased AD risk. In contrast, *IGF1R* (OR = 0.859, 95% CI: 0.739-0.998) and *PTPRN2* (OR = 0.914, 95% CI: 0.863-0.969) were identified as protective genes, for which higher expression was associated with reduced AD risk (**Table 3**). To confirm the expression trends of the key genes identified by the multi-omics analyses in an independent peripheral blood cohort, we measured the mRNA expression levels of the five feature genes in pooled PBMCs derived from 5 patients with AD and 5 age- and sex-matched healthy controls using qPCR (**Figure 7**). The qPCR trend confirmation showed that the direction of expression change was fully consistent with the potential causal directions inferred from eQTL-based MR analysis (**Table 2**). Specifically, mRNA levels of *ATF7IP2* (mean 2.37 vs. 1.00), *TWSG1* (1.77 vs. 1.00), and *ASCC3* (1.39 vs. 1.00) showed an upward trend in PBMCs from patients with AD compared to controls. Conversely, expression levels of *IGF1R* (0.55 vs. 1.00) and *PTPRN2* (0.47 vs. 1.00) showed a downward trend in the AD group. These qPCR results reflect group-level expression trends from pooled RNA rather than biological variability across individual participants.

**Table 2:** qPCR trend confirmation of key gene expression in pooled PBMCs.

Group	Genes	Technical replicate 1	Technical replicate 2	Technical replicate 3	Mean± SD
Control	ASCC3	1.06	0.97	0.97	1.00 ± 0.04
AD	ASCC3	1.42	1.37	1.37	1.39 ± 0.02
Control	ATF7IP2	1.04	0.97	0.99	1.00 ± 0.03
AD	ATF7IP2	2.19	2.54	2.38	2.37 ± 0.14
Control	IGF1R	1.04	1.03	0.93	1.00 ± 0.05
AD	IGF1R	0.59	0.59	0.45	0.55 ± 0.07
Control	PTPRN2	1.05	0.99	0.97	1.00 ± 0.04
AD	PTPRN2	0.49	0.43	0.49	0.47 ± 0.03
Control	TWSG1	1.01	0.98	1.01	1.00 ± 0.01
AD	TWSG1	1.77	1.78	1.77	1.77 ± 0.01

a pooled AD sample (derived from n=5 patients) and a pooled control sample (derived from n=5 healthy controls). Each gene was measured in three technical replicates per sample (n=1 pooled sample per group; error bars represent SD across three technical replicates). Mean expression values ± standard deviations (SD) are shown. Statistical significance testing was not performed due to the pooled nature of the biological samples.

**Table 3:** Mendelian randomization results for eQTLs of target genes with AD risk.

Exposure	Outcome	Method	P value	OR	95% CI
ASCC3	AD	Inverse variance weighted	0.045	1.198	1.004, 1.429
ATF7IP2	AD	Inverse variance weighted	0.005	1.200	1.057, 1.363
IGF1R	AD	Inverse variance weighted	0.047	0.859	0.739,0.998
PTPRN2	AD	Inverse variance weighted	0.002	0.914	0.863, 0.969
TWSG1	AD	Inverse variance weighted	0.008	1.357	1.085, 1.698

The potential causal estimates were primarily calculated using the Inverse variance weighted (IVW) method. All instrumental variables satisfied *F*>10. Sensitivity analyses (MR-Egger, weighted median, MR-PRESSO) showed no significant horizontal pleiotropy. OR, odds ratio; CI, confidence interval.

### Transcriptomic expression trends of target genes in the validation cohort

**Figure 8:**
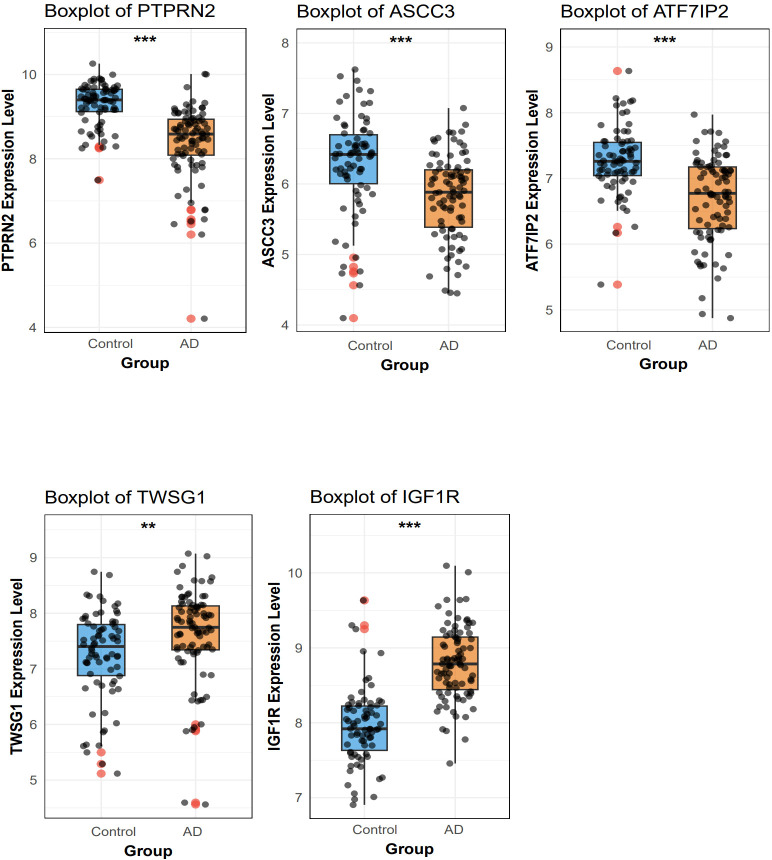
Box plots showing expression trends of the five key genes in the validation cohort. The figure shows the expression patterns of ATF7IP2, TWSG1, PTPRN2, ASCC3, and IGF1R in the AD and control groups in the validation cohort, reported as consistent with the results obtained in the training set. ** P < 0.01, *** P < 0.001.

Due to the complex biological nature of peripheral-central interactions, we observed distinct expression patterns across different datasets. A comprehensive summary of the expression direction for each key gene across the training set, external validation cohort, MR inference, and qPCR trend confirmation is provided in **Supplementary Table 6**. In the validation cohort, transcriptomic analysis showed that *PTPRN2*, *ASCC3*, and *ATF7IP2* were significantly downregulated in AD, whereas *TWSG1* and *IGF1R* were significantly upregulated (**Figure 8**). These trends were reported as consistent with those observed in the training set.

## Discussion

Using a systematic multi-omics strategy, this study combined genetic potential causal inference, transcriptomic differential expression analysis, machine learning, single-cell and spatial transcriptomics, and experimental confirmation in an independent clinical cohort. This framework allowed us to define a complex regulatory network underlying the AD gut microbiota-blood metabolite-immune cell-brain axis. Our results identified a set of high-confidence core genes and revealed dynamic peripheral-central interactions as well as cell type-specific patterns of gene regulation. Together, these findings offer a broader view of AD as a systemic disorder rather than a disease confined to the brain.

The MR analyses provided genetic evidence that gut microbiota, blood metabolites, and immune cell phenotypes are each associated with AD, showing patterns consistent with potential causal effects. Among the gut microbes, a higher relative abundance of P. clara was associated with increased AD risk, whereas B. xylanisolvens appeared protective. Previous studies have shown that wheat germ polysaccharide can markedly alter gut microbial composition and substantially increase the abundance of Bacteroides, particularly B. xylanisolvens (19). This shift is accompanied by increased production of short-chain fatty acids and indole-3-lactic acid, metabolites that help maintain intestinal barrier integrity and exert anti-inflammatory and neuromodulatory effects. The protective role of B. xylanisolvens in AD may therefore be mediated through the production of beneficial metabolites. By contrast, prior work suggests that P. clara may alter peripheral immune function, especially monocyte function, by influencing bile acid metabolism. This raises the possibility that P. clara contributes to AD risk through immune dysregulation (20).

Our MR analysis also identified several immune phenotypes associated with AD, particularly involving B cells, T cells, and NK cells. The CD28 signaling pathway was especially notable. CD28 is a key co-stimulatory receptor on T cells, and when it binds B7 ligands (CD80/CD86) on antigen-presenting cells, it provides the second signal required for T-cell activation (21). We found that increased CD28 expression on CD45RA+ CD4+ T cells was associated with increased AD risk, whereas higher proportions of CD28+ DN (CD4-CD8-) T cells and CD28+ CD45RA+ CD8^br T cells among CD8br cells were associated with lower risk. Previous studies have shown that expansion of CD28-CD45RA+ CD8+ T cells, also known as terminally differentiated effector memory T cells re-expressing CD45RA (TEMRA), is linked to immunosenescence and chronic inflammation, which fits current models of AD pathogenesis (22, 23). CD28+ DN regulatory T cells have also been implicated as mediators of microbiota-related protection in other neuroimmune disorders. This suggests that CD28+ DN T cells may act as immunoregulatory intermediates through which gut microbes influence distant neuroinflammatory responses (24). Likewise, CD28+ CD45RA+ CD8br T cells are generally regarded as a marker of naive or early memory T-cell status, and preserving this population may help maintain immune surveillance and limit disease progression (25).

The metabolite analysis identified 18 blood metabolic traits associated with AD, involving amino acid, lipid, and energy metabolism. Notably, the Spermidine to pyruvate ratio exhibited the strongest protective association with AD risk (OR = 0.827, showing the largest protective effect size among all metabolic features). Spermidine is a natural polyamine increasingly recognized for its ability to preserve cognitive function by inducing autophagy, suppressing neuroinflammation, and protecting against oxidative stress (26, 27). The association between this ratio and reduced AD risk suggests that the synergy between polyamine homeostasis and efficient energy metabolism (represented by pyruvate) may be a critical systemic defense mechanism. Furthermore, previous studies have shown that gut microbiota-derived metabolites can influence systemic spermidine levels, suggesting that this ratio may act as a key protective bridge within the gut-brain axis (28, 29). One notable finding was that a higher 3-methyl-2-oxovalerate to 4-methyl-2-oxopentanoate ratio was associated with increased AD risk, suggesting disrupted branched-chain amino acid (BCAA) metabolism. Previous studies have shown that environmental toxicants such as phthalates can disturb gut microbial composition and alter microbially derived metabolites, including BCAAs, leading to lipid metabolic dysfunction and neurotoxicity (30). BCAA dysregulation may therefore represent a shared mechanism that promotes oxidative stress, inflammation, mitochondrial dysfunction, and impaired autophagy in AD (31, 32).

By intersecting the SMR and conventional MR results, we identified 61 genetically regulated genes associated with AD risk, 31 of which were differentially expressed in AD brain tissue. Machine learning then selected a five-gene signature-*ATF7IP2*, *TWSG1*, *PTPRN2*, *ASCC3* and *IGF1R*-that showed strong predictive performance across multiple datasets. In independent PBMC samples, qPCR confirmed expression trends that matched the potential causal directions inferred from eQTL-based MR. Notably, some expression trends in bulk brain transcriptomic data differed from those observed in blood. This difference is more likely to reflect tissue-specific and cell type-specific regulation, as well as dynamic changes across disease stages, than a technical artifact.

*ATF7IP2* was first identified for its specific role in male germ cell meiosis, where it regulates histone H3 lysine 9 trimethylation (H3K9me3) and is essential for spermatogenesis (33). More recent GWAS and whole-exome sequencing studies have implicated *ATF7IP2* in a broader range of conditions, including recurrent visceral leishmaniasis, common variable immunodeficiency, endometrial cancer, and Lewy body disease. These observations suggest that *ATF7IP2* has functions beyond reproduction and may be involved in immune regulation, tumor biology, and neurodegeneration (34–37). In our study, qPCR showed an upward trend for *ATF7IP2* expression in PBMCs from patients with AD, fully consistent with the eQTL-based MR finding that higher expression increases AD risk. In contrast, *ATF7IP2* was downregulated in bulk brain transcriptomic data, whereas spatial transcriptomics showed significant upregulation in microglia and significant downregulation in oligodendrocytes. Because microglia shift from a homeostatic to an activated state in response to Aβ and tau pathology, the concordant upregulation of *ATF7IP2* in both microglia and PBMCs suggests that it may be part of a myeloid immune activation program in AD (38, 39).

*IGF1R* encodes a transmembrane tyrosine kinase receptor that is central to cell growth, metabolism, survival, and differentiation (40). Our qPCR data showed a downward trend for IGF1R expression in PBMCs from patients with AD, which was fully consistent with the eQTL-based MR inference that lower expression increases AD risk. However, *IGF1R* showed mild upregulation in bulk brain transcriptomic data, whereas spatial transcriptomics demonstrated marked downregulation in oligodendrocytes. A large body of evidence supports a close link between type 2 diabetes and AD and suggests that brain insulin resistance is a major pathological connection between the two disorders (41). Functional abnormalities in insulin signaling molecules such as IRS1, Akt, and GSK3β have been reported in the brains of patients with both type 2 diabetes and AD. Impaired insulin signaling can promote mitochondrial dysfunction, oxidative stress, abnormal protein aggregation, and cognitive decline (42). Our findings add molecular support to this framework by suggesting that reduced *IGF1R* expression in oligodendrocytes may contribute to brain insulin resistance. Single-cell data further showed that *IGF1R* is highly expressed in astrocytes and endothelial cells and is upregulated in AD, which may reflect compensatory responses such as reactive gliosis or attempts to maintain blood–brain barrier integrity. The mild increase seen at the bulk tissue level may therefore reflect opposing changes across cell types (42). In peripheral blood, reduced *IGF1R* expression may indicate broader systemic impairment of insulin signaling.

Spatial transcriptomic analysis further showed that *IGF1R*, *ASCC3* and *TWSG1* are strongly co-localized in oligodendrocytes in AD brain tissue, forming a potential oligodendrocyte response module. This is important because it challenges the traditional view of oligodendrocytes as passive bystanders in AD and instead suggests that they may play an active role in disease progression. Oligodendrocytes do more than produce myelin; they also support neuronal metabolism and survival. *IGF1R* mediates the trophic and pro-survival effects of IGF-1, so its downregulation may reduce oligodendrocyte responsiveness and impair myelination and cell survival. *ASCC3* is involved in DNA damage repair, and because oligodendrocytes are highly vulnerable to oxidative damage, its upregulation may represent a protective response (43). *TWSG1*, an extracellular regulator of bone morphogenetic protein (BMP) signaling, has been implicated in oligodendrocyte precursor cell differentiation. Extensive *in vitro* and *in vivo* work indicates that BMP signaling inhibits differentiation into mature oligodendrocytes while promoting an astrocytic fate (44, 45). Reduced *TWSG1* expression may therefore alter oligodendrocyte precursor dynamics and contribute to disease progression. We hypothesize that these three genes *IGF1R*, *ASCC3*, *TWSG1*) may form a dynamic regulatory system that shapes the oligodendrocyte response to AD pathology. However, because this interpretation relies heavily on the spatial co-localization evidence provided here, it remains speculative. Future studies employing cell-specific knockouts or functional assays are required to move beyond this provisional working hypothesis.

Despite the strengths of this integrative design and the use of multiple independent datasets, several limitations should be acknowledged. First, we employed an exploratory threshold (*P* < 0.05) for our initial high-throughput MR screening to preserve potential biological signals; while downstream multi-omics integration was used for rigorous validation, these initial MR findings should be interpreted as hypothesis-generating. Second, our immune infiltration analysis in brain tissue utilized CIBERSORT’s LM22 matrix, which is derived from peripheral blood. This introduces inherent limitations in distinguishing brain-resident microglia from infiltrating peripheral macrophages; therefore, these results represent an estimation of the broader immune microenvironment rather than definitive local cell counts.

Furthermore, there were observable discrepancies in the expression directions of *ASCC3*, *PTPRN2*, and *ATF7IP2* between the central nervous system transcriptomics and peripheral blood expression. This likely reflects complex, tissue-specific compensatory mechanisms. Consequently, while our diagnostic model performs well in brain tissue, these genes are currently better suited as candidates for mechanistic investigation rather than as ready-to-use blood diagnostic biomarkers. Their direct translation to peripheral blood requires specific retraining and optimization on large-scale blood datasets. Finally, the qPCR trend confirmation utilized a pooled RNA strategy. While this approach effectively verified the overarching population-level expression trends consistent with our MR inferences, the lack of individual biological replicates limits statistical power. Future large-scale, individual-level clinical cohorts are necessary to robustly validate these peripheral biomarkers. Furthermore, while the SMR results strongly support genetic association and target prioritization, the lack of formal colocalization analysis (e.g., the HEIDI test) means that these findings do not by themselves prove a shared causal variant. Future studies incorporating comprehensive colocalization methodologies are warranted.

## Conclusions

Despite these limitations, this study provides several important advances through a systematic multi-omics framework. First, MR analysis offered genetic evidence that gut microbiota, blood metabolites, and immune cell phenotypes are associated with AD (showing patterns consistent with potential causal effects), supporting the view that AD may have important peripheral drivers. Second, by integrating SMR, conventional MR, and machine learning, we identified a five-gene panel consisting of *ATF7IP2*, *TWSG1*, *PTPRN2*, *ASCC3*, and *IGF1R* in the training data and further evaluated the diagnostic potential of the individual feature genes in an external dataset. The agreement between qPCR findings and the directions inferred from eQTL-based MR further strengthens the case for these genes as candidates for future mechanistic and biomarker investigation. Third, single-cell RNA sequencing revealed distinct expression patterns across brain cell types, with *ATF7IP2* upregulated in microglia and *IGF1R* downregulated in oligodendrocytes, offering important clues to their biological roles. Spatial transcriptomics further showed strong co-localization of *IGF1R*, *ASCC3*, and *TWSG1* in oligodendrocytes, leading us to propose an oligodendrocyte response module as a potential new unit of AD pathology. However, given the reliance on spatial transcriptomic evidence, this finding should be viewed as a biologically plausible but provisional working hypothesis that requires further rigorous validation in independent cohorts. Overall, by linking gut microbiota, circulating metabolites, immune cells, and the central nervous system, this study supports a systems-level view of AD as a disorder that extends beyond the brain alone.

## Data Availability

The original contributions presented in the study are included in the article/Supplementary Material. The public transcriptomic data presented in the study are deposited in the GEO repository, accession numbers GSE138260, GSE37263, GSE5281, GSE29378, and GSE36980 (https://www.ncbi.nlm.nih.gov/geo/). The single-cell RNA sequencing data and spatial transcriptomic data presented in the study are deposited in the GEO repository, accession numbers GSE157827 and GSE220442. Genetic association data and eQTL statistics presented in the study are deposited in the IEU Open GWAS database (https://opengwas.io/datasets/).
