# Supplementary material for: A multi-omics framework integrating gut microbiota, blood metabolites, and immune cells to elucidate the pathogenesis of Alzheimer’s disease
Source: Front Immunol. 2026 Jul 6;17:1842398. doi: 10.3389/fimmu.2026.1842398 (PMC13381253; doi:10.3389/fimmu.2026.1842398)
Supplement: Supplementary file 1 [file Image1.pdf]

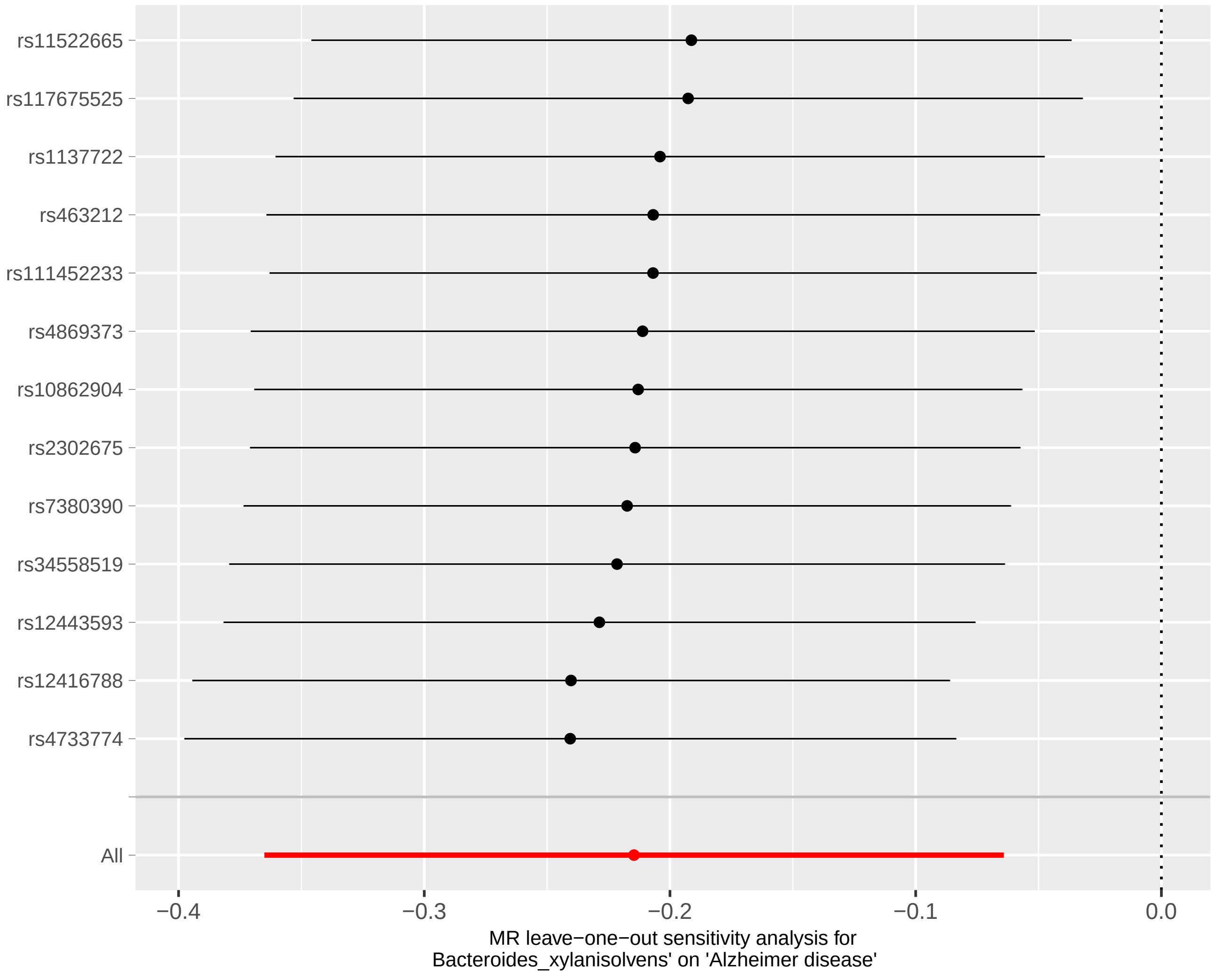
rs11522665
rs117675525
rs1137722
rs463212
rs111452233
rs4869373
rs10862904
rs2302675
rs7380390
rs34558519
rs12443593
rs12416788
rs4733774
All
−0.4
−0.3
−0.2
−0.1
0.0
MR leave−one−out sensitivity analysis for
Bacteroides_xylanisolvens' on 'Alzheimer disease'

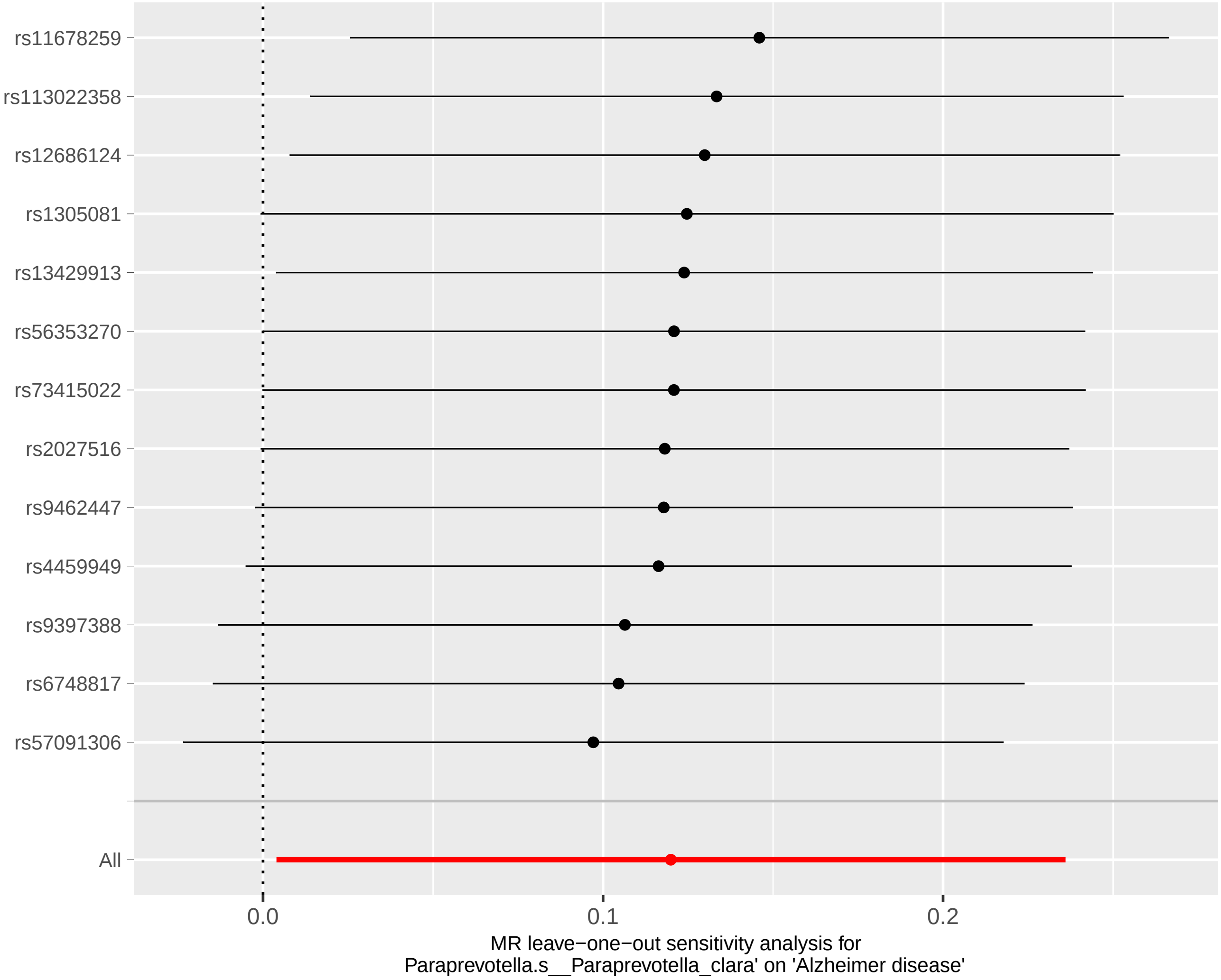
rs11678259
rs113022358
rs12686124
rs1305081
rs13429913
rs56353270
rs73415022
rs2027516
rs9462447
rs4459949
rs9397388
rs6748817
rs57091306
All
0.0
0.1
0.2
MR leave−one−out sensitivity analysis for
Paraprevotella.s__Paraprevotella_clara' on 'Alzheimer disease'

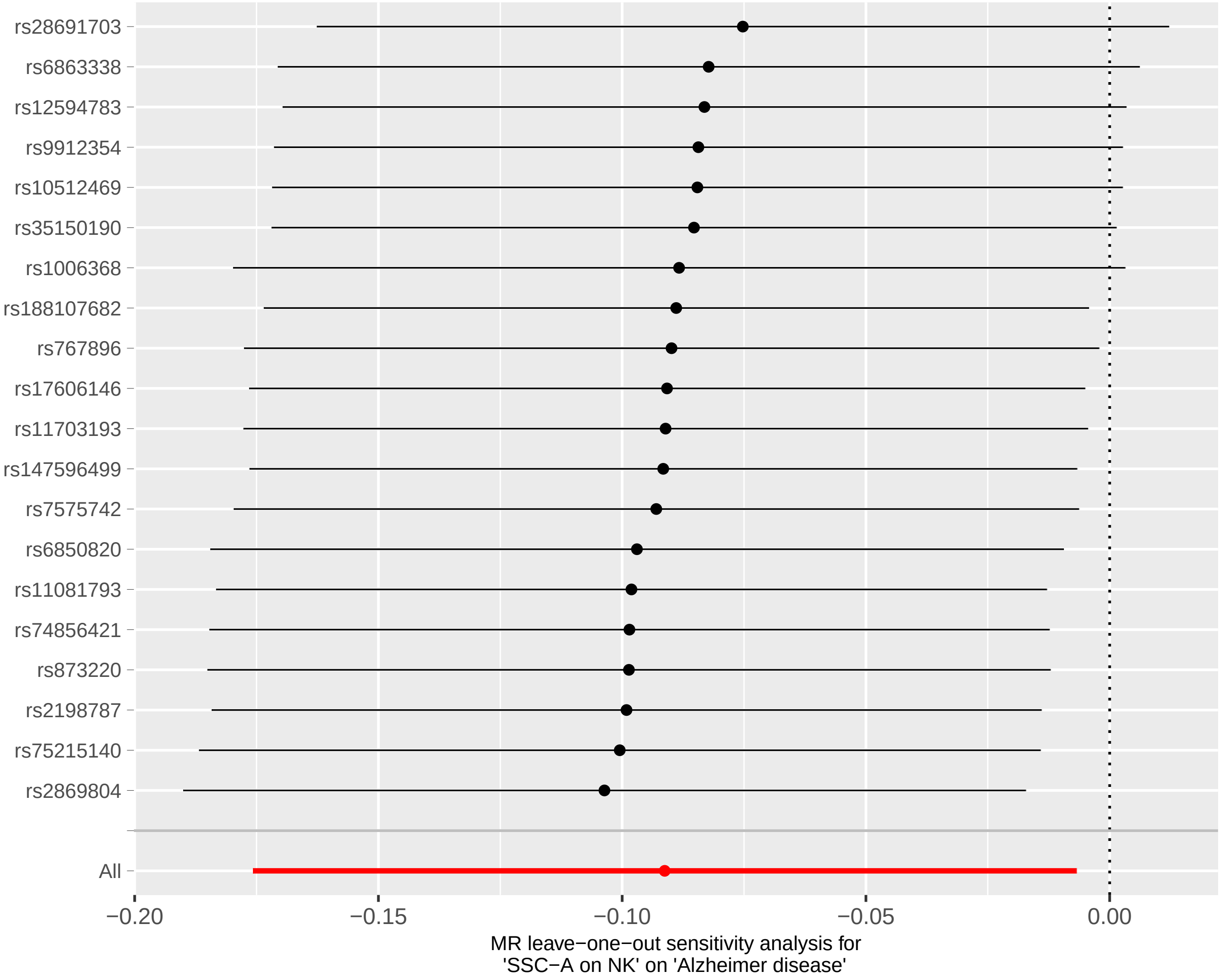
rs28691703
rs6863338
rs12594783
rs9912354
rs10512469
rs35150190
rs1006368
rs188107682
rs767896
rs17606146
rs11703193
rs147596499
rs7575742
rs6850820
rs11081793
rs74856421
rs873220
rs2198787
rs75215140
rs2869804
All
−0.20
−0.15
−0.10
−0.05
0.00
MR leave−one−out sensitivity analysis for
'SSC−A on NK' on 'Alzheimer disease'

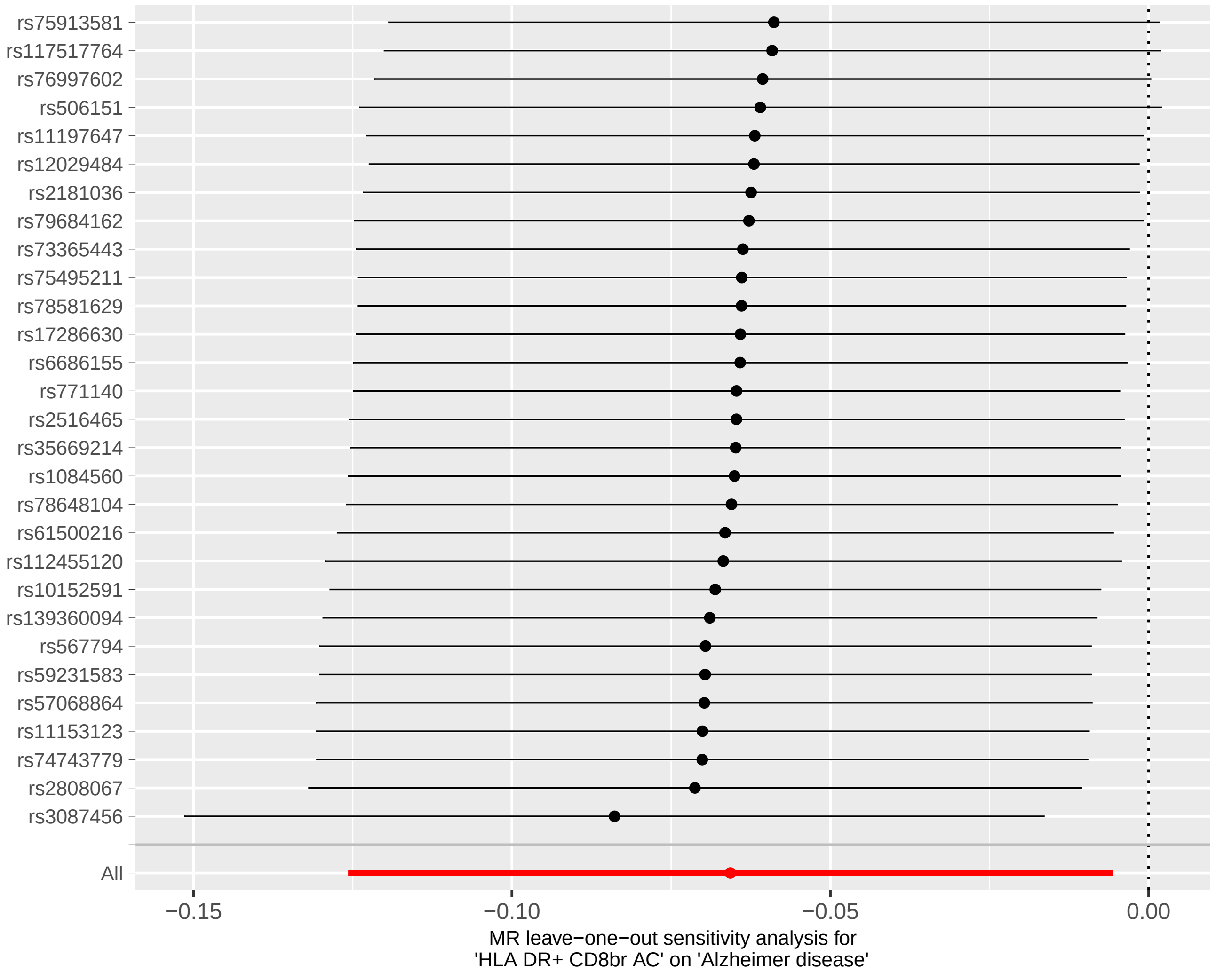
rs75913581
rs117517764
rs76997602
rs506151
rs11197647
rs12029484
rs2181036
rs79684162
rs73365443
rs75495211
rs78581629
rs17286630
rs6686155
rs771140
rs2516465
rs35669214
rs1084560
rs78648104
rs61500216
rs112455120
rs10152591
rs139360094
rs567794
rs59231583
rs57068864
rs11153123
rs74743779
rs2808067
rs3087456
All
−0.15
−0.10
−0.05
0.00
MR leave−one−out sensitivity analysis for
'HLA DR+ CD8br AC' on 'Alzheimer disease'

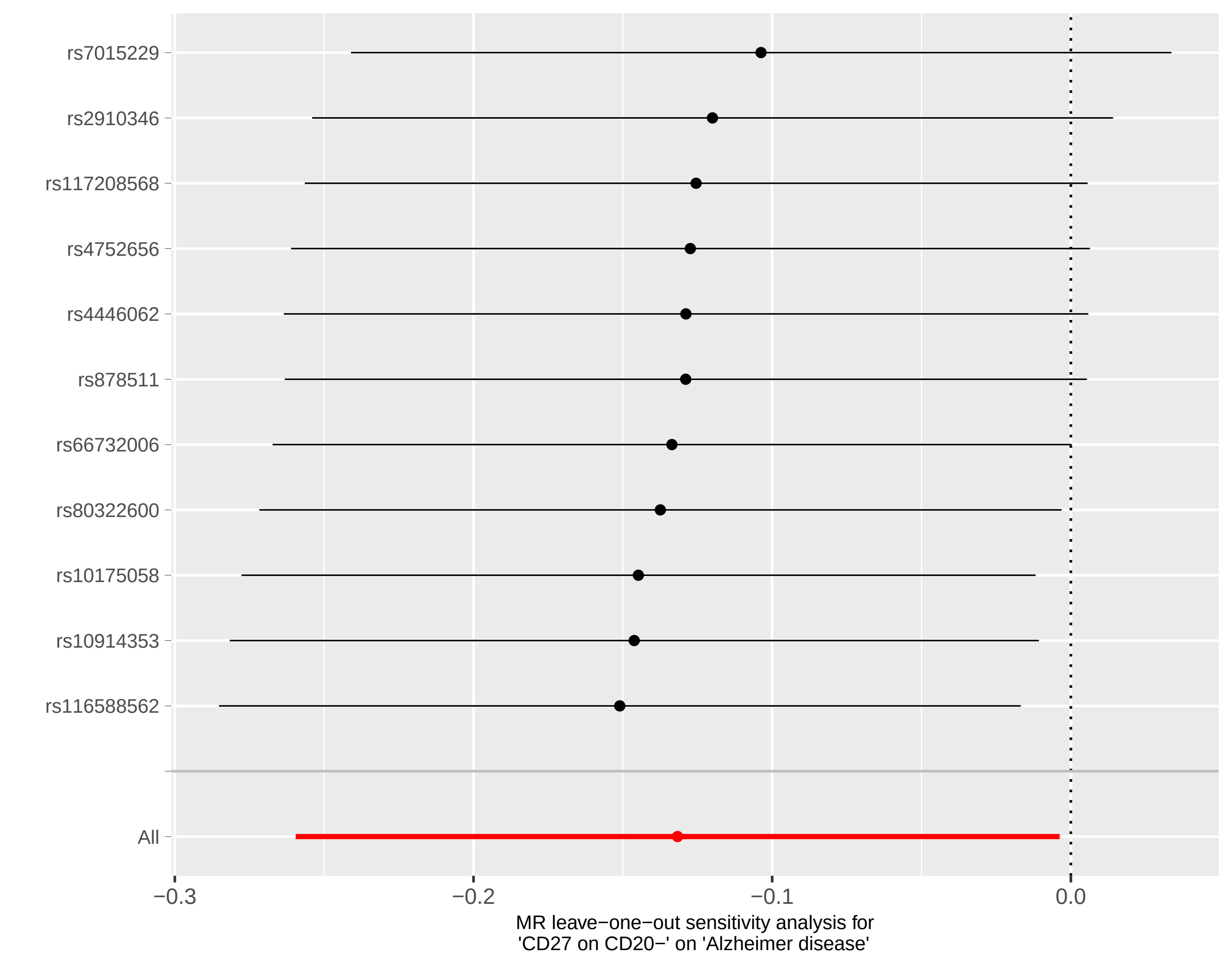
rs7015229
rs2910346
rs117208568
rs4752656
rs4446062
rs878511
rs66732006
rs80322600
rs10175058
rs10914353
rs116588562
All
−0.3
−0.2
−0.1
0.0
MR leave−one−out sensitivity analysis for
'CD27 on CD20−' on 'Alzheimer disease'

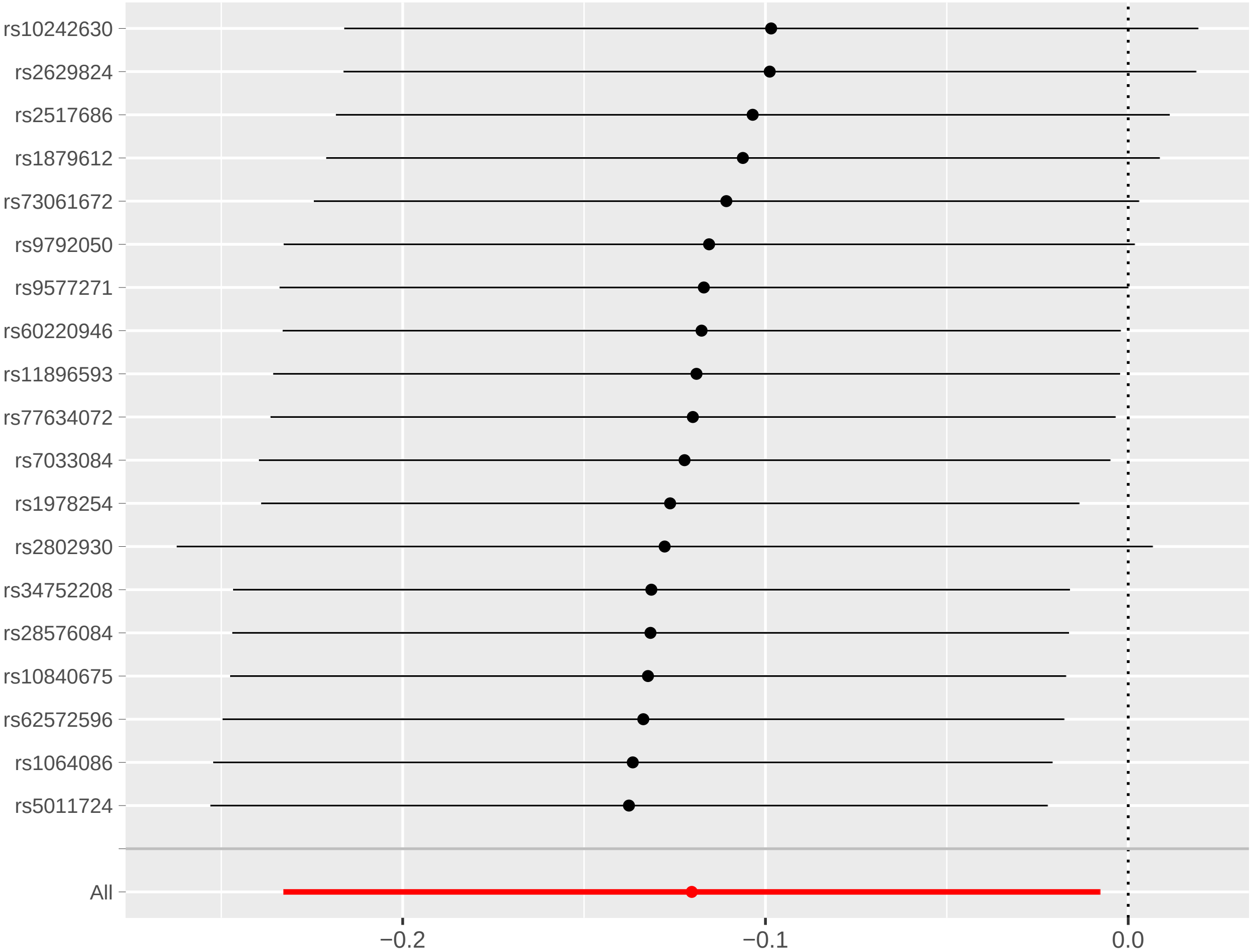

MR leave−one−out sensitivity analysis for
'CD28+ CD45RA+ CD8br %CD8br' on 'Alzheimer disease'

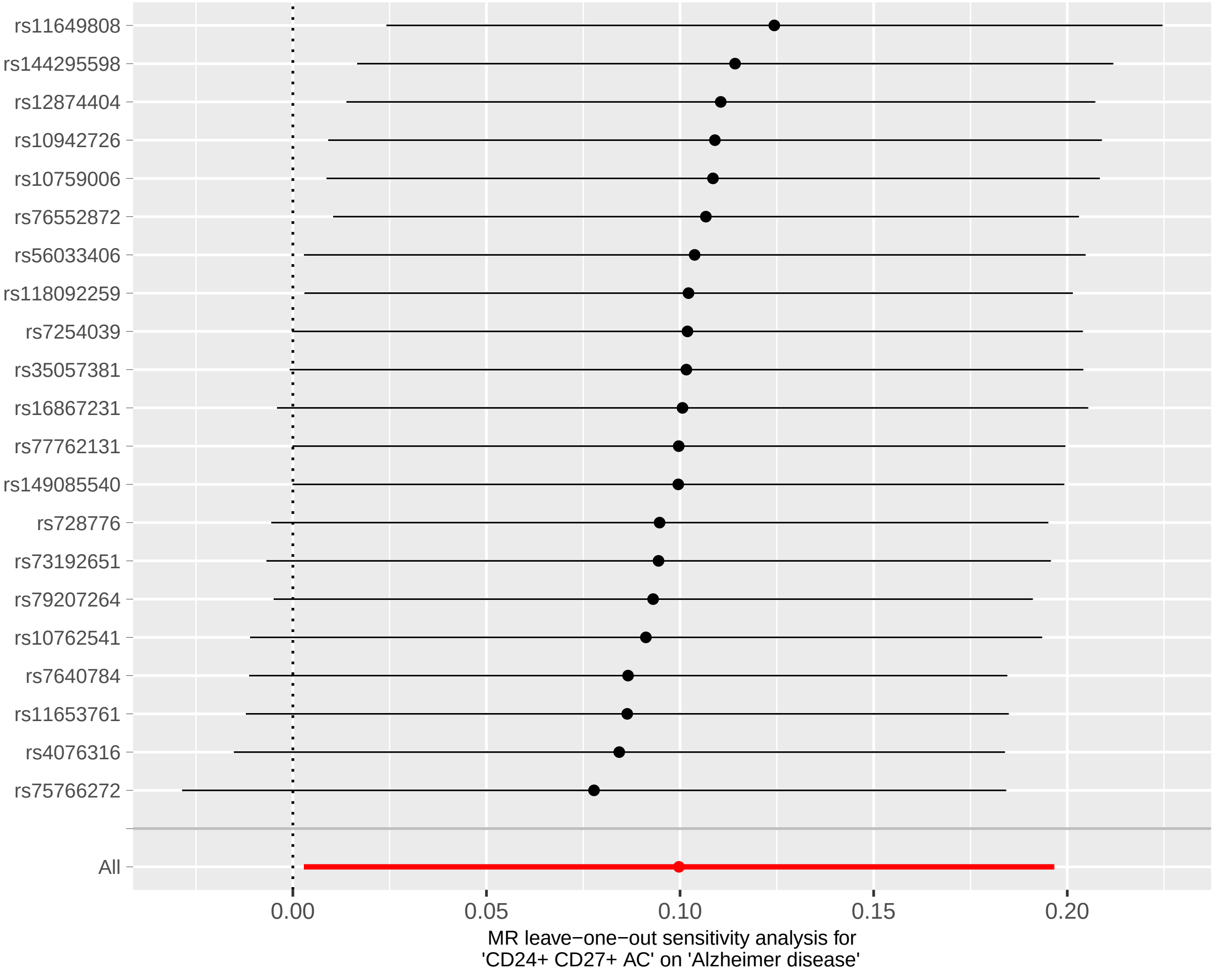

rs11649808
rs144295598
rs12874404
rs10942726
rs10759006
rs76552872
rs56033406
rs118092259
rs7254039
rs35057381
rs16867231
rs77762131
rs149085540
rs728776
rs73192651
rs79207264
rs10762541
rs7640784
rs11653761
rs4076316
rs75766272
All
0.00
0.05
0.10
0.15
0.20
MR leave−one−out sensitivity analysis for
'CD24+ CD27+ AC' on 'Alzheimer disease'

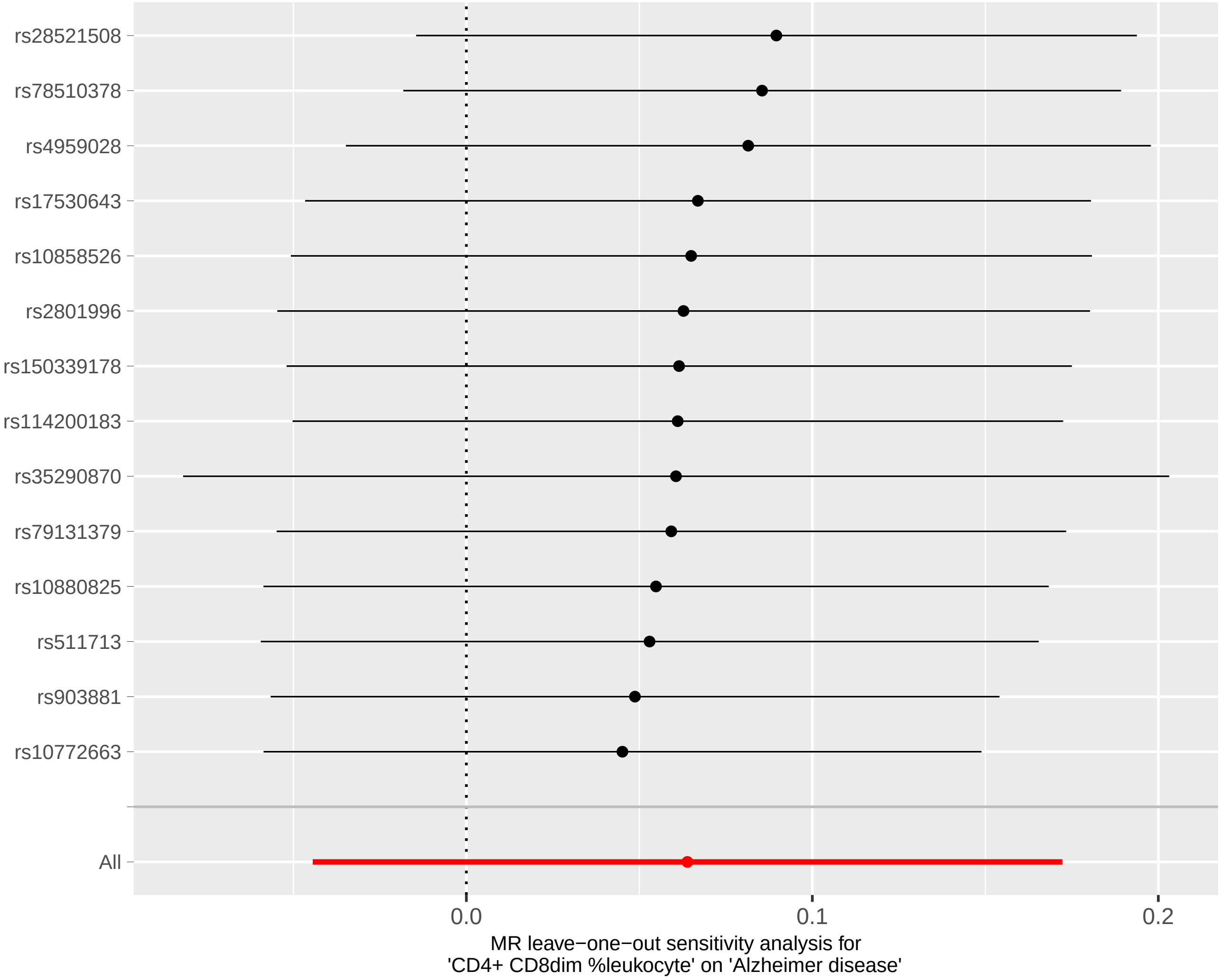
rs28521508
rs78510378
rs4959028
rs17530643
rs10858526
rs2801996
rs150339178
rs114200183
rs35290870
rs79131379
rs10880825
rs511713
rs903881
rs10772663
All
0.0
0.1
0.2
MR leave−one−out sensitivity analysis for
'CD4+ CD8dim %leukocyte' on 'Alzheimer disease'

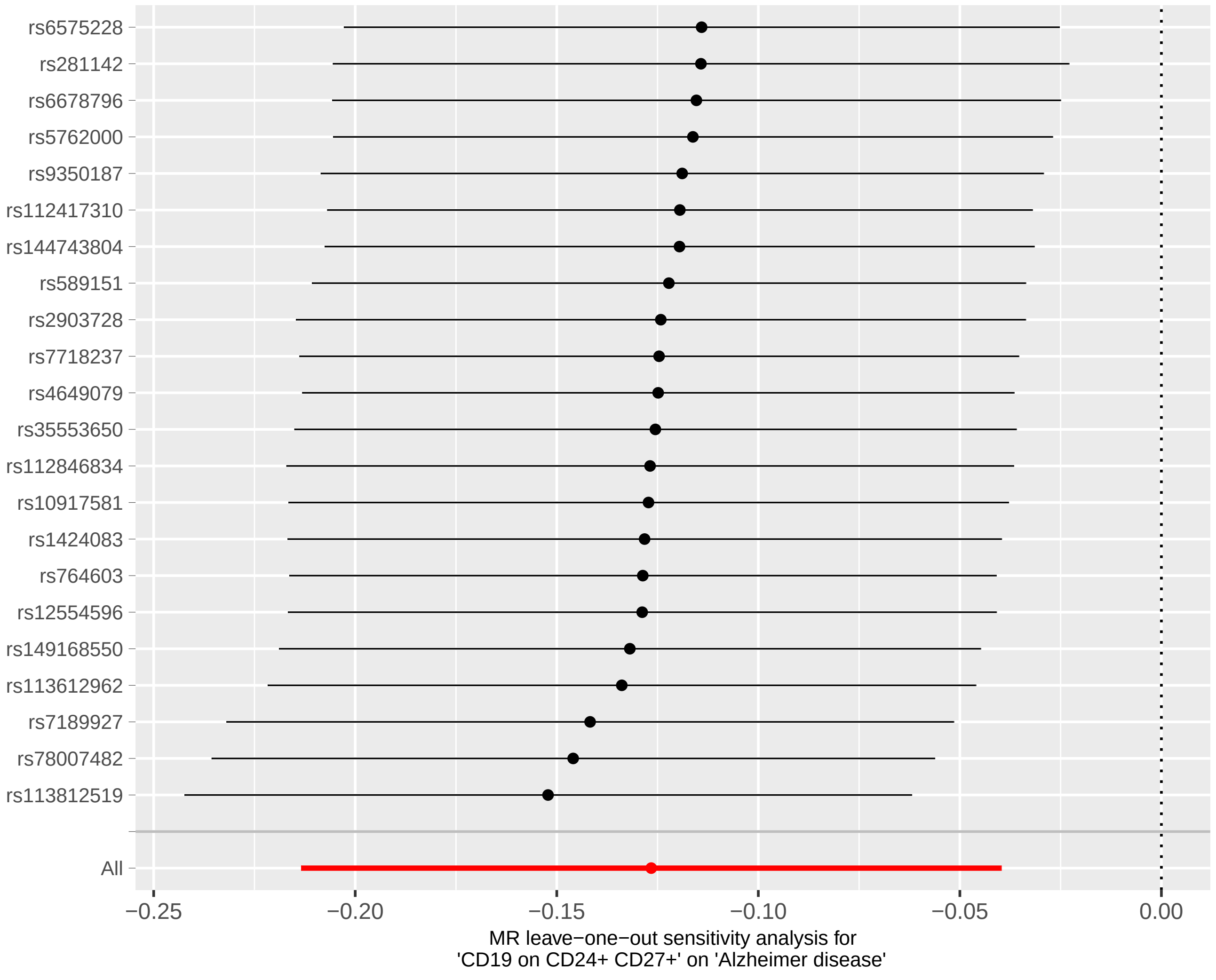
rs6575228
rs281142
rs6678796
rs5762000
rs9350187
rs112417310
rs144743804
rs589151
rs2903728
rs7718237
rs4649079
rs35553650
rs112846834
rs10917581
rs1424083
rs764603
rs12554596
rs149168550
rs113612962
rs7189927
rs78007482
rs113812519
All
−0.25
−0.20
−0.15
−0.10
−0.05
0.00
MR leave−one−out sensitivity analysis for
'CD19 on CD24+ CD27+' on 'Alzheimer disease'

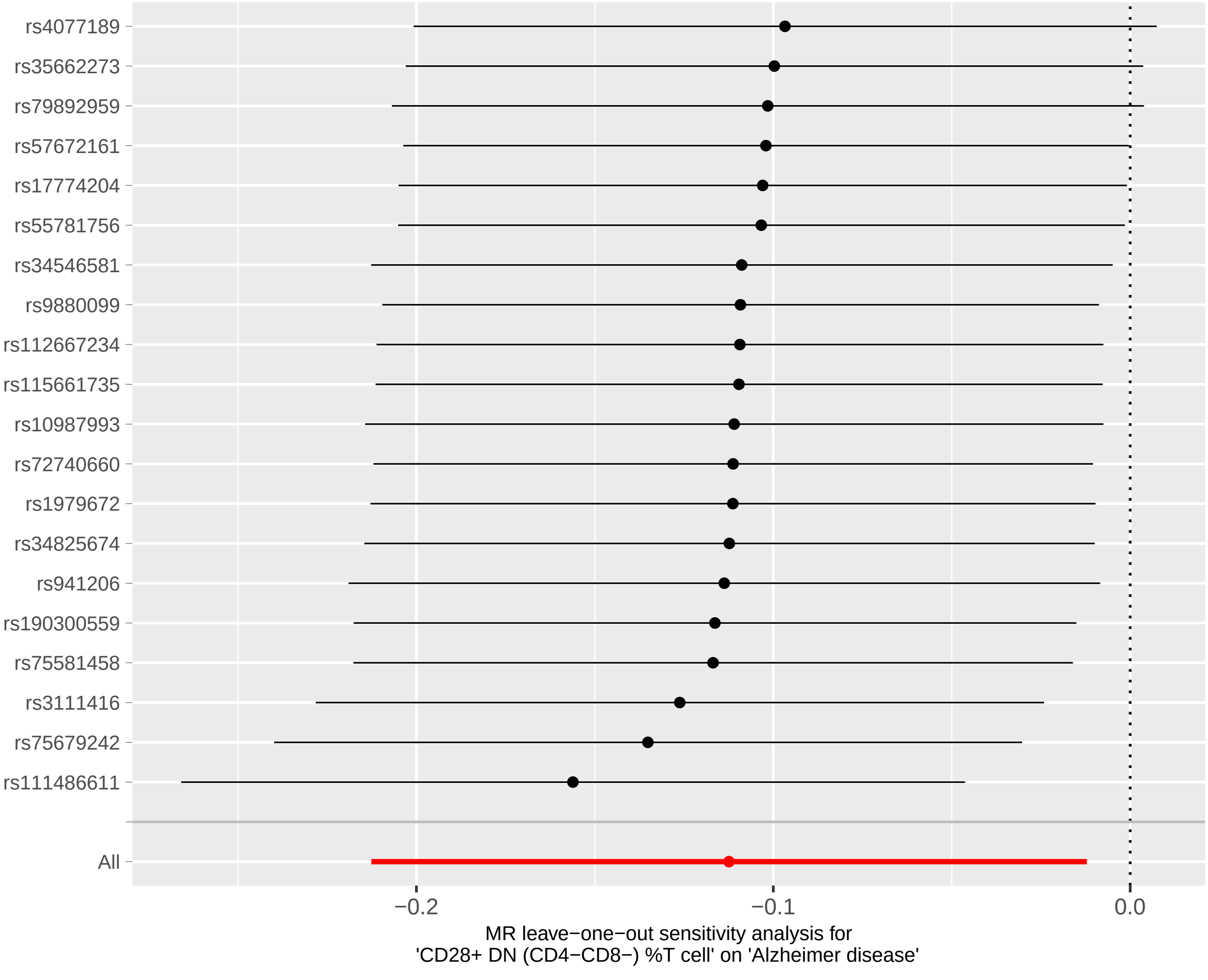
rs4077189
rs35662273
rs79892959
rs57672161
rs17774204
rs55781756
rs34546581
rs9880099
rs112667234
rs115661735
rs10987993
rs72740660
rs1979672
rs34825674
rs941206
rs190300559
rs75581458
rs3111416
rs75679242
rs111486611
All
−0.2
−0.1
0.0
MR leave−one−out sensitivity analysis for
'CD28+ DN (CD4−CD8−) %T cell' on 'Alzheimer disease'

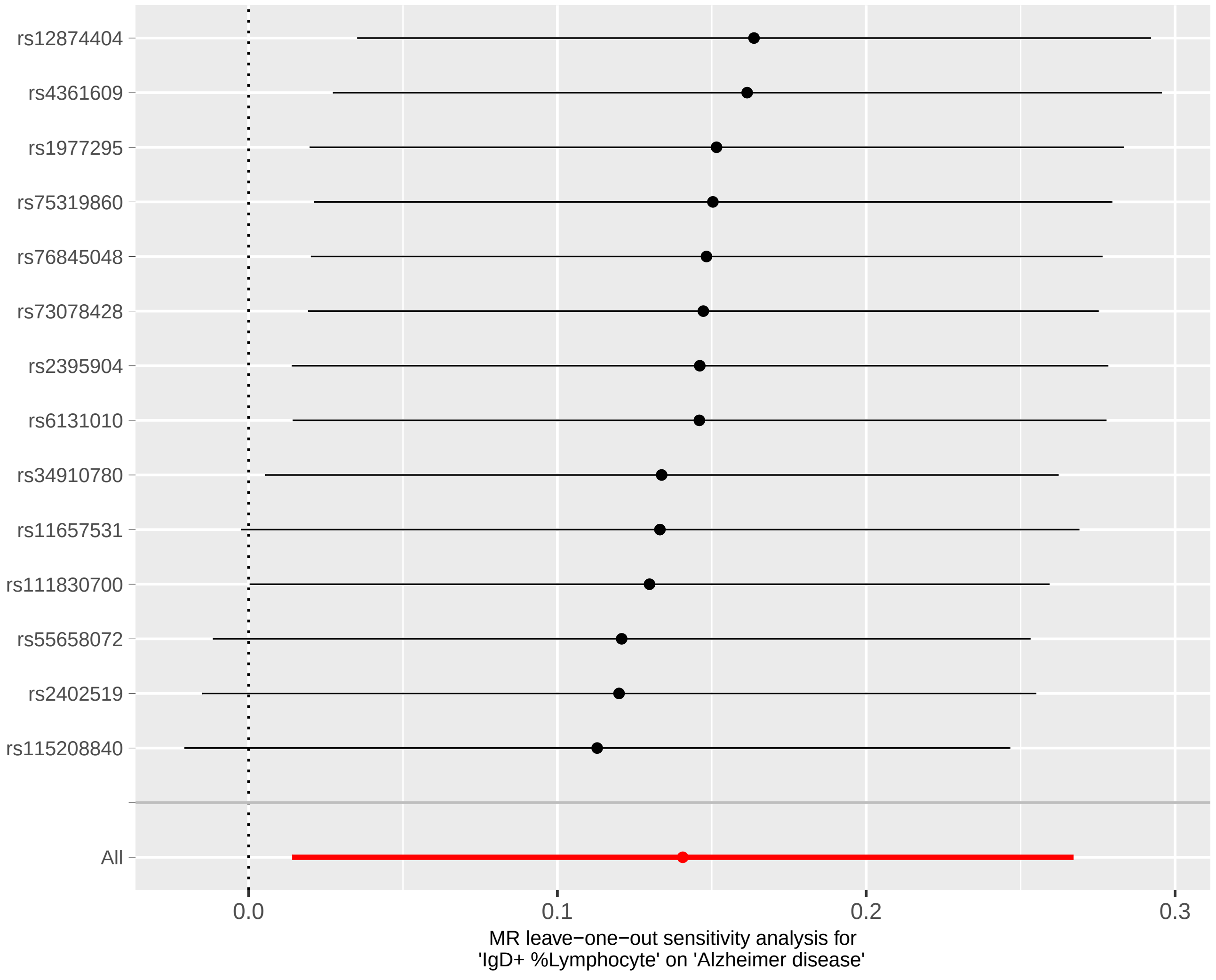
rs12874404
rs4361609
rs1977295
rs75319860
rs76845048
rs73078428
rs2395904
rs6131010
rs34910780
rs11657531
rs111830700
rs55658072
rs2402519
rs115208840
All
0.0
0.1
0.2
0.3
MR leave−one−out sensitivity analysis for
'IgD+ %Lymphocyte' on 'Alzheimer disease'

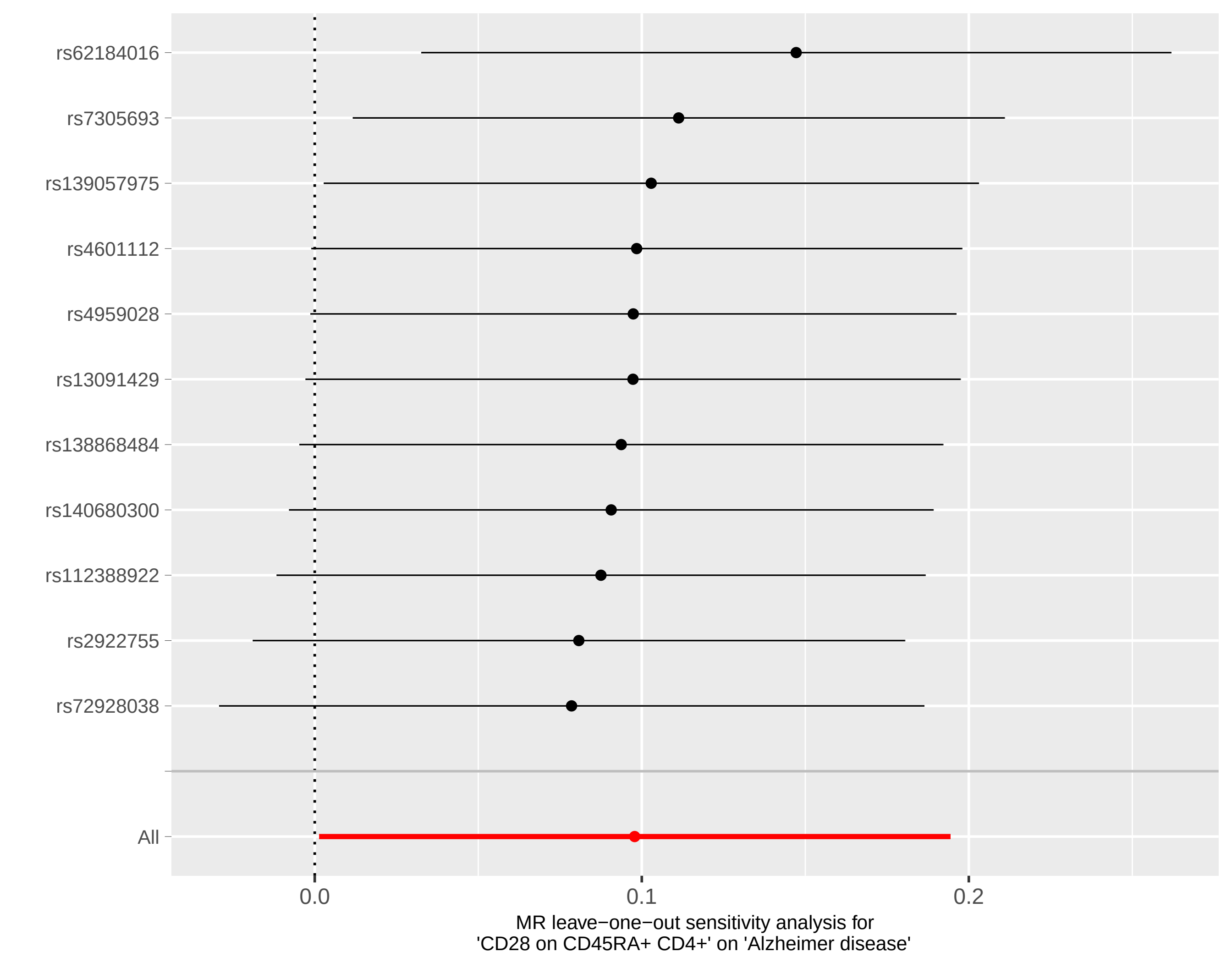

rs62184016
rs7305693
rs139057975
rs4601112
rs4959028
rs13091429
rs138868484
rs140680300
rs112388922
rs2922755
rs72928038
All
0.0
0.1
0.2
MR leave−one−out sensitivity analysis for
'CD28 on CD45RA+ CD4+' on 'Alzheimer disease'

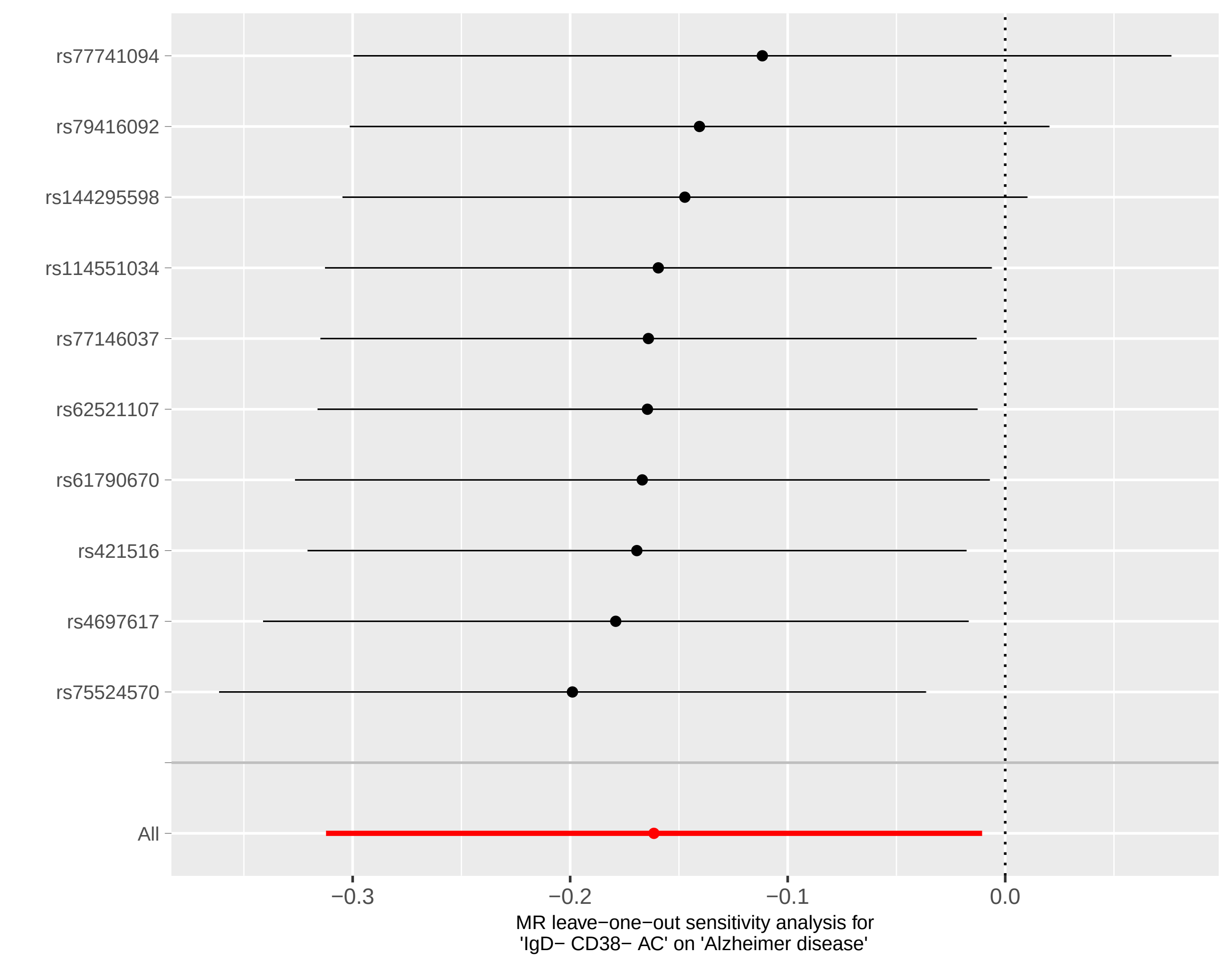
rs77741094
rs79416092
rs144295598
rs114551034
rs77146037
rs62521107
rs61790670
rs421516
rs4697617
rs75524570
All
−0.3
−0.2
−0.1
0.0
MR leave−one−out sensitivity analysis for
'IgD− CD38− AC' on 'Alzheimer disease'

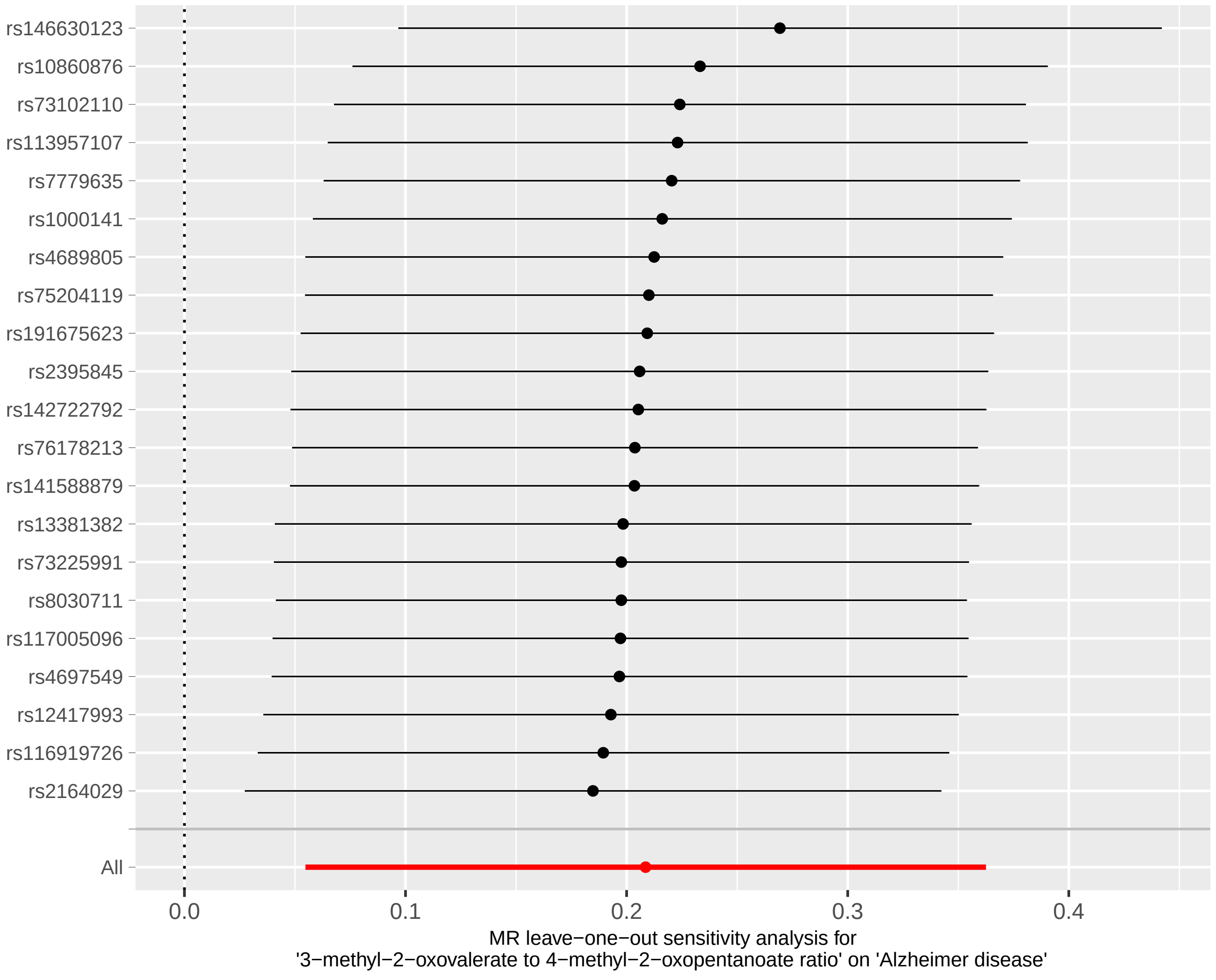
rs146630123
rs10860876
rs73102110
rs113957107
rs7779635
rs1000141
rs4689805
rs75204119
rs191675623
rs2395845
rs142722792
rs76178213
rs141588879
rs13381382
rs73225991
rs8030711
rs117005096
rs4697549
rs12417993
rs116919726
rs2164029
All
0.0
0.1
0.2
0.3
0.4
MR leave−one−out sensitivity analysis for
'3−methyl−2−oxovalerate to 4−methyl−2−oxopentanoate ratio' on 'Alzheimer disease'

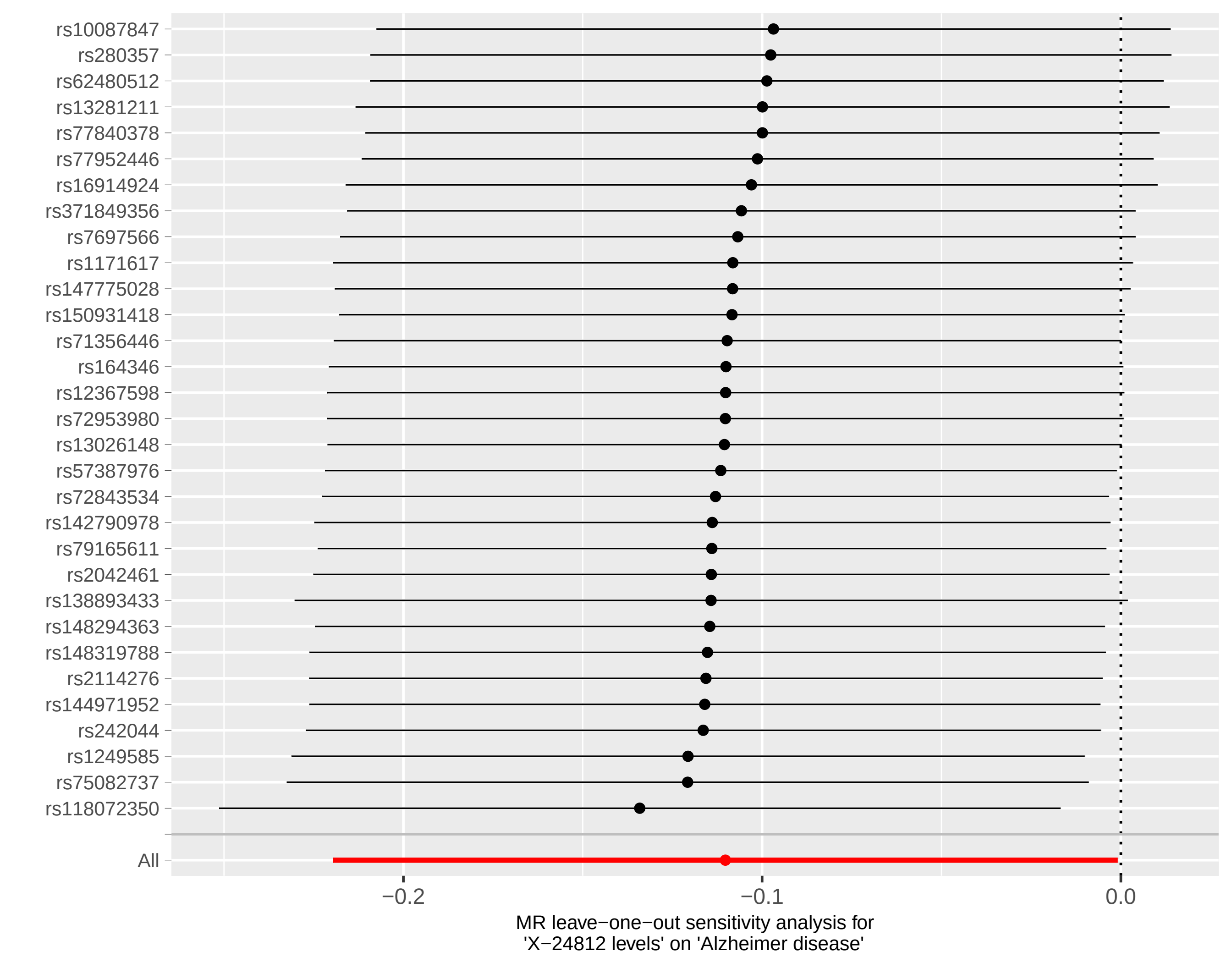
rs10087847
rs280357
rs62480512
rs13281211
rs77840378
rs77952446
rs16914924
rs371849356
rs7697566
rs1171617
rs147775028
rs150931418
rs71356446
rs164346
rs12367598
rs72953980
rs13026148
rs57387976
rs72843534
rs142790978
rs79165611
rs2042461
rs138893433
rs148294363
rs148319788
rs2114276
rs144971952
rs242044
rs1249585
rs75082737
rs118072350
All
−0.2
−0.1
0.0
MR leave−one−out sensitivity analysis for
'X−24812 levels' on 'Alzheimer disease'

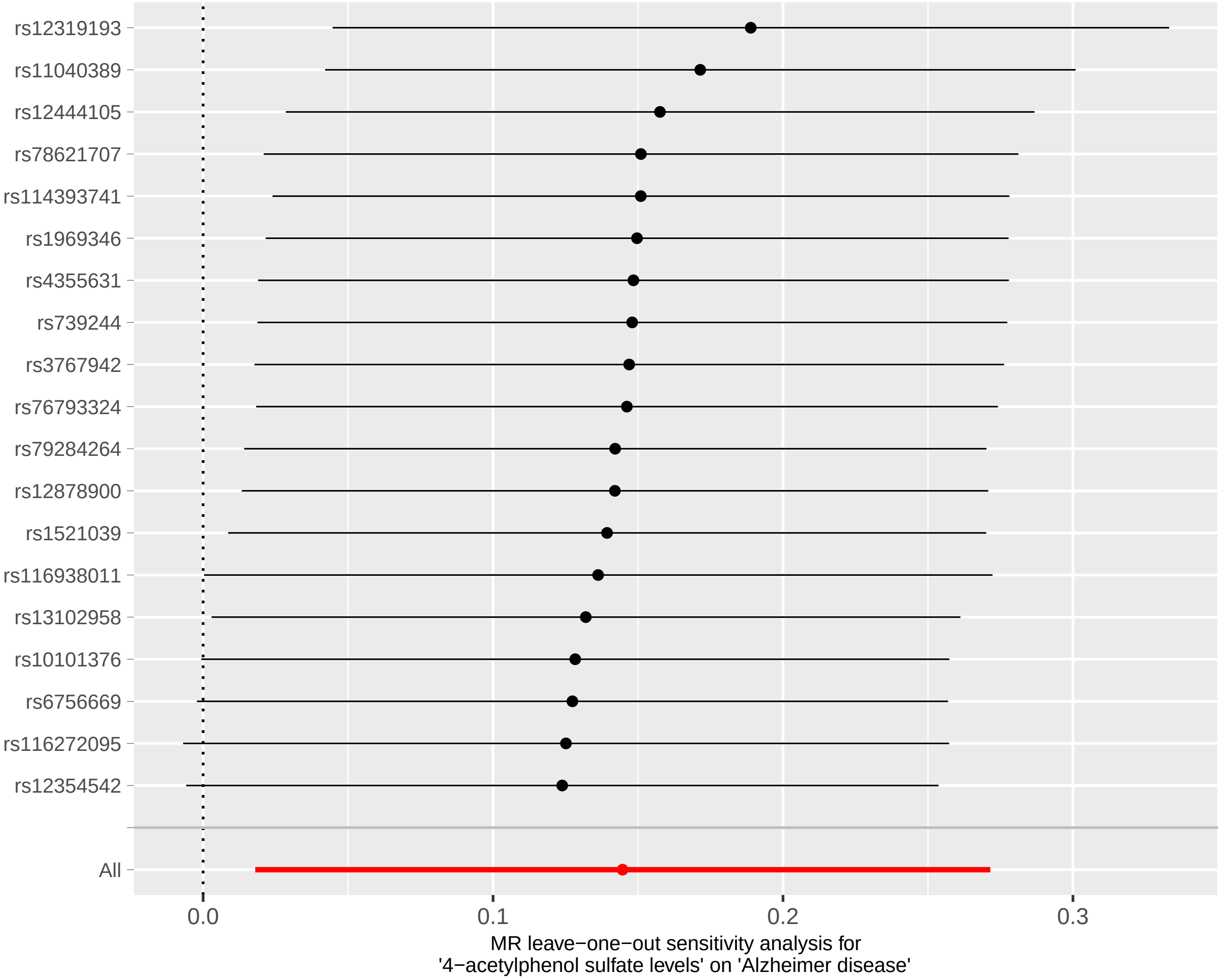
rs12319193
rs11040389
rs12444105
rs78621707
rs114393741
rs1969346
rs4355631
rs739244
rs3767942
rs76793324
rs79284264
rs12878900
rs1521039
rs116938011
rs13102958
rs10101376
rs6756669
rs116272095
rs12354542
All
0.0
0.1
0.2
0.3
MR leave−one−out sensitivity analysis for
'4−acetylphenol sulfate levels' on 'Alzheimer disease'

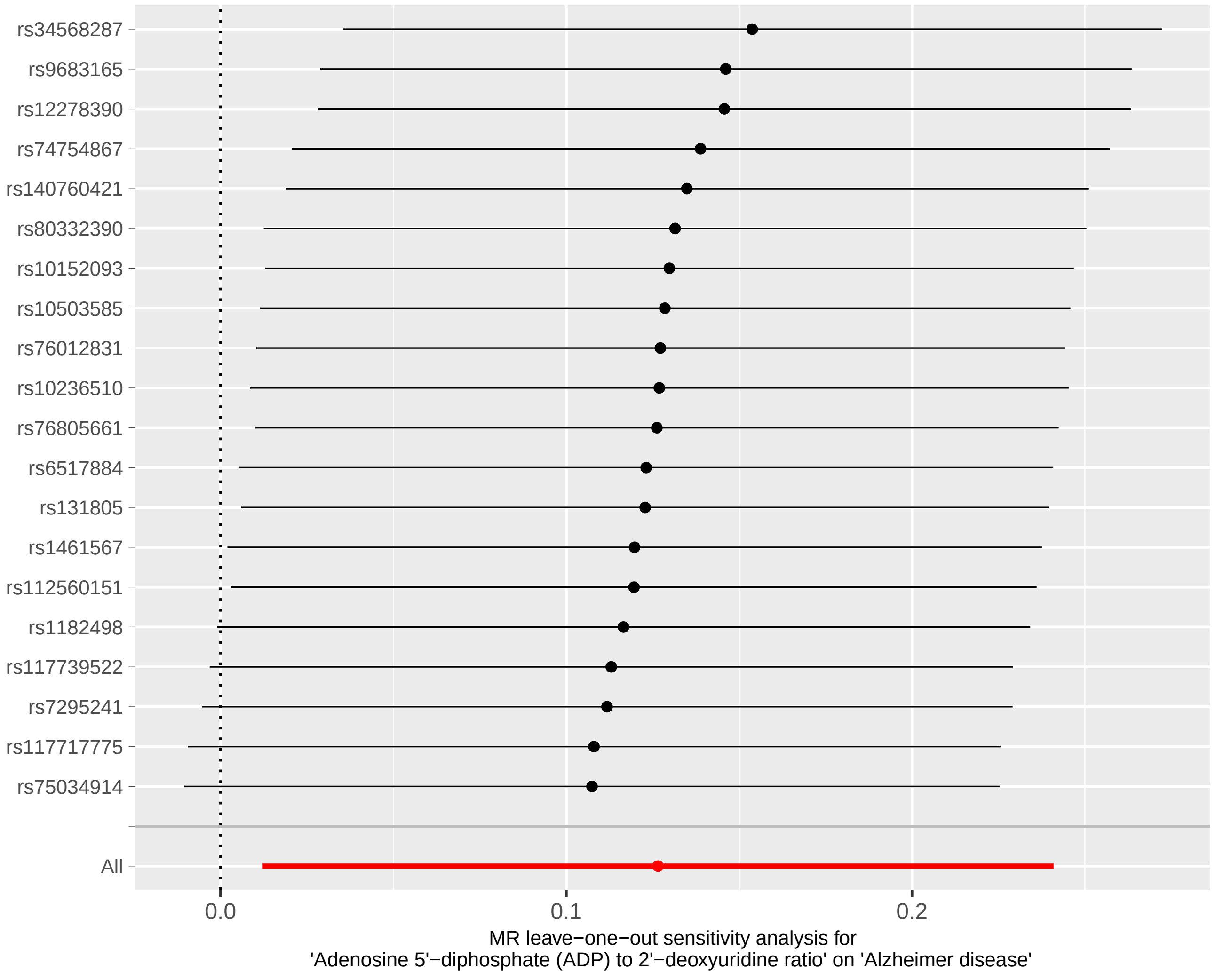
rs34568287
rs9683165
rs12278390
rs74754867
rs140760421
rs80332390
rs10152093
rs10503585
rs76012831
rs10236510
rs76805661
rs6517884
rs131805
rs1461567
rs112560151
rs1182498
rs117739522
rs7295241
rs117717775
rs75034914
All
0.0
0.1
0.2
MR leave−one−out sensitivity analysis for
'Adenosine 5'−diphosphate (ADP) to 2'−deoxyuridine ratio' on 'Alzheimer disease'

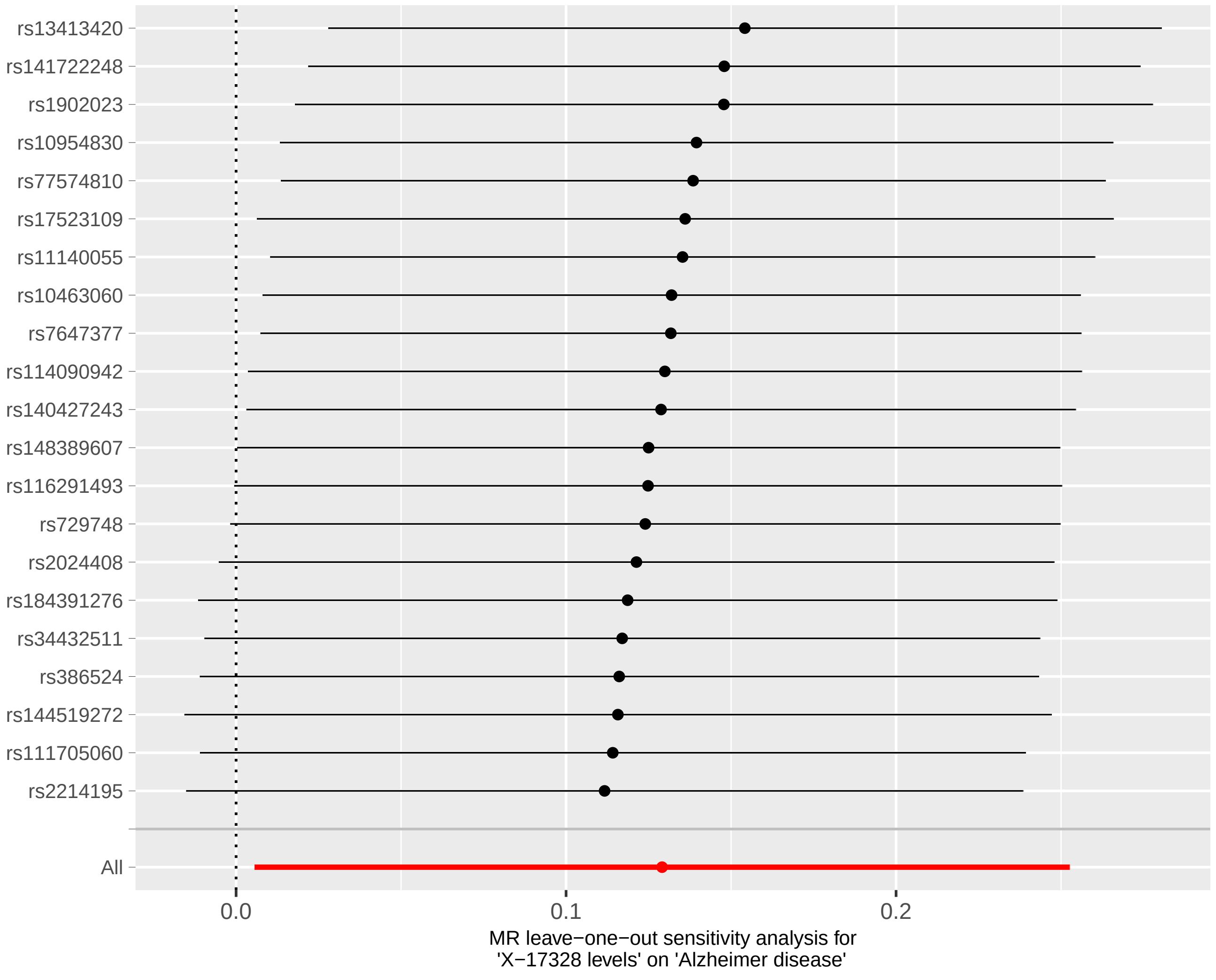

rs13413420
rs141722248
rs1902023
rs10954830
rs77574810
rs17523109
rs11140055
rs10463060
rs7647377
rs114090942
rs140427243
rs148389607
rs116291493
rs729748
rs2024408
rs184391276
rs34432511
rs386524
rs144519272
rs111705060
rs2214195
All
0.0
0.1
0.2
MR leave−one−out sensitivity analysis for
'X−17328 levels' on 'Alzheimer disease'

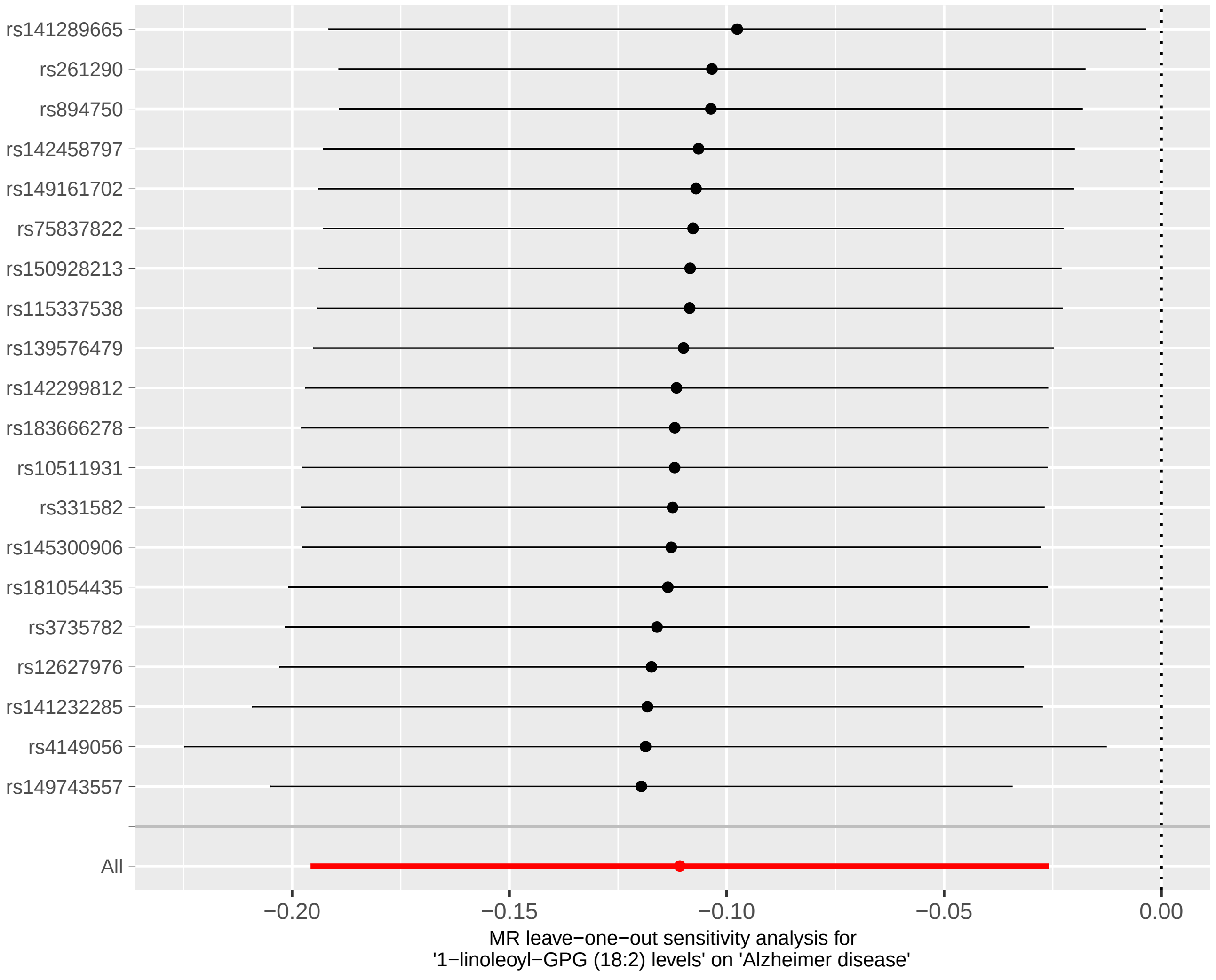

rs141289665
rs261290
rs894750
rs142458797
rs149161702
rs75837822
rs150928213
rs115337538
rs139576479
rs142299812
rs183666278
rs10511931
rs331582
rs145300906
rs181054435
rs3735782
rs12627976
rs141232285
rs4149056
rs149743557
All
−0.20
−0.15
−0.10
−0.05
0.00
MR leave−one−out sensitivity analysis for
'1−linoleoyl−GPG (18:2) levels' on 'Alzheimer disease'

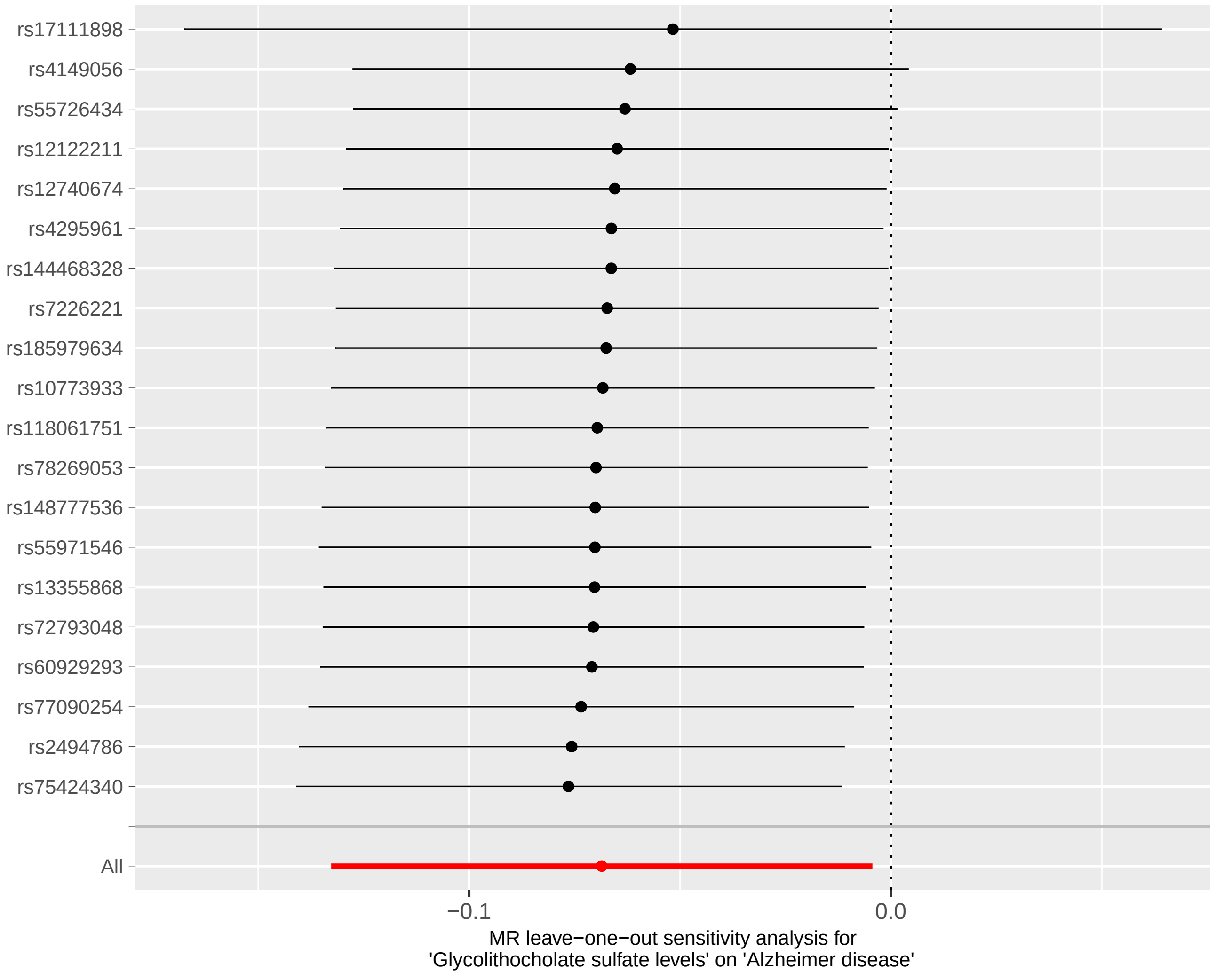
rs17111898
rs4149056
rs55726434
rs12122211
rs12740674
rs4295961
rs144468328
rs7226221
rs185979634
rs10773933
rs118061751
rs78269053
rs148777536
rs55971546
rs13355868
rs72793048
rs60929293
rs77090254
rs2494786
rs75424340
All
−0.1
0.0
MR leave−one−out sensitivity analysis for
'Glycolithocholate sulfate levels' on 'Alzheimer disease'

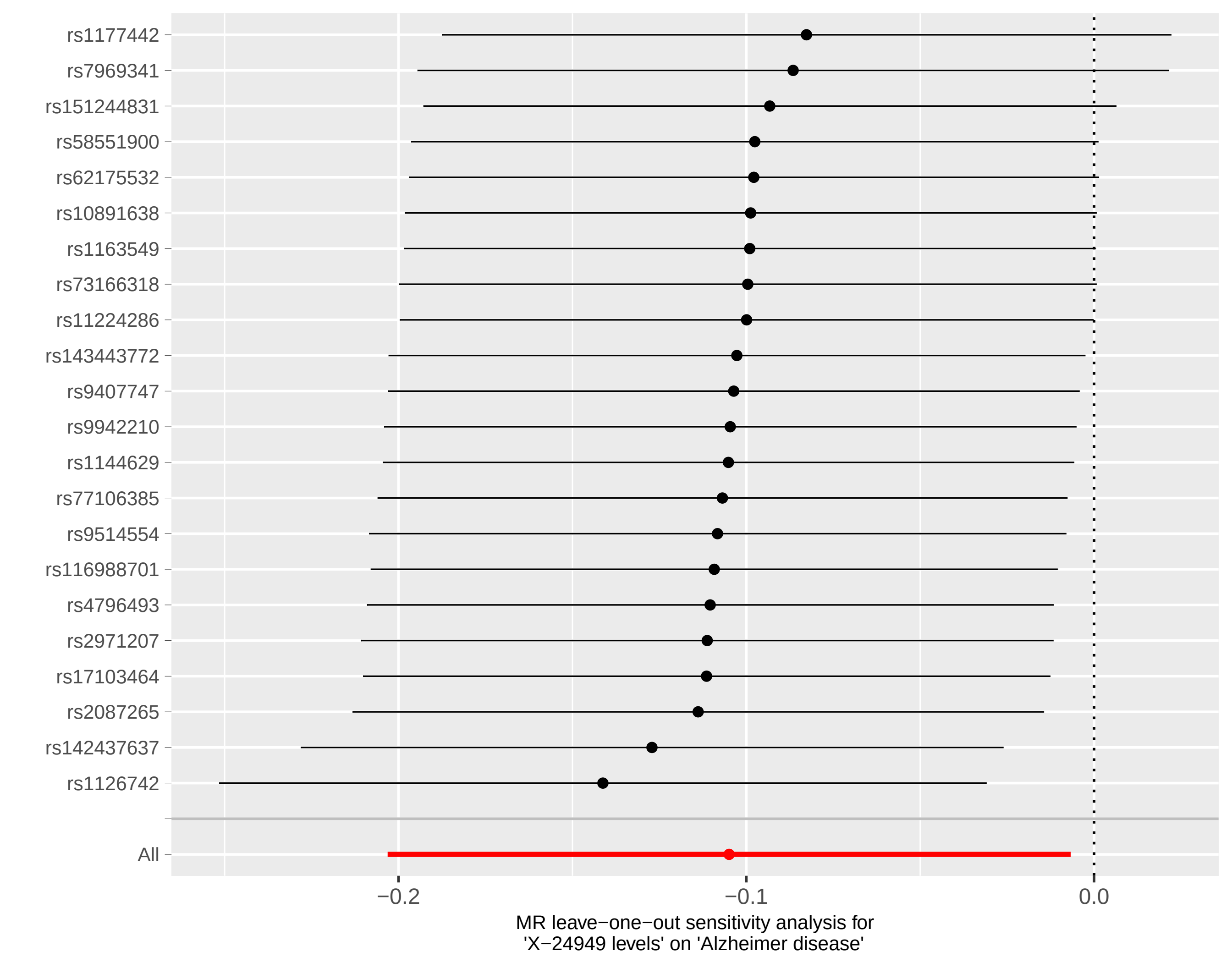

rs1177442
rs7969341
rs151244831
rs58551900
rs62175532
rs10891638
rs1163549
rs73166318
rs11224286
rs143443772
rs9407747
rs9942210
rs1144629
rs77106385
rs9514554
rs116988701
rs4796493
rs2971207
rs17103464
rs2087265
rs142437637
rs1126742
All
−0.2
−0.1
0.0
MR leave−one−out sensitivity analysis for
'X−24949 levels' on 'Alzheimer disease'

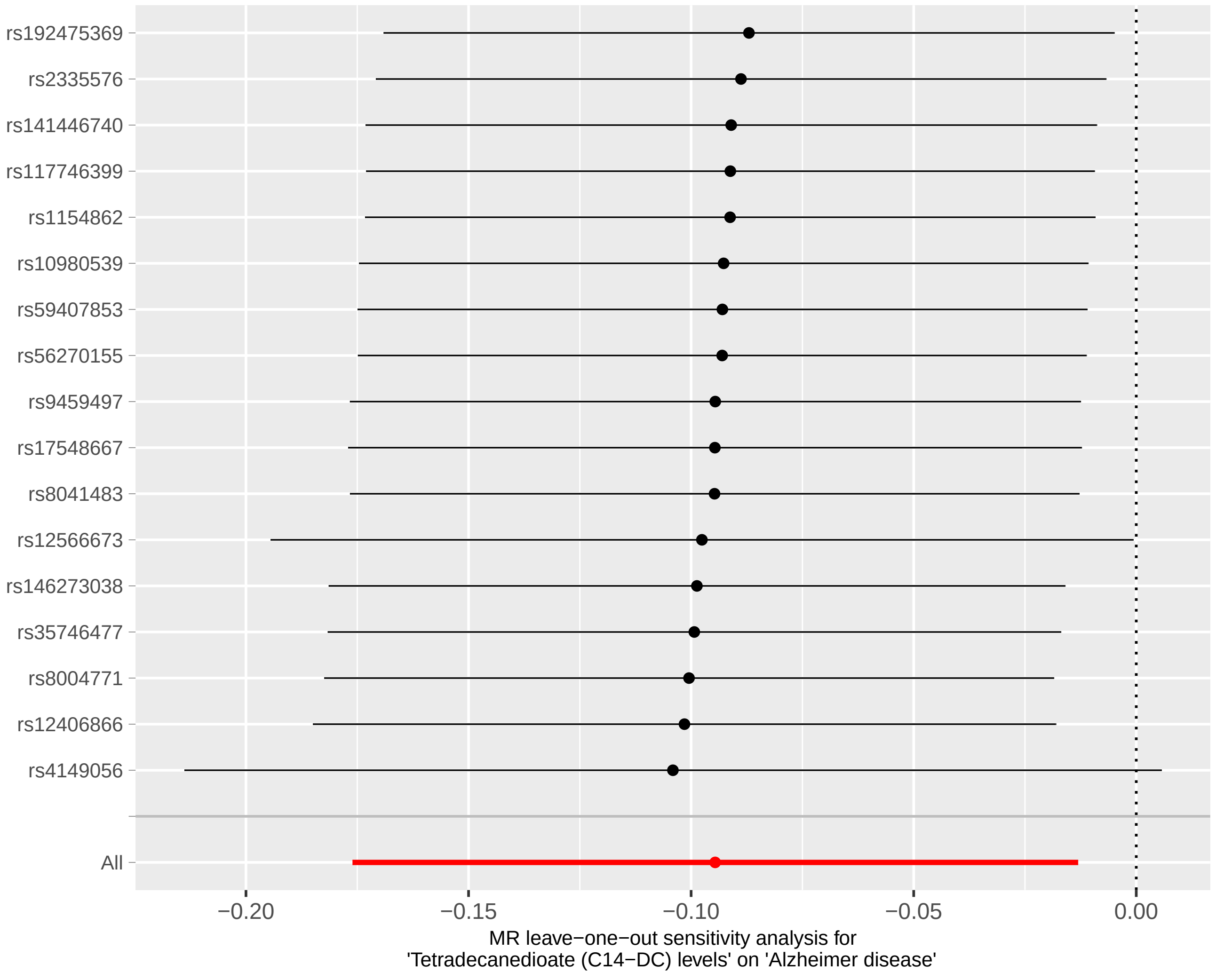
rs192475369
rs2335576
rs141446740
rs117746399
rs1154862
rs10980539
rs59407853
rs56270155
rs9459497
rs17548667
rs8041483
rs12566673
rs146273038
rs35746477
rs8004771
rs12406866
rs4149056
All
−0.20
−0.15
−0.10
−0.05
0.00
MR leave−one−out sensitivity analysis for
'Tetradecanedioate (C14−DC) levels' on 'Alzheimer disease'

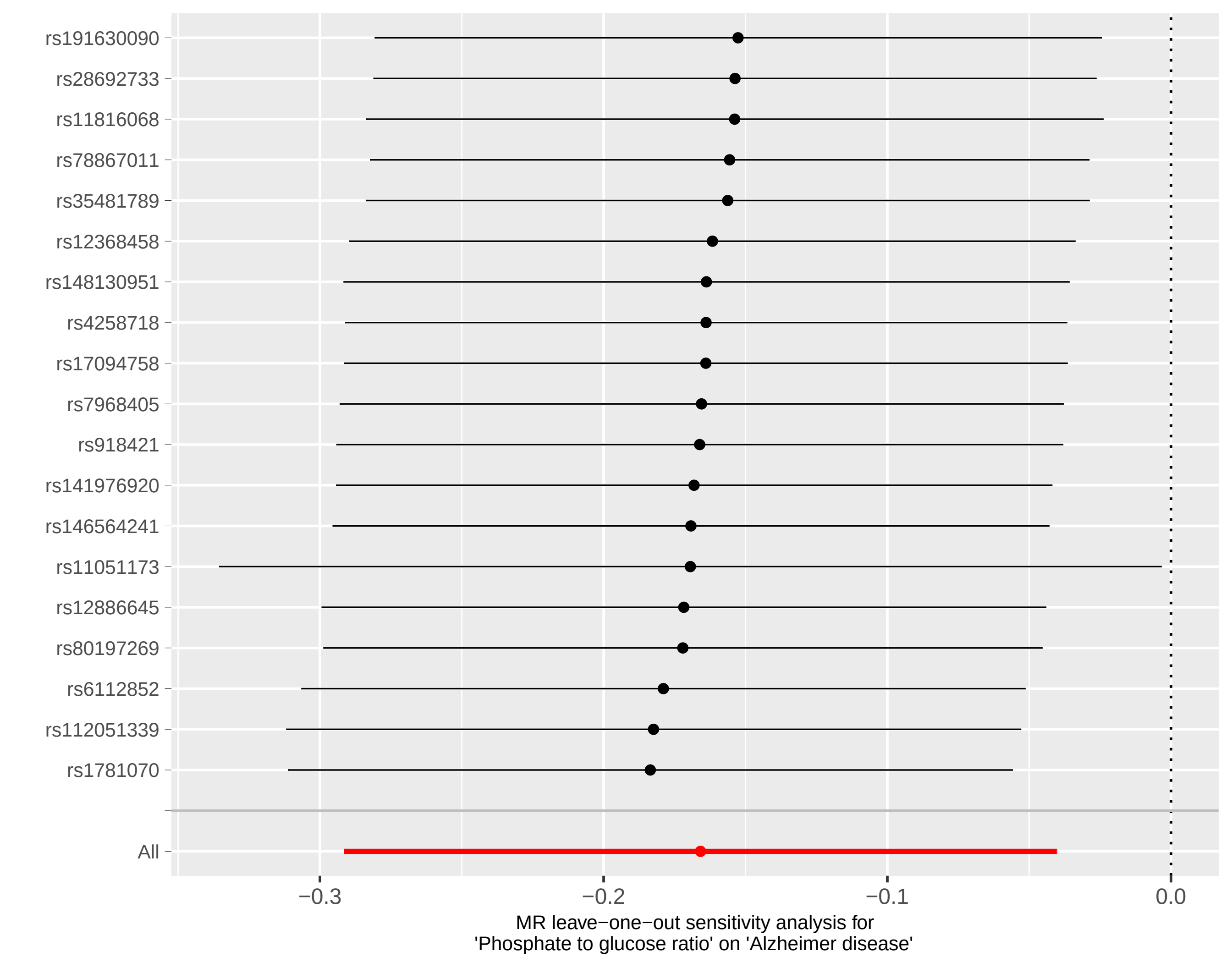
rs191630090
rs28692733
rs11816068
rs78867011
rs35481789
rs12368458
rs148130951
rs4258718
rs17094758
rs7968405
rs918421
rs141976920
rs146564241
rs11051173
rs12886645
rs80197269
rs6112852
rs112051339
rs1781070
All
−0.3
−0.2
−0.1
0.0
MR leave−one−out sensitivity analysis for
'Phosphate to glucose ratio' on 'Alzheimer disease'

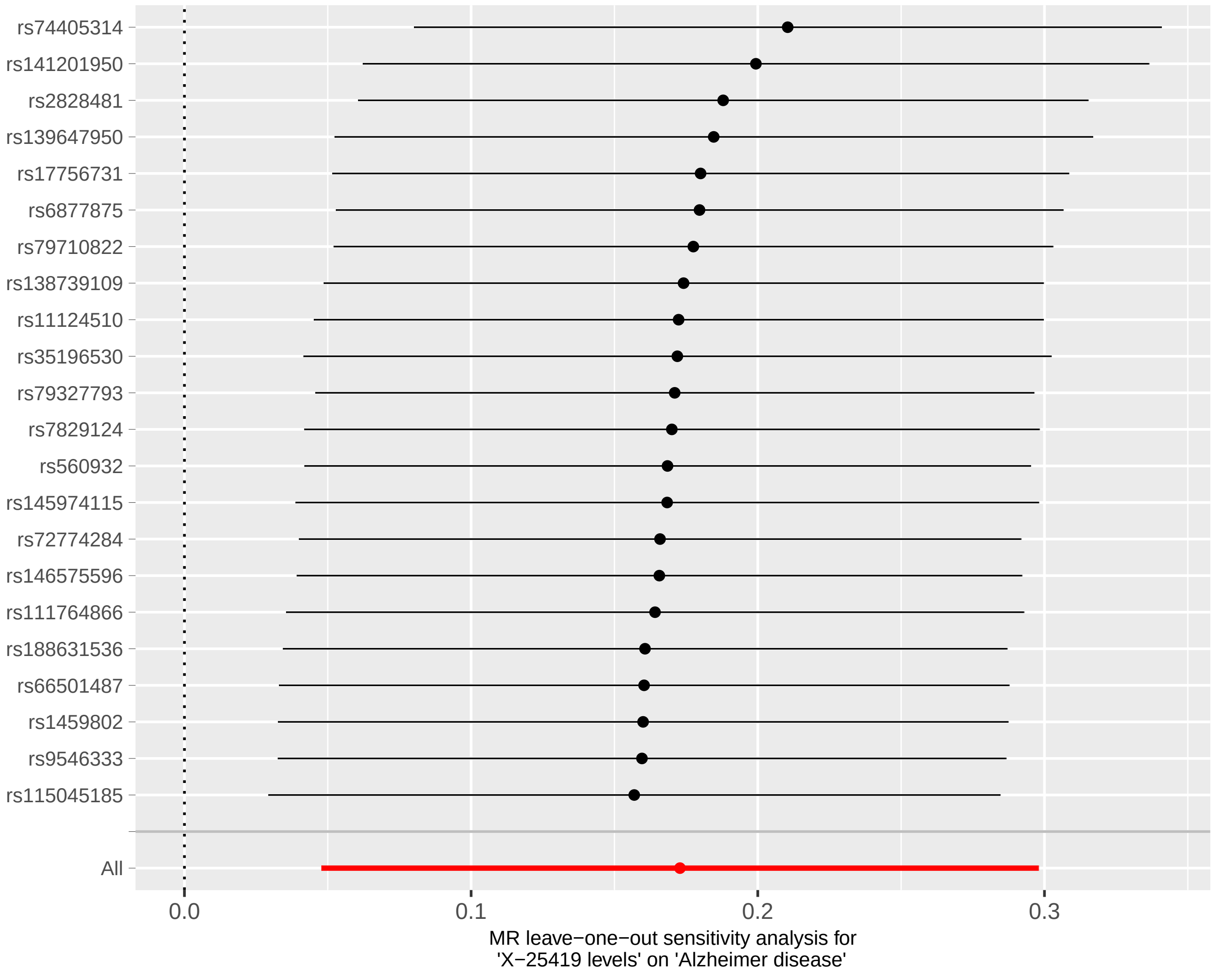
rs74405314
rs141201950
rs2828481
rs139647950
rs17756731
rs6877875
rs79710822
rs138739109
rs11124510
rs35196530
rs79327793
rs7829124
rs560932
rs145974115
rs72774284
rs146575596
rs111764866
rs188631536
rs66501487
rs1459802
rs9546333
rs115045185
All
0.0
0.1
0.2
0.3
MR leave−one−out sensitivity analysis for
'X−25419 levels' on 'Alzheimer disease'

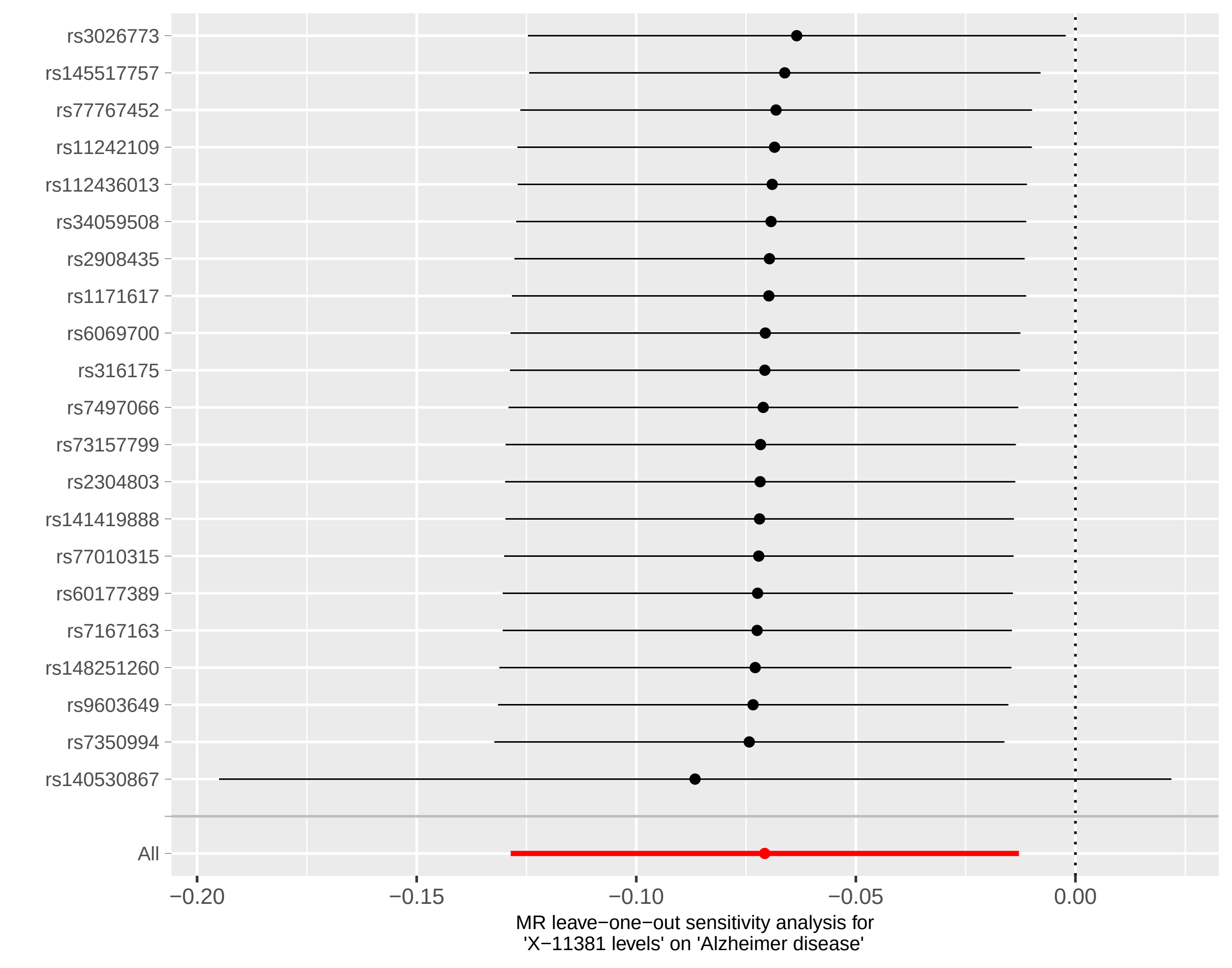
rs3026773
rs145517757
rs77767452
rs11242109
rs112436013
rs34059508
rs2908435
rs1171617
rs6069700
rs316175
rs7497066
rs73157799
rs2304803
rs141419888
rs77010315
rs60177389
rs7167163
rs148251260
rs9603649
rs7350994
rs140530867
All
−0.20
−0.15
−0.10
−0.05
0.00
MR leave−one−out sensitivity analysis for
'X−11381 levels' on 'Alzheimer disease'

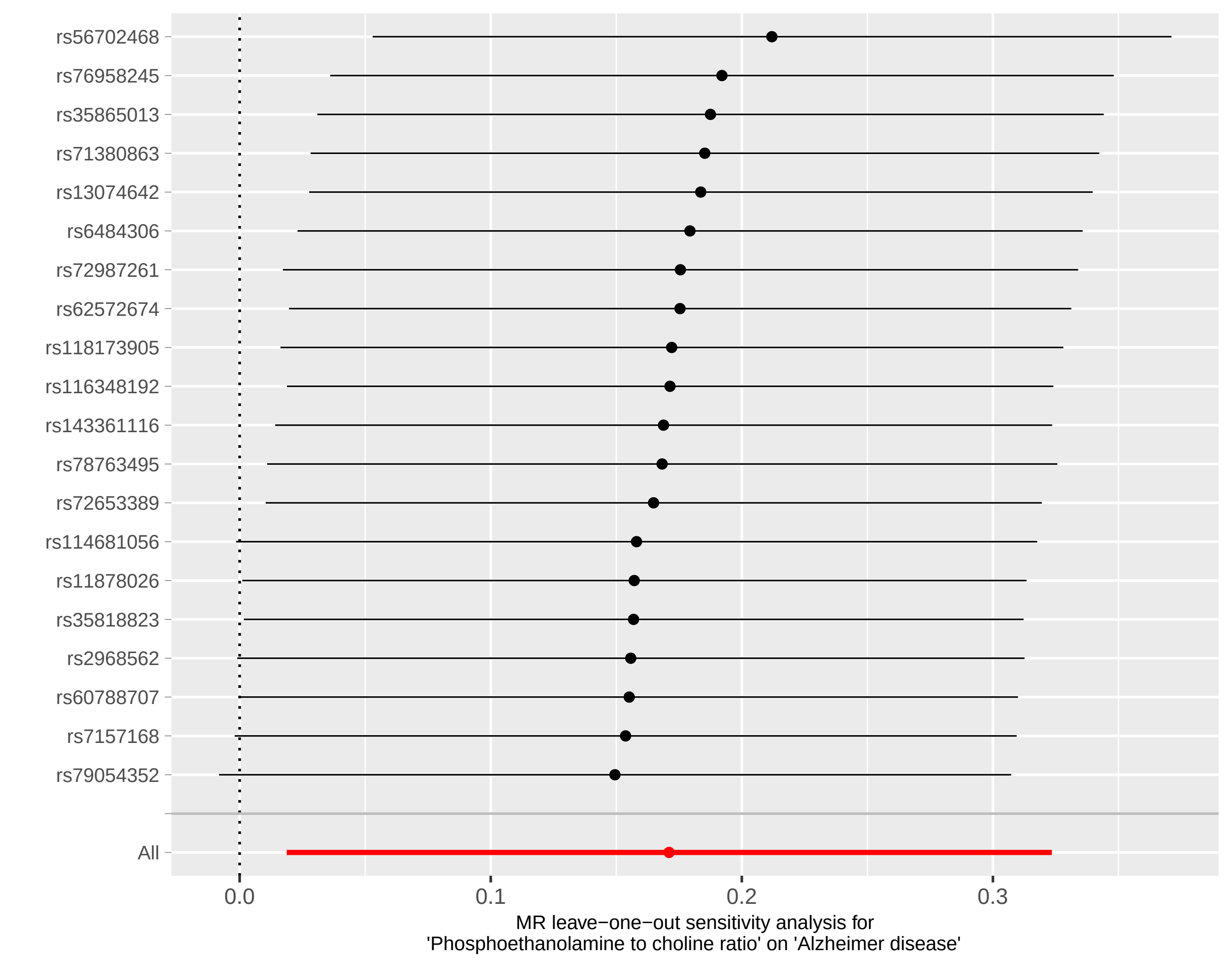
rs56702468
rs76958245
rs35865013
rs71380863
rs13074642
rs6484306
rs72987261
rs62572674
rs118173905
rs116348192
rs143361116
rs78763495
rs72653389
rs114681056
rs11878026
rs35818823
rs2968562
rs60788707
rs7157168
rs79054352
All
0.0
0.1
0.2
0.3
MR leave−one−out sensitivity analysis for
'Phosphoethanolamine to choline ratio' on 'Alzheimer disease'

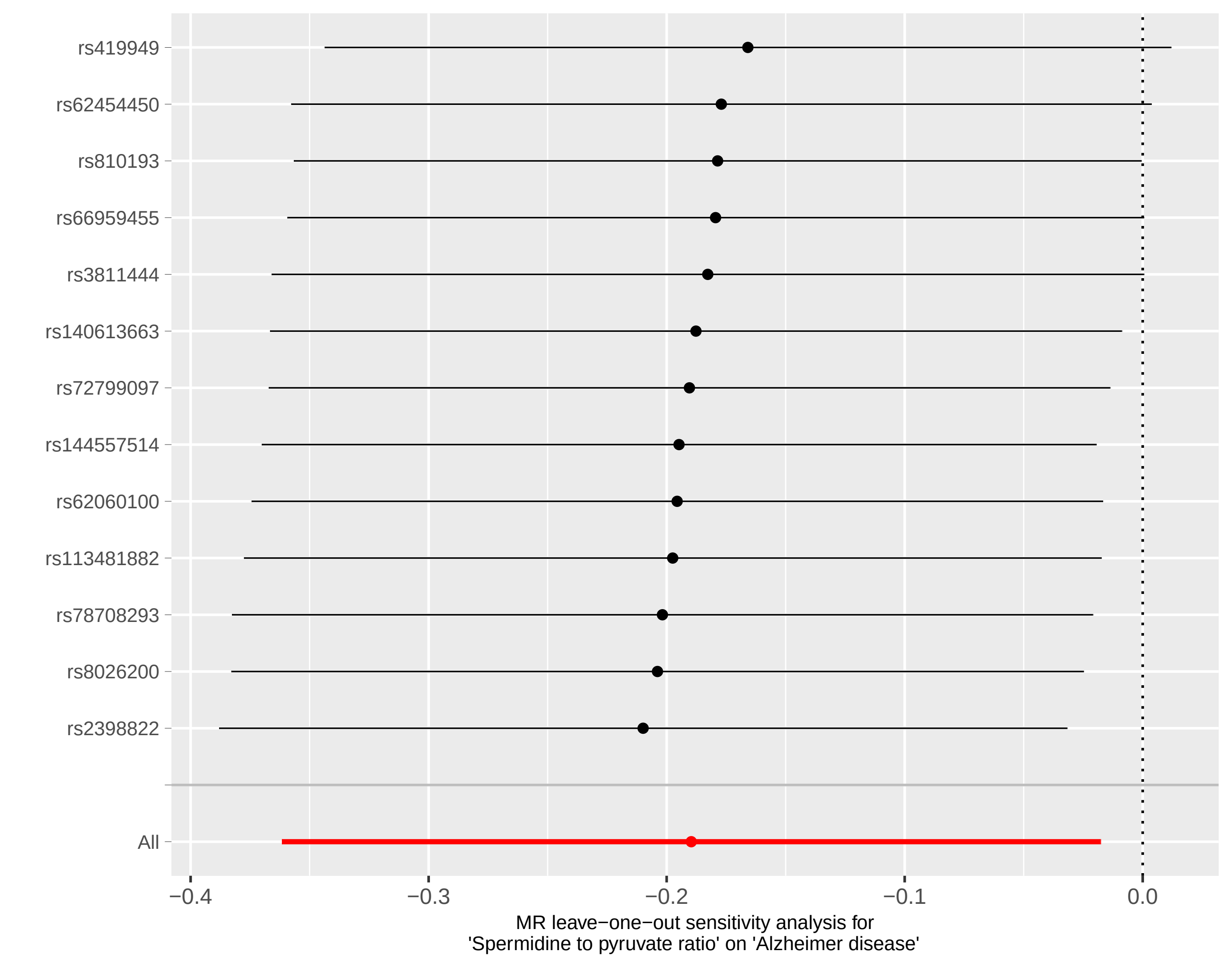

rs419949
rs62454450
rs810193
rs66959455
rs3811444
rs140613663
rs72799097
rs144557514
rs62060100
rs113481882
rs78708293
rs8026200
rs2398822
All
−0.4
−0.3
−0.2
−0.1
0.0
MR leave−one−out sensitivity analysis for
'Spermidine to pyruvate ratio' on 'Alzheimer disease'

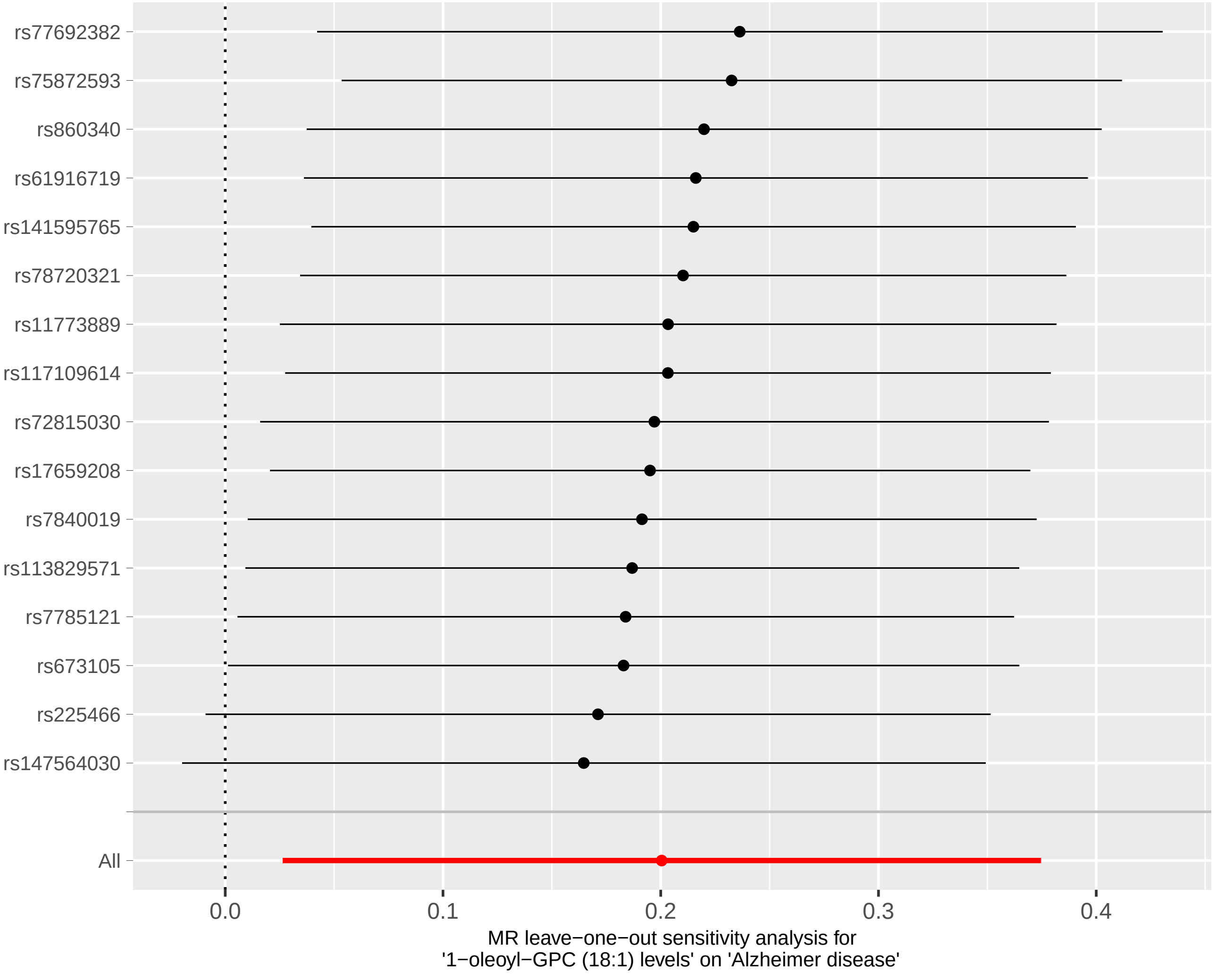
rs77692382
rs75872593
rs860340
rs61916719
rs141595765
rs78720321
rs11773889
rs117109614
rs72815030
rs17659208
rs7840019
rs113829571
rs7785121
rs673105
rs225466
rs147564030
All
0.0
0.1
0.2
0.3
0.4
MR leave−one−out sensitivity analysis for
'1−oleoyl−GPC (18:1) levels' on 'Alzheimer disease'

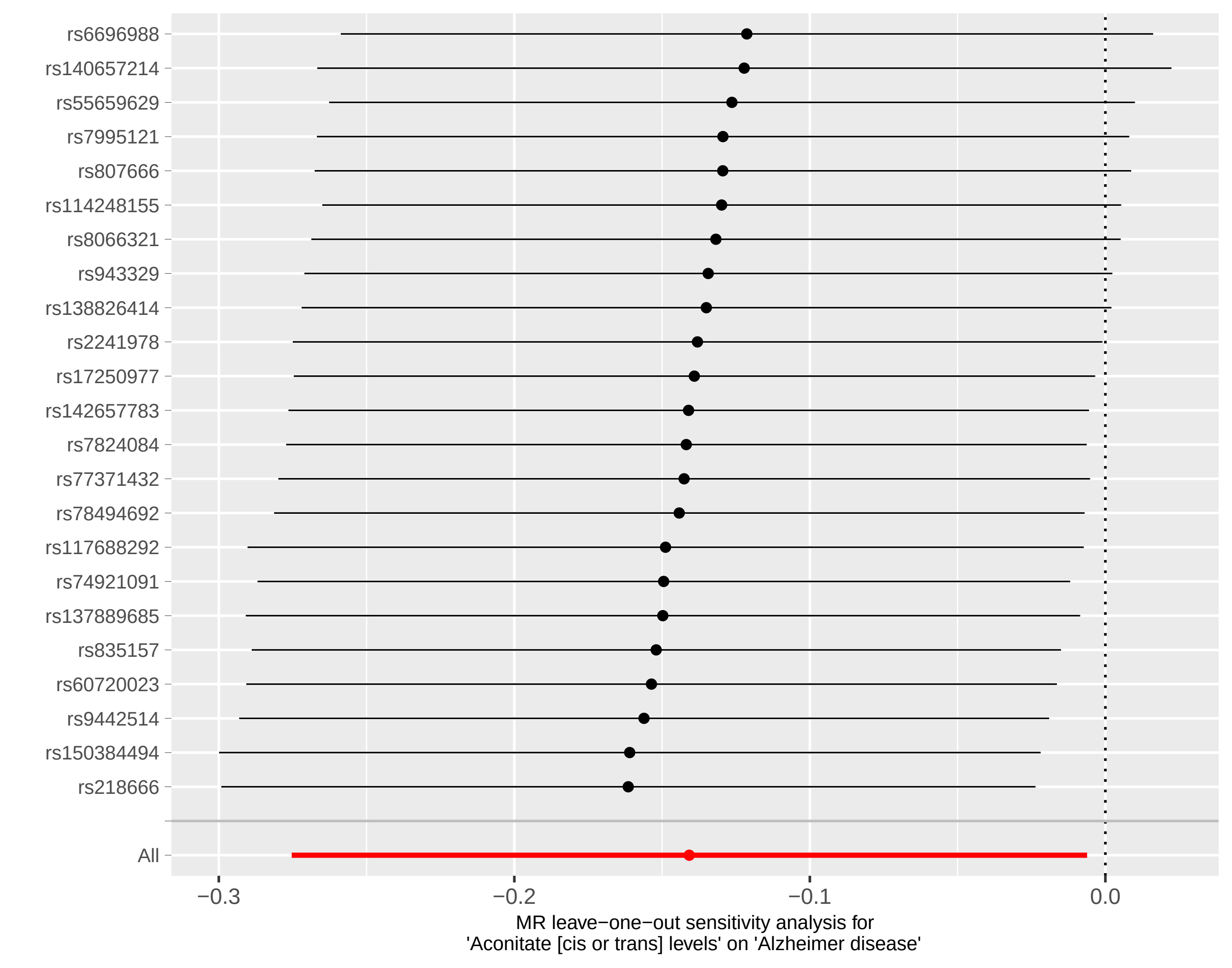
rs6696988
rs140657214
rs55659629
rs7995121
rs807666
rs114248155
rs8066321
rs943329
rs138826414
rs2241978
rs17250977
rs142657783
rs7824084
rs77371432
rs78494692
rs117688292
rs74921091
rs137889685
rs835157
rs60720023
rs9442514
rs150384494
rs218666
All
−0.3
−0.2
−0.1
0.0
MR leave−one−out sensitivity analysis for
'Aconitate [cis or trans] levels' on 'Alzheimer disease'

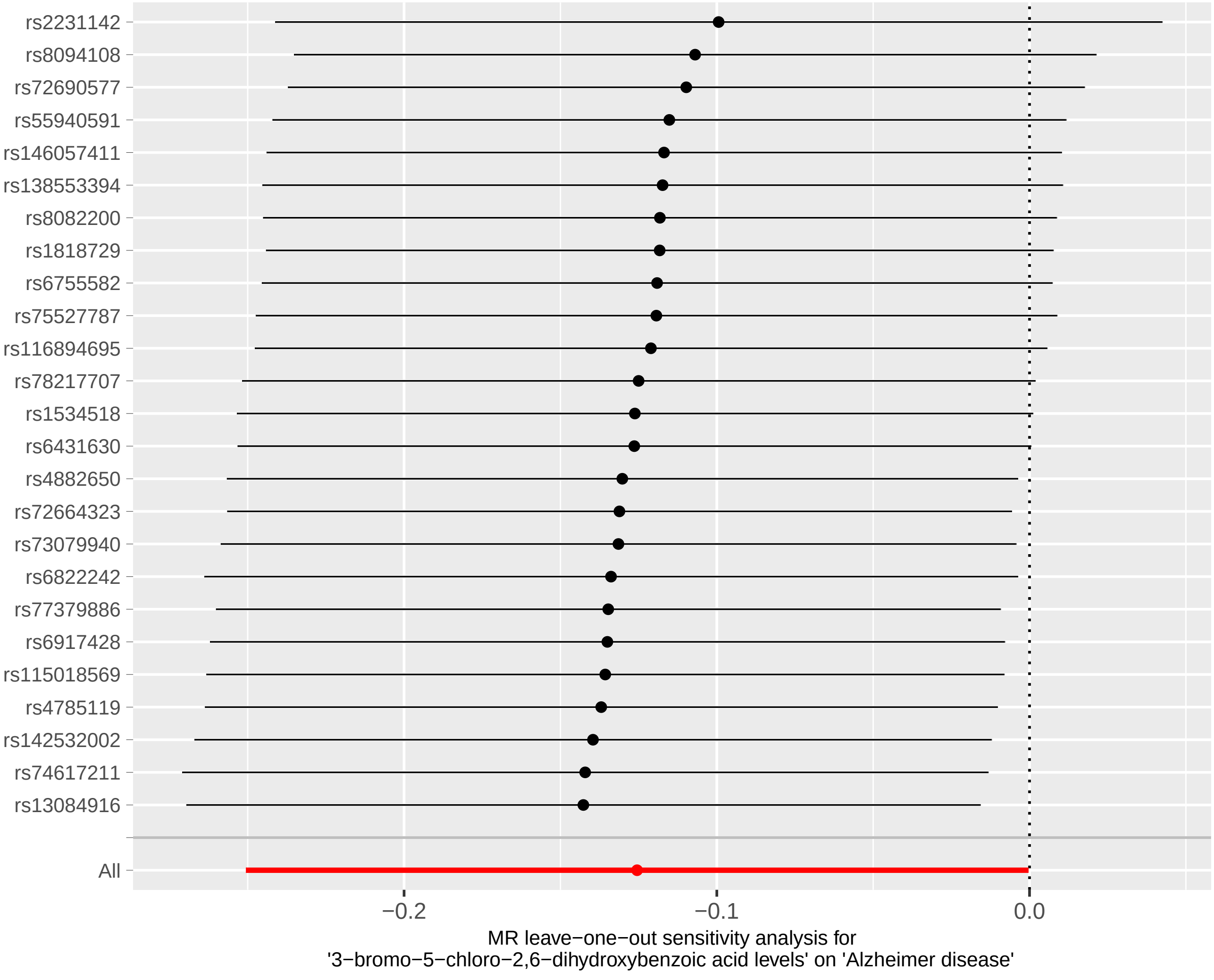
rs2231142
rs8094108
rs72690577
rs55940591
rs146057411
rs138553394
rs8082200
rs1818729
rs6755582
rs75527787
rs116894695
rs78217707
rs1534518
rs6431630
rs4882650
rs72664323
rs73079940
rs6822242
rs77379886
rs6917428
rs115018569
rs4785119
rs142532002
rs74617211
rs13084916
All
−0.2
−0.1
0.0
MR leave−one−out sensitivity analysis for
'3−bromo−5−chloro−2,6−dihydroxybenzoic acid levels' on 'Alzheimer disease'

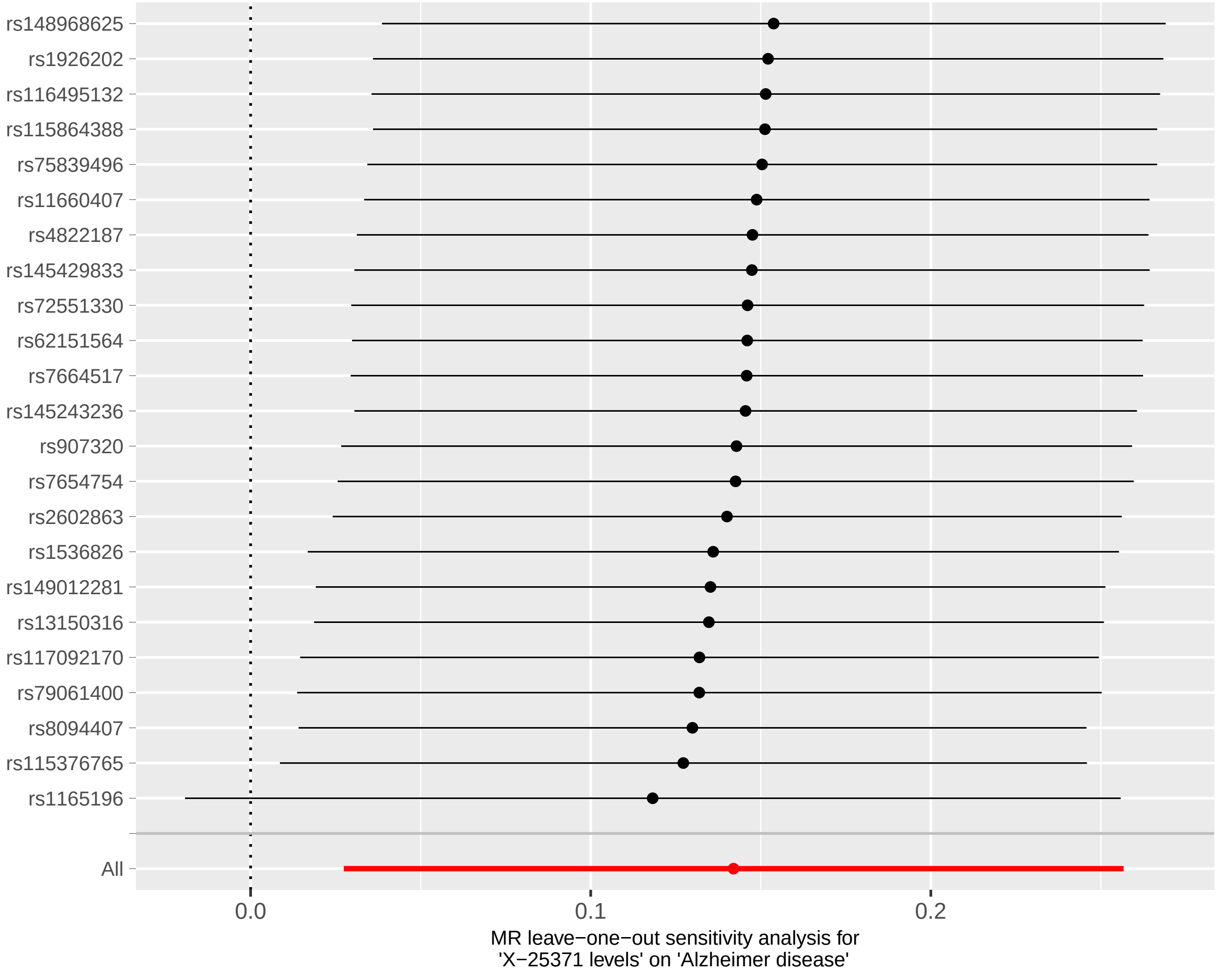

rs148968625
rs1926202
rs116495132
rs115864388
rs75839496
rs11660407
rs4822187
rs145429833
rs72551330
rs62151564
rs7664517
rs145243236
rs907320
rs7654754
rs2602863
rs1536826
rs149012281
rs13150316
rs117092170
rs79061400
rs8094407
rs115376765
rs1165196
All
0.0
0.1
0.2
MR leave−one−out sensitivity analysis for
'X−25371 levels' on 'Alzheimer disease'

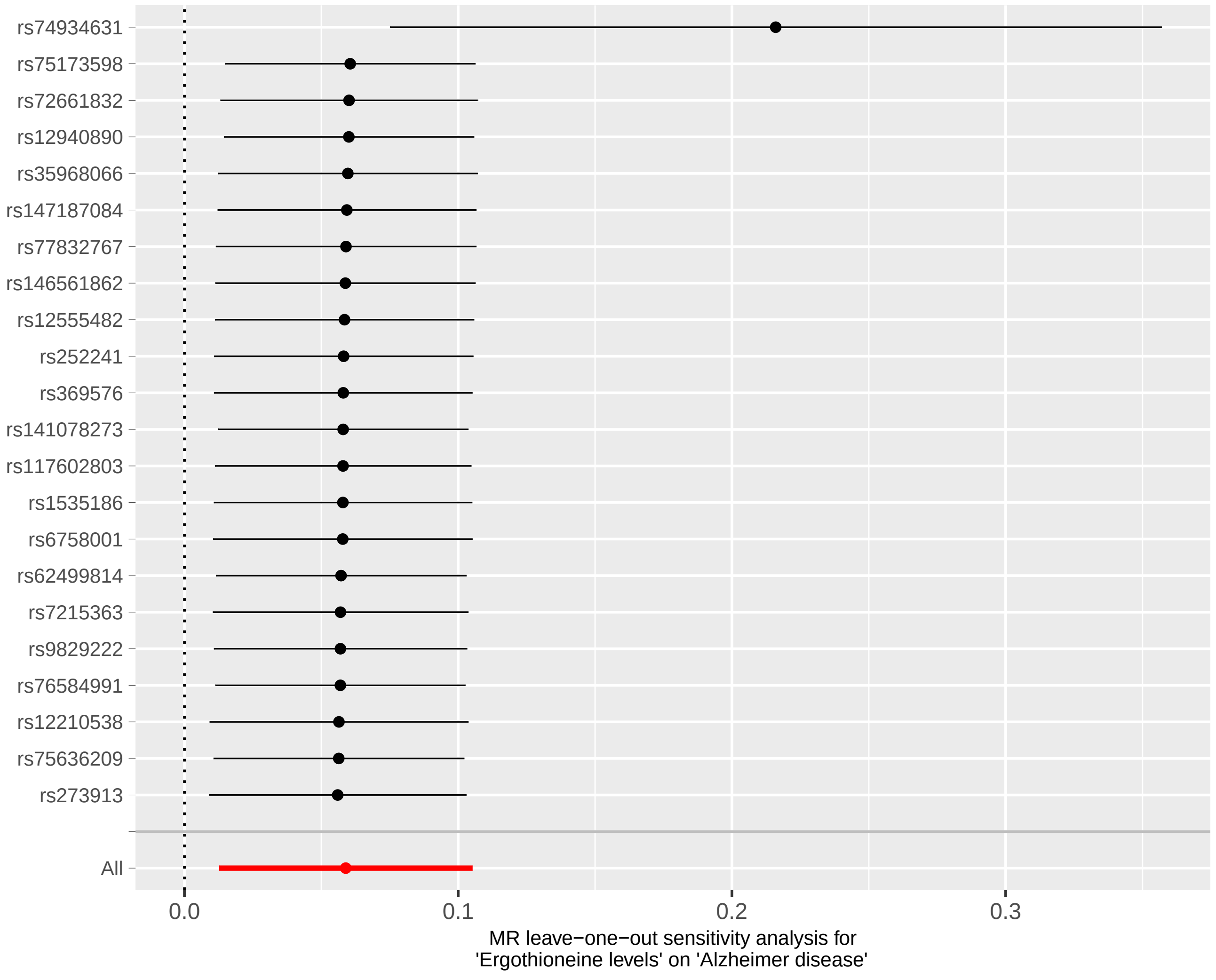

rs74934631
rs75173598
rs72661832
rs12940890
rs35968066
rs147187084
rs77832767
rs146561862
rs12555482
rs252241
rs369576
rs141078273
rs117602803
rs1535186
rs6758001
rs62499814
rs7215363
rs9829222
rs76584991
rs12210538
rs75636209
rs273913
All
0.0
0.1
0.2
0.3
MR leave−one−out sensitivity analysis for
'Ergothioneine levels' on 'Alzheimer disease'
