# Supplementary figures and images for: A multi-omics framework integrating gut microbiota, blood metabolites, and immune cells to elucidate the pathogenesis of Alzheimer’s disease
Source: Front Immunol. 2026 Jul 6;17:1842398. doi: 10.3389/fimmu.2026.1842398 (PMC13381253; doi:10.3389/fimmu.2026.1842398)

a

Before batch correction

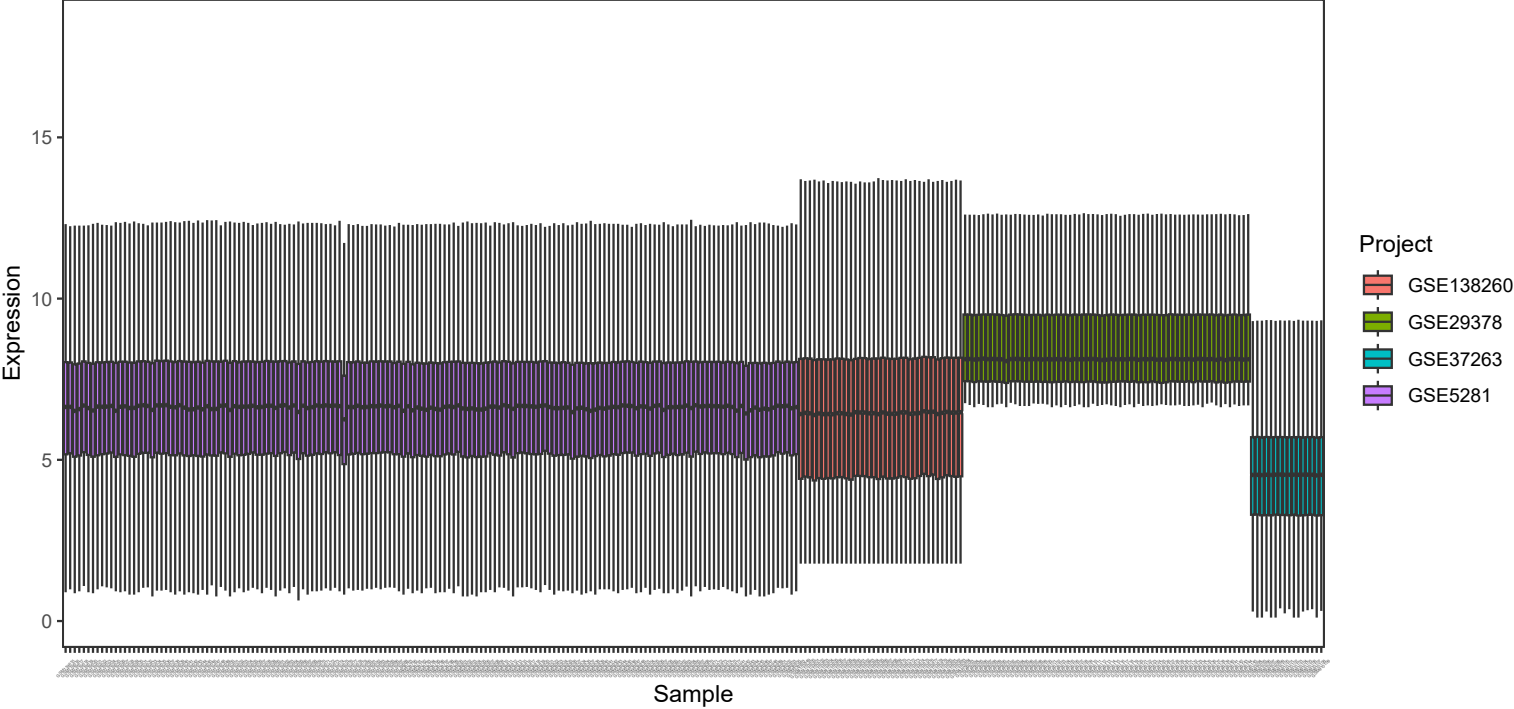

b

After batch correction

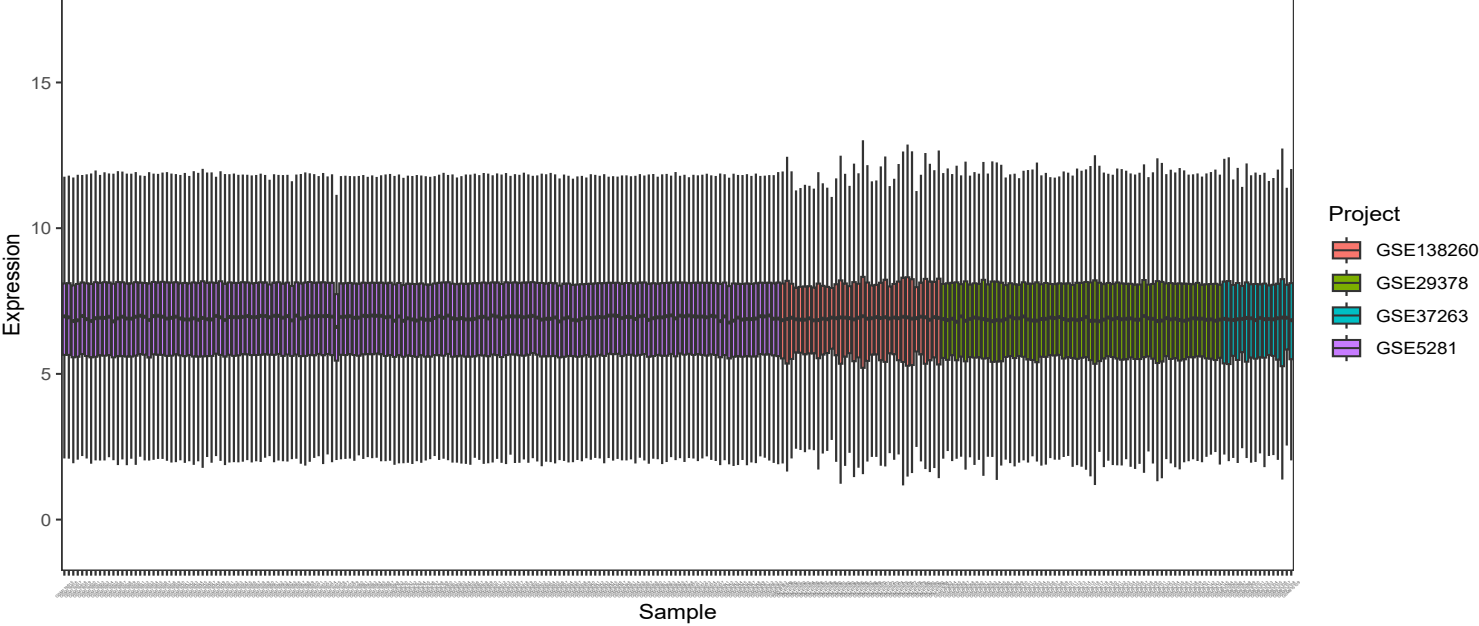

Supplement: Supplementary file 2 [file Image2.pdf]
